# Short-term Effects of High Thoracic Epidural Blockade in Patients With Ischemic Heart Disease and Heart Failure: A Systematic Review and Data Synthesis

**DOI:** 10.31083/RCM37886

**Published:** 2025-07-31

**Authors:** Daoyu Guo, Mei Chen, Chen Zhu, Yang Liu

**Affiliations:** ^1^Department of Internal Medicine and Pediatrics, Clinical College of Qilu Medical University, 255300 Zibo, Shandong, China; ^2^Department of Nursing Science, Clinical College of Qilu Medical University, 255300 Zibo, Shandong, China; ^3^Department of Cardiology, Affiliated Hospital of Qilu Medical University, 255213 Zibo, Shandong, China

**Keywords:** high thoracic epidural blockade, heart failure, ischemic heart disease, systematic review

## Abstract

**Background:**

High thoracic epidural blockade (HTEB) with local anti-sympathetic effects modulates cardiac performance in patients undergoing cardiac or non-cardiac surgeries. However, the short-term cardio-protective effects of HTEB in non-operative patients with ischemic heart disease (IHD) and heart failure (HF) remain unclear. Our study aimed to pool evidence regarding the benefits of adjunctive HTEB intervention in patients with IHD and HF.

**Methods:**

Exposures were defined as non-operative patients with IHD and HF who received adjunctive HTEB intervention and/or conventional medical treatment (CMT). The primary outcomes were clinical recovery indicator assessments, electrocardiographic and ultrasonic index improvement, laboratory tests, and hemodynamic benefits provided by adjunctive HTEB treatment. The secondary outcome was the effectiveness rate and adverse side effects after HTEB intervention. The pooled analyses of continuous variables were conducted using a fixed-effects model and the effects were represented by the weighted mean difference (WMD) and a 95% confidence interval (CI). The effective rates of HTEB treatment were represented using odds ratios (ORs, 95% CI) or effect size (ES, 95% CI). The I^2^ statistic was used to identify any inconsistency in the pooled results from individual trials. A meta-regression and subgroup analysis were conducted when inconsistencies in individual trials were detected.

**Results:**

HTEB treatment was associated with a significant 10% increase in left ventricular ejection fraction (summary WMD, 9.651 [95% CI: 9.082 to 10.220]), a decline in neuroendocrine hormone levels, myocardial ischemia relief, improvement in hemodynamics, and the reversal of decompensated cardiac remodeling. HTEB treatment is more effective than conventional medical treatment (odds ratio, 5.114 [95% CI: 3.189 to 8.203]) in treating HF and angina pectoris.

**Conclusions:**

Our results suggest that HTEB intervention may be a complementary approach for cardiac rehabilitation in patients with IHD and HF. However, more data are necessary to confirm these findings due to the significant heterogeneity of the included studies.

## 1. Introduction

Heart failure (HF) is the end-stage of multiple cardiovascular diseases and has become the leading cause of death worldwide [1]. HF is characterized by a multi-faceted syndrome with clinical symptoms and signs resulting from cardiac dysfunction, which is associated with a high risk of mortality in hospitalized patients. HF has been categorized into three phenotypes based on left ventricular ejection fraction (LVEF) measurements: HF with preserved (HFpEF: LVEF ≥50%), mildly reduced (HFmrEF: LVEF 41%–49%), and reduced ejection fraction (HFrEF: LVEF ≤40%). Epidemiological reports indicate that the 30-day mortality rate due to HF accounts for 2–3%, while the mortality rates for 1 year, 3 years, and 5 years are 15–30%, 30–50%, and 50–75%, respectively [2]. In China, a high HF mortality rate exists among patients with reduced ejection fraction (rEF). The post-discharge all-cause mortality rates for patients with HF at 30 days, 1 year, and 3 years are 2.4%, 13.7%, and 28.2%, respectively [3]. Meanwhile, progressive pathological remodeling of the left ventricle worsens cardiac function and increases the risk of death in HF patients. Delaying the progression of cardiac structural remodeling has become one of the most important goals in managing HF for hospitalized patients to improve long-term outcomes.

Several newly developed anti-HF drugs have been introduced in recent years, including mineralocorticoid receptor antagonists (MRAs), soluble guanylate cyclase (sGC) stimulators, sodium–glucose transport protein 2 inhibitors, selective vasopressin V2 receptor antagonists, novel anti-renin–angiotensin system (RAS) agents, calcium sensitizers, and the I_f_ channel inhibitor [4]. Patients diagnosed with HFrEF should receive standard pharmacological treatment, including a combination of an angiotensin-converting enzyme inhibitor (ACE-I)/angiotensin receptor–neprilysin inhibitor (ARNI), a beta-blocker, a MRA, and a sodium–glucose co-transporter 2 inhibitor (SGLT2i), which comprise the foundation of pharmacotherapy for HFrEF patients. Sacubitril/valsartan, a representative ARNI, is recommended as a first-line therapy instead of an ACE-I or angiotensin II receptor blocker (ARB) in patients with HFrEF [4]. The administration of sacubitril/valsartan has been associated with several advantageous outcomes, including improvement in symptoms and quality of life, a decrease in the occurrence of diabetes necessitating insulin therapy, a mitigation of the decline in estimated glomerular filtration rate (eGFR), as well as a lower incidence of hyperkalemia. Patients starting sacubitril/valsartan should have adequate blood pressure and an estimated GFR greater than or equal to 30 mL/min/1.73 m^2^ [4]. Beta-blockers, ACE-I/ARB/ARNI, and SGLT2i were independently associated with a lower risk of all-cause mortality in patients with HF. Nevertheless, anti-sympathetic therapy remains an important component in the comprehensive treatment of HF. Accumulating evidence suggested that exacerbated sympathetic nerve discharge and a progressive loss of rhythmic sympathetic oscillation contribute to the hyper-adrenergic state associated with HF [5]. Levine and colleagues [6] found significant correlations between plasma norepinephrine concentrations and hemodynamic evidence associated with congestive HF, while sympathetic stimulation contributed to renin release. The results by Levine *et al*. [6] implicated that the sympathetic response contributes to the hemodynamic derangement observed in HF patients. These pathophysiological changes can lead to a diminished compensatory response to increased cardiac load, ultimately facilitating cardiac enlargement, symptomatic mitral regurgitation, and systolic dysfunction [6, 7, 8]. β-adrenoceptor blockers have been shown to reduce mortality and morbidity in patients with HFrEF [4]. A previous meta-analysis of all major beta-blocker trials in HFrEF indicated no benefit on hospital admissions and mortality in the subgroup of patients with HFrEF and atrial fibrillation (AF) [9]. Meanwhile, β-blockers should be cautiously initiated for patients admitted to the hospital with acute HF once the patient has been hemodynamically stabilized [4]. Therefore, the use of β-adrenoceptor blockers is also limited due to compensatory heart rate responses and impaired cardiac output [10]. Furthermore, moxonidine, a general sympathetic inhibitor, is not recommended for treating HF, as this agent may increase the mortality rate in HF patients [11].

The impact of high thoracic epidural blockade (HTEB) with thoracic epidural anesthesia on the postoperative neurohumoral stress response and cardiovascular pathophysiology has been the focus of extensive clinical and experimental research for several years [12]. Some investigators have raised questions about the effect of HTEB on systolic left ventricular function. Results from studies on the HTEB effect have varied, indicating that it may be unchanged, impaired, or even improved in healthy individuals. Some of these studies found that HTEB potentially increased the luminal dimensions of stenotic coronary vessels, thereby reducing coronary blood flow [13]. HTEB may also help alleviate severe refractory unstable angina pectoris by improving abnormalities in coronary function, and can complement traditional medication treatments to achieve better therapeutic effects [14]. Nevertheless, recent well-designed clinical trials on HTEB intervention primarily concentrated on the anti-ischemic and antiarrhythmic properties in patients with coronary artery disease (CAD) or those undergoing surgery [12, 14]. There is limited information in the scientific literature regarding non-surgical HF patients. Some studies have found that adjunctive cardiac sympathetic blockade produces favorable hemodynamic effects during rehabilitation management for HF; meanwhile, the combination of HTEB and conventional medical treatment (CMT) also demonstrated superior therapeutic effects in hospitalized patients with dilated cardiomyopathy [15, 16]. Regrettably, compared to well-designed clinical trials conducted recently, earlier studies did not strictly adhere to randomized principles and exhibited varying degrees of flaws in their design. Additionally, since most relevant results were published in Chinese, these data received limited attention and were not widely recognized. However, these findings may provide important insights into the neuroregulation of HF and the changes in cardiac performance following local cardiac sympathetic blockage in the context of abnormal hemodynamics associated with HF. Therefore, the current study aimed to consolidate evidence from published articles regarding the effectiveness of HTEB for managing ischemic heart disease (IHD) and HF.

## 2. Materials and Methods

The methods applied in this study adhered to the Preferred Reporting Items for Systematic Reviews and Meta-Analyses (PRISMA) guidelines [17]. The review protocol was not registered.

### 2.1 Search Strategy 

We searched the PubMed, Web of Science, Embase, and Chinese National Knowledge Infrastructure (CNKI) databases. The Medical Subject Headings (MeSH) terms and keywords included “thoracic epidural blockade”, “cardiac sympathetic blockade”, “thoracic epidural analgesia”, “epidural anesthesia”, “epidural analgesia”, “cardiac sympathectomy”, “heart failure”, “ischemic heart disease”, “dilated cardiomyopathy”, “angina”, “coronary artery disease”, “left ventricular ejection fraction”, and “left ventricular function”. These terms were used to conduct an integrative search of the aforementioned databases. The search strategy is detailed in **Supplementary Table 1**.

### 2.2 Selection Criteria

Clinical trials were identified from systematic literature searches in the databases mentioned above. The cardiac diseases involved IHD with or without HF, and HF due to various cardiovascular disorders. The diagnostic criteria of HF and other types of cardiac diseases were based on the World Health Organization (WHO) guidelines and the Chinese Guidelines for Diagnosis and Treatment of HF cited in the included studies. To reduce the bias of the selected studies, we also screened valuable conference abstracts or published supplementary issues on HTEB treatment. The languages of publication included, but were not restricted to, English and Chinese. We included two types of clinical trials: case–control trials and case–series reports; two groups of patients were noted in the case–control trials. One group of patients received a combination of HTEB and CMT treatment, whereas the other group only received CMT treatment. The trials also described comparing cardiac outcomes between the two groups of patients before and after treatment. In the case–series reports, the patients received combinations of HTEB and CMT treatments with no control arm, and the comparisons of cardiac outcomes were conducted pre- and post-treatment. We excluded the isolated case reports, HTEB combined with general anesthesia, and those undergoing cardiac or other surgery.

### 2.3 Data Extraction and Quality Assessment

Two independent reviewers (DYG and MC) extracted data based on the baseline characteristics of each patient, study design, and treatment or control agents. Another author assisted with the data extraction (CZ). The quality of the individual case–control articles (randomized assignment) was assessed according to the Jadad score [18]. The Newcastle–Ottawa Scale (NOS) was added for additional evaluation of the case–control trials [19]. The case–series studies were evaluated using the developing tool from the Institute of Health Economics (IHE) [20]. This tool contains 21 independent items that assess the quality of the case reports. Any discrepancies were resolved through consensus. If a consensus could not be reached, the author (YL) decided on data extraction and trial eligibility. The protocol for this analysis was not registered.

### 2.4 Short-term Outcomes

#### 2.4.1 Case–Control Trials

Clinical indicators included heart rate (HR), systolic blood pressure (SBP), diastolic blood pressure (DBP), mean arterial blood pressure (MBP), and the New York Heart Association (NYHA) cardiac functional classification.

Ultrasound indicators: LVEF, left ventricular fraction shortening (LVFS), left ventricular end-systolic dimension (LVESD), left ventricular end-diastolic dimension (LVEDD), left atrial dimension (LAD), left ventricular end-diastolic volume (LVEDV), left ventricular end-systolic volume (LVESV), left ventricular weight (LVW), right ventricle transverse dimension (RVTD), right atrium end-diastolic transverse dimension (RATD), MRA, tricuspid regurgitation area (TRA), pulmonary arterial pressure (PAP), and cardiac index.

Curative effect: total effective rate, excellence rate, and inefficiency rate.

#### 2.4.2 Case–Series Studies

Clinical indicators: frequency of ischemic episodes, duration of ischemic episodes, mean HR, SBP, DBP, MBP, NYHA cardiac functional classification, and 6-minute walk distance test (6-MWD).

Cardio-electronic indicators: ΣST-T, NST-T, Q-T dispersion, Q-T corrected dispersion, J-T dispersion, autonomic neural function: standard deviation of NN intervals (SDNN) and standard deviation of the average NN intervals (SDANN) (24-hour dynamic electrocardiogram: Holter).

Radiological indicator: Cardiothoracic ratio (CTR).

Ultrasound indicators: LVEF, LVFS, LAD, LVEDD, LVESV, LVEDV, A/E peaking ratio, and E/A peaking ratio. Laboratory indicators: plasma N-terminal pro-B-type natriuretic peptide (NT-pro BNP), serum angiotensin-converting enzyme 2 (Ang II), and norepinephrine (NE).

Hemodynamic indicators: cardiac output (CO), stroke volume (SV), cardiac index, mean pulmonary arterial pressure (mPAP), pulmonary capillary wedge (PCW), pulmonary vascular resistance indices (PVRIs), stroke volume index (SVI), and systemic vascular resistance indices (SVRIs).

Curative effect: total effective rate, excellence rate, and inefficiency rate.

#### 2.4.3 Merged Studies (HTEB Group of 25 Case–Control Trials and 38 Case–Series Studies)

Clinical indicators: HR, SBP, DBP, MBP, and the NYHA cardiac functional classification.

Ultrasound indicators: LVEF, LVFS, LAD, LVEDD, LVESV, LVEDV, and CI.

Curative effect: total effective rate, excellence rate, and inefficiency rate.

### 2.5 Estimation of HTEB-Related Adverse Events

The incidence of adverse events related to HTEB was assessed in the individual studies, and the percentage of each event is reported.

### 2.6 Data Analysis 

The primary data of the NYHA cardiac functional classification in some of the included studies was reported as the difference in the mean (M) ± standard deviation (SD) between the HTEB and CMT groups, as well as before and after HTEB or CMT treatment. Other studies reported the NYHA functional classification as the grade (1, 2, 3, and 4) numbers before and after HTEB or CMT treatment. We reproduced the comparative methods in each study to obtain the corresponding M ± SD values for the NYHA functional classification. The results were analyzed using a two-tailed independent *t-*test in SPSS software (version 20.0, SPSS Inc., Chicago, IL, USA) to obtain the reliable M ± SD values for NYHA functional classification in the meta-analysis of continuous variables. The data were prepared and are presented as the M ± SD for the following pooled analysis (**Supplementary Tables 2** and **3**). The BP transformations are reported according to the formula:

(1) 1 kpa ≈ 7.5 mmHg

The reported hemodynamic indices measured using cardiac catheterization were converted according to the formula:

(2) ⁢1 ⁢m⁢m⁢H⁢g⋅min⋅L-1⁢( Wood )=80 ⁢ dynes ⋅sec-1⋅cm-5

A continuous variable in individual studies was presented as the weighted mean difference (WMD) with a 95% confidence interval (CI) when comparing adjunctive HTEB treatment to CMT alone in 24 case–control trials, as well as the change in outcomes pre- and post-HTEB treatment in 38 case–series studies. The effective rate was analyzed using a fixed-effect model and a random-effects model. When high heterogeneity existed between the adjunctive HTEB and CMT groups of individual examinations in 24 case–control trials, the summary ORs and 95% CIs were applied to represent the results. In analyzing 38 case–series and merged studies, a single rate meta-analysis was conducted using the representative effect size (ES) and 95% CI. The merged analyses (HTEB groups of case–control trials and case–series studies) were conducted by comparing the main parameters between pre- and post-adjunctive HTEB treatment. When high heterogeneity existed, the pooled effects were calculated using a fixed-effect model and a random-effects model.

Inconsistency among the eligible studies was detected using the I^2^ statistic [21]. Moreover, meta-regression of covariates and a subgroup analysis were conducted when significant heterogeneity was identified. Potential publication bias was evaluated using the Egger’s test for case–control trials and case–series studies. Results were deemed statistically significant at a *p*-value of less than 0.05. The analytical procedure was achieved using STATA 12.0 software (StataCorp, College Station, TX, USA).

## 3. Results

### 3.1 Study Selection

A total of 25 case–control trials [14, 16, 22, 23, 24, 25, 26, 27, 28, 29, 30, 31, 32, 33, 34, 35, 36, 37, 38, 39, 40, 41, 42, 43, 44] and 38 case–series studies [13, 45, 46, 47, 48, 49, 50, 51, 52, 53, 54, 55, 56, 57, 58, 59, 60, 61, 62, 63, 64, 65, 66, 67, 68, 69, 70, 71, 72, 73, 74, 75, 76, 77, 78, 79, 80, 81] were included. The case–control trials included 23 random assignment trials [14, 16, 23, 24, 25, 26, 27, 28, 29, 30, 31, 32, 33, 34, 35, 36, 37, 38, 39, 40, 41, 42, 44], one observational study [22], and one retrospective study [43]. In the case–control trials, there were eight trials on dilated cardiomyopathy [14, 22, 25, 26, 29, 31, 32, 33], four trials on ischemic cardiomyopathy [35, 37, 41, 43], two trials on myocardial infarction [30, 40], five trials on angina pectoris [14, 23, 24, 38, 39], four trials on multiple cardiovascular disease [27, 28, 42, 44], and one trial on peripartum cardiomyopathy [36].

**Fig. 1.**
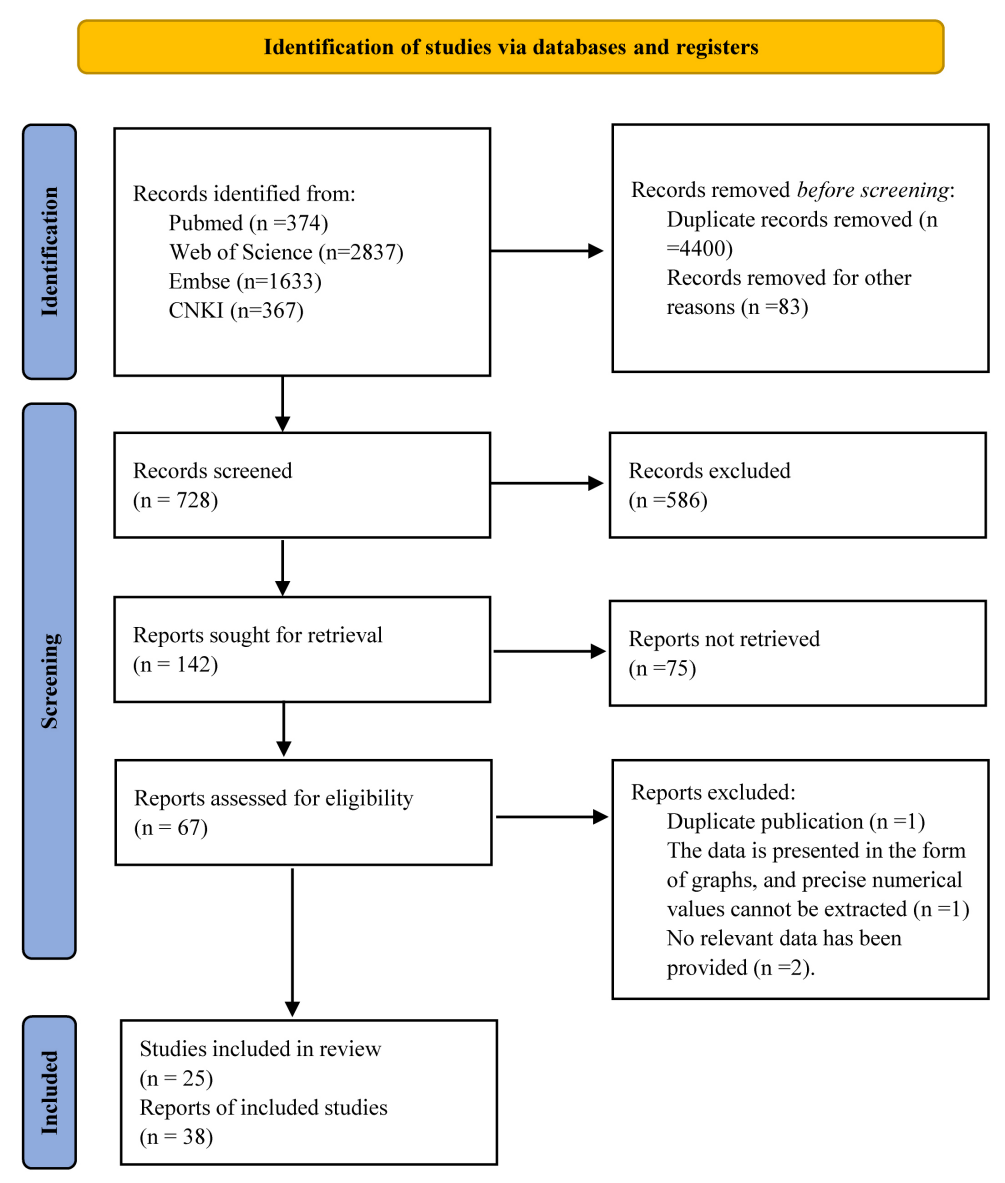
**Preferred Reporting Items for Systematic Reviews and Meta-Analyses (PRISMA) flowchart and analytical protocol**. CNKI, Chinese National Knowledge Infrastructure.

Additionally, we included 38 case–series studies to evaluate additional cardiac indicators in patients receiving adjunctive HTEB treatment; these included nineteen reports on coronary artery disease [13, 45, 46, 47, 48, 49, 50, 51, 52, 53, 54, 55, 57, 60, 62, 68, 69, 70, 71], seven reports on dilated cardiomyopathy [56, 58, 59, 61, 63, 67, 81], one report on Keshan disease (endemic cardiomyopathy) [64], one report on alcoholic cardiomyopathy [65], one report on familial dilated cardiomyopathy [66], two reports on diabetic cardiomyopathy or HF with type II diabetes [72, 73], four reports on multiple cardiovascular diseases [74, 75, 78, 79], one report on HF with AF [76], one report on hypertrophic cardiomyopathy [77], and two reports on valvular heart diseases [79, 80]. Fig. 1 presents the PRISMA selection flowchart and the analytical protocol. **Supplementary Table 4** outlines the clinical characteristics of patients in individual studies. **Supplementary Table 5 **presents the deciphered therapeutic methods.

### 3.2 Quality Assessment 

A total of 25 case–control trials reported randomization allocation, but only two trials were strictly adherent (**Supplementary Table 6**) [42, 44]. Therefore, we added the NOS, a tool for evaluating the risk of bias in non-randomized studies, which can provide more information on the study methods. The total score of the 24 studies was estimated to be up to six points [14, 22, 23, 24, 25, 26, 27, 28, 29, 30, 31, 32, 33, 34, 35, 36, 37, 38, 39, 40, 41, 42, 43, 44], with only one study estimated to be up to eight points (**Supplementary Tables 7** and **8**) [16]. All but one (Chi *et al*. [16], 2011) case–control study lacked the adequacy of follow-up of cohorts. Meanwhile, the case–series studies were estimated using modified tools developed by the IHE. A total of 21 IHE items are listed in **Supplementary Table 9**. Most of the case–series studies fulfilled items 1, 2, 5 to 12, 15, and 17; a few studies met items 3, 4, 13, 14, 16, 18, and 19 (**Supplementary Table 10**). In total, 24 studies used β-blockers, [13, 14, 23, 24, 27, 31, 34, 35, 38, 39, 42, 44, 45, 46, 47, 48, 57, 60, 62, 69, 70, 76, 77, 81], 14 used angiotensin converting enzyme inhibitors or/and angiotensin receptor antagonists [13, 23, 24, 28, 35, 41, 42, 44, 48, 71, 75, 76, 80, 81], and five studies used mineralocorticoid receptor antagonists [44, 75, 76, 80, 81].

### 3.3 Publication Bias

**Fig. 2.**
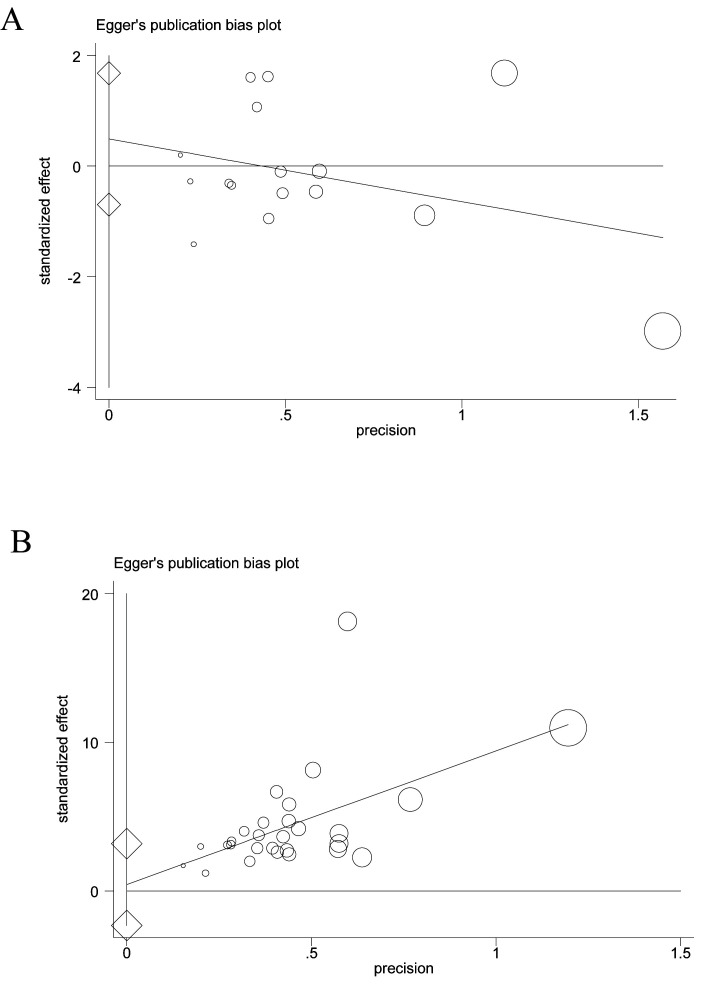
**An estimation of the publication bias based on the left ventricular ejection fraction (LVEF) effect by the Egger’s test**. (A) Case–control studies, (B) case–series studies.

Based on the pretreatment effects of LVEF (16 studies) and LVEDD (16 studies) between the two groups in the 25 case–control studies, the Egger’s test indicated that there was no significant publication bias for LVEF (coefficient: 0.494, 95% CI: –0.695 to 1.683; *p* > |*t*| = 0.388) (Fig. 2A). Likewise, the Egger’s test on LVEDD indicated no significant publication bias (coefficient: 0.348, 95% CI: –0.838 to 1.534; *p* > |*t*| = 0.539) (**Supplementary Fig. 1A**). Next, we estimated the publication bias in 38 non-control case–series studies using the same two indicators, LVEF and LVEDD. Similarly, the results of the Egger’s tests on the LVEF (18 studies) indicated no significant publication bias (coefficient: 0.430, 95% CI: –2.318 to 3.179; *p* > |*t*| = 0.750) among the involved studies (Fig. 2B). Nonetheless, we performed an additional sensitivity analysis for LVEF. One study, Chi *et al*. [16], exhibited deviation with an extended confidence interval (**Supplementary Fig. 1B**,**C**). Although this study did not impact the final estimated publication bias result for LVEF (coefficient: 0.388, 95% CI: –2.382 to 3.159; *p* > |*t*| = 0.775), we omitted this study and re-analyzed the publication bias (**Supplementary Fig. 1D**). Comparatively, publication bias could not be prevented when estimating the LVEDD (17 studies) (coefficient: 3.371, 95% CI: –1.573 to 5.169; *p* > |*t*| = 0.001). Therefore, we conducted a sensitivity analysis for LVEDD and found that one study, Liu *et al*. [58], presented a deviation with an extended confidence interval. Subsequently, no significant publication bias was observed for LVEDD after this study was omitted (coefficient: –0.215, 95% CI: –1.652 to 1.223; *p* > |*t*| = 0.758) (**Supplementary Fig. 1E,F**).

### 3.4 Safety Information of HTEB in Individual Studies

Complications were estimated in 1818 patients undergoing an HTEB intervention. Hypotension (0.39%), feeling weak (0.61%), dizziness (0.55%), infection (0.17%), bleeding (0.11%), local inflammation (0.28%), catheter detachment or occlusion (0.61%), pain (0.17%), Horner’s syndrome (0.22%), and urinary retention (0.55%) were reported as complications related to HTEB. Cardiac arrest or mortality (0.17%) was unrelated to HTEB intervention (**Supplementary Table 11**). No severe complications, such as respiratory depression, hematomas, thromboembolism, cardiac arrest, or death, were reported after HTEB treatment in the reviewed studies.

### 3.5 Analysis of Case–Control Studies

No significant difference was observed in the pooled effects of indicators between pre-HTEB treatment and pre-CMT. HTEB treatment was associated with a significant reduction in HR, blood pressure (BP), and NYHA cardiac functional classification (WMD = –0.602 grade, 95% CI: –0.691 to –0.513, *p* = 0.000, I^2^ = 87.4%) compared to CMT. HTEB treatment significantly improved cardiac function, including LVEF (WMD = 5.589%, 95% CI: 4.727 to 6.451, *p* = 0.000, I^2^ = 62.2%), LVFS (WMD = 0.799%, 95% CI: 0.587 to 1.011, *p* = 0.000, I^2^ = 45.4%), and CI (WMD = 0.372 L min^-1^ m^-2^, 95% CI: 0.305 to 0.438, *p* = 0.000, I^2^ = 0%). In contrast, HTEB treatment reduced cardiac compensative expansion, LAD (WMD = –2.604 mm, 95% CI: –3.719 to –1.488, *p* = 0.000, I^2^ = 0.9%), LVESD (WMD = –4.114 mm, 95% CI: –6.954 to –1.275, *p* = 0.005, I^2^ = 0.0%), LVEDD (WMD = –3.593 mm, 95% CI: –4.432 to –2.754, *p* = 0.000, I^2^ = 22.8%), LVESV (WMD = –37.601 mL, 95% CI: –52.050 to –23.153, *p* = 0.000, I^2^ = 66.8%), LVEDV (WMD = –30.072 mL, 95% CI: –50.169 to –9.975, *p* = 0.003, I^2^ = 0.0%), LVW (WMD = –41.002 g/m^2^, 95% CI: –51.014 to –30.990, *p* = 0.000, I^2^ = 21.6%), and mitral regurgitation area (WMD = –1.734 mm^2^, 95% CI: –2.810 to –0.658, *p* = 0.002, I^2^ = 87.4%) compared to CMT alone. HTEB also alleviated right heart expansion (RATD: WMD = –2.926, 95% CI: –4.585 to –1.266, *p* = 0.001, I^2^ = 51.2%; RVTD: WMD = –5.149, 95% CI: –6.807 to –3.491, *p* = 0.000, I^2^ = 79.7%), tricuspid regurgitation area (WMD = –2.866 mm^2^, 95% CI: –3.914 to –1.819, *p* = 0.000, I^2^ = 92.1%), and pulmonary arterial pressure (WMD = –17.196 mmHg, 95% CI: –22.036 to –12.356, *p* = 0.000, I^2^ = 0.0%). HETB treatment significantly decreased plasma BNP concentrations (WMD = –210.429 pg/mL or ng/L, 95% CI: –217.478 to –203.380, *p* = 0.000; I^2^ = 80.2%) compared to CMT alone. Due to the significant heterogeneities observed in the pooled results of indicators among the included literature, indicated by the fixed-effects model, we included a random-effects model to reanalyze these results, particularly for studies with an I^2^ greater than 75%. Consistent results were observed from the random-effects model, excluding the indicators, mean arterial blood pressure (*p* for effect >0.05), mitral regurgitation area (*p* for effect >0.05), and tricuspid regurgitation area (*p* for effect >0.05).

The pooled results indicated that HTEB treatment is more efficient than CMT alone, as the total effective rate: OR, 5.114 (95% CI: 3.189 to 8.203), *p* = 0.000, I^2^ = 0%, and the excellence rate: OR, 2.828 (95% CI: 1.968 to 4.063), *p* = 0.000, I^2^ = 0%. HTEB treatment was less inefficient than CMT alone (OR, 0.186 [95% CI: 0.112 to 0.307], *p* = 0.000, I^2^ = 0%). The detailed results are presented in Table 1.

**Table 1.** **Pooled short-term effects of adjunctive HTEB intervention on cardiac function and structure compared to baseline and conventional medical treatments**.

Parameters		Number of studies	Pooled weighted mean difference (WMD) and 95% confidence interval	*p*-value for WMD	I-squared (I^2^, %)	*p*-value for heterogeneity
Heart rate (times per min)						
	Post-HTEB versus pre-HTEB	8	–12.197 (–13.328, –11.066)	0.000	94.4%	0.000
	*Random-effects model*		–12.126 (–17.348, –6.904)	0.000	94.4%	0.000
	Post-CMT versus pre-CMT	7	–6.681 (–8.211, –5.152)	0.000	79.1%	0.000
	*Random-effects model*		–5.795 (–9.652, –1.937)	0.003	79.1%	0.000
	Pre-HTEB versus pre-CMT	7	1.213 (–0.501, 2.926)	0.766	0.0%	0.166
	Post-HTEB versus post-CMT	9	–7.851 (–8.556, –7.147)	0.000	84.3%	0.000
	*Random-effects model*		–7.347 (–9.646, –5.047)	0.000	84.3%	0.000
Systolic blood pressure (mmHg)						
	Post-HTEB versus pre-HTEB	6	–18.494 (–20.632, –16.356)	0.000	94.7%	0.000
	*Random-effects model*		–12.082 (–22.307, –1.858)	0.021	94.7%	0.000
	Post-CMT versus pre-CMT	5	–6.915 (–10.028, –3.802)	0.000	91.3%	0.000
	*Random-effects model*		–1.824 (–13.664, 10.017)	0.763	91.3%	0.000
	Pre-HTEB versus pre-CMT	4	2.568 (–0.461, 5.597)	0.986	0.0%	0.097
	Post-HTEB versus post-CMT	7	–3.241 (–4.867, –1.615)	0.000	92.8%	0.000
	*Random-effects model*		–8.030 (–15.257, –0.802)	0.029	92.8%	0.000
Diastolic blood pressure (mmHg)						
	Post-HTEB versus pre-HTEB	5	–7.767 (–9.364, –6.169)	0.000	92.1%	0.000
	*Random-effects model*		–7.169 (–13.205, –1.133)	0.020	92.1%	0.000
	Post-CMT versus pre-CMT	4	–4.712 (–6.153, –3.270)	0.005	69.9%	0.000
	Pre-HTEB versus pre-CMT	4	3.503 (1.313, 5.693)	0.002	67.0%	0.028
	Post-HTEB versus post-CMT	6	0.861 (0.087, 1.636)	0.029	96.0%	0.000
	*Random-effects model*		–5.170 (–10.240, –0.100)	0.046	96.0%	0.000
Mean arterial blood pressure (mmHg)						
	Post-HTEB versus pre-HTEB	2	–9.975 (–13.747, –6.203)	0.000	86.8%	0.006
	*Random-effects model*		–8.629 (–19.451, 2.192)	0.118	86.8%	0.006
	Post-CMT versus pre-CMT	2	–1.725 (–5.822, 2.373)	0.409	0.0%	0.623
	Pre-HTEB versus pre-CMT	2	–0.061 (–3.784, 3.662)	0.974	0.0%	0.450
	Post-HTEB versus post-CMT	2	–7.992 (–12.154, –3.829)	0.000	81.8%	0.019
	*Random-effects model*		–7.098 (–17.087, 2.890)	0.164	81.8%	0.019
NYHA cardiac functional classification (grade)						
	Post-HTEB versus pre-HTEB	9	–1.319 (–1.409, –1.228)	0.000	84.4%	0.000
	*Random-effect model*		–1.386 (–1.626, –1.147)	0.000	84.4%	0.000
	Post-CMT versus pre-CMT	9	–0.647 (–0.747, –0.546)	0.000	70.5%	0.001
	Pre-HTEB versus pre-CMT	9	–0.019 (–0.108, 0.070)	0.678	0.0%	0.972
	Post-HTEB versus post-CMT	10	–0.602 (–0.691, –0.513)	0.000	87.4%	0.000
	*Random-effects model*		–0.783 (–1.052, –0.513)	0.000	87.4%	0.000
Left ventricular ejection fraction (%)						
	Post-HTEB versus pre-HTEB	16	9.472 (8.616, 10.328)	0.000	77.8%	0.000
	*Random-effects model*		9.776 (7.726, 11.825)	0.000	77.8%	0.000
	Post-CMT versus pre-CMT	16	3.763 (2.998, 4.529)	0.000	44.8%	0.027
	Pre-HTEB versus pre-CMT	16	–0.499 (–1.249, 0.251)	0.192	28.1%	0.141
	Post-HTEB versus post-CMT	16	5.589 (4.727, 6.451)	0.000	62.2%	0.001
Left ventricular fraction shortening (%)						
	Post-HTEB versus pre-HTEB	8	1.270 (1.053, 1.486)	0.000	21.6%	0.258
	Post-CMT versus pre-CMT	8	0.364 (0.154, 0.574)	0.001	0.0%	0.595
	Pre-HTEB versus pre-CMT	8	–0.109 (–0.311, 0.094)	0.293	0.0%	0.700
	Post-HTEB versus post-CMT	8	0.799 (0.587, 1.011)	0.000	45.4%	0.077
Cardiac index (L min^–⁢1^ m^–⁢2^)						
	Post-HTEB versus pre-HTEB	3	0.633 (0.549, 0.717)	0.000	92.0%	0.000
	*Random-effects model*		0.950 (0.313, 1.587)	0.003	92.0%	0.000
	Post-CMT versus pre-CMT	3	0.231 (0.151, 0.310)	0.000	91.7%	0.000
	*Random-effects model*		0.545 (0.010, 1.081)	0.046	91.7%	0.000
	Pre-HTEB versus pre-CMT	3	–0.051 (–0.146, 0.044)	0.295	0.0%	0.867
	Post-HTEB versus post-CMT	3	0.372 (0.305, 0.438)	0.000	0.0%	0.446
Left atrial dimension (mm)						
	Post-HTEB versus pre-HTEB	8	–4.454 (–5.602, –3.306)	0.000	0.0%	0.667
	Post-CMT versus pre-CMT	8	–1.260 (–2.397, –0.123)	0.030	0.0%	0.978
	Pre-HTEB versus pre-CMT	8	0.450 (–0.738, 1.637)	0.458	0.0%	0.778
	Post-HTEB versus post-CMT	8	–2.604 (–3.719, –1.488)	0.000	0.9%	0.422
Left ventricular end-systolic dimension (mm)						
	Post-HTEB versus pre-HTEB	2	–5.772 (–8.692, –2.853)	0.000	0.0%	0.830
	Post-CMT versus pre-CMT	2	–1.743 (–4.642, 1.156)	0.239	0.0%	0.417
	Pre-HTEB versus pre-CMT	2	–0.228 (–3.153, 2.697)	0.879	0.0%	0.883
	Post-HTEB versus post-CMT	2	–4.114 (–6.954, –1.275)	0.005	0.0%	0.647
Left ventricular end-diastolic dimension (mm)						
	Post-HTEB versus pre-HTEB	16	–5.341 (–6.190, –4.492)	0.000	31.1%	0.114
	Post-CMT versus pre-CMT	16	–0.980 (–1.915, –0.044)	0.040	0.0%	0.764
	Pre-HTEB versus pre-CMT	16	0.768 (–0.182, 1.719)	0.113	0.0%	0.999
	Post-HTEB versus post-CMT	15	–3.593 (–4.432, –2.754)	0.000	22.8%	0.195
Left ventricular end-systolic volume (mL)						
	Post-HTEB versus pre-HTEB	3	–42.022 (–57.831, –26.213)	0.000	55.7%	0.105
	Post-CMT versus pre-CMT	3	–5.238 (–20.319, 9.842)	0.496	0.0%	0.951
	Pre-HTEB versus pre-CMT	3	1.136 (–15.309, 17.581)	0.892	0.0%	0.959
	Post-HTEB versus post-CMT	3	–37.601 (–52.050, –23.153)	0.000	66.8%	0.049
Left ventricular end-diastolic volume (mL)						
	Post-HTEB versus pre-HTEB	3	–36.659 (–57.129, –16.190)	0.000	14.5%	0.310
	Post-CMT versus pre-CMT	3	–1.096 (–21.118, 18.926)	0.915	0.0%	0.976
	Pre-HTEB versus pre-CMT	3	4.779 (–15.711, 25.269)	0.648	0.0%	0.963
	Post-HTEB versus post-CMT	3	–30.072 (–50.169, –9.975)	0.003	0.0%	0.375
Left ventricular weight (g/m^2^)						
	Post-HTEB versus pre-HTEB	3	–42.607 (–55.360, –29.855)	0.000	16.2%	0.303
	Post-CMT versus pre-CMT	3	–8.429 (–17.494, 0.636)	0.068	0.0%	0.596
	Pre-HTEB versus pre-CMT	3	–4.587 (–16.676, 7.502)	0.457	0.0%	0.858
	Post-HTEB versus post-CMT	3	–41.002 (–51.014, –30.990)	0.000	21.6%	0.279
Mitral regurgitation area (mm^2^)						
	Post-HTEB versus pre-HTEB	2	–3.016 (–4.031, –2.000)	0.005	87.4%	0.000
	*Random-effects model*		–4.007 (–7.622, –0.392)	0.030	87.4%	0.005
	Post-CMT versus pre-CMT	2	–0.776 (–1.964, 0.412)	0.201	0.0%	0.444
	Pre-HTEB versus pre-CMT	2	0.487 (–0.649, 1.622)	0.401	0.0%	0.331
	Post-HTEB versus post-CMT	2	–1.734 (–2.810, –0.658)	0.002	87.4%	0.005
	*Random-effects model*		–2.841 (–6.748, 1.067)	0.154	87.4%	0.005
Right atrium end-diastolic transverse dimension (mm)						
	Post-HTEB versus pre-HTEB	3	–4.112 (–5.873, –2.351)	0.000	40.2%	0.188
	Post-CMT versus pre-CMT	3	–1.505 (–3.041, 0.031)	0.055	0.0%	0.866
	Pre-HTEB versus pre-CMT	3	0.054 (–1.680, 1.788)	0.951	0.0%	0.378
	Post-HTEB versus post-CMT	3	–2.926 (–4.585, –1.266)	0.001	51.2%	0.129
Right ventricle transverse dimension (mm)						
	Post-HTEB versus pre-HTEB	3	–4.663 (–6.334, –2.993)	0.000	0.0%	0.551
	Post-CMT versus pre-CMT	3	–0.173 (–1.782, 1.437)	0.833	53.1%	0.119
	Pre-HTEB versus pre-CMT	3	–0.229 (–1.876, 1.419)	0.785	0.9%	0.365
	Post-HTEB versus post-CMT	3	–5.149 (–6.807, –3.491)	0.000	79.7%	0.007
	*Random-effects model*		–5.055 (–8.964, –1.146)	0.011	79.7%	0.007
Tricuspid regurgitation area (mm^2^)						
	Post-HTEB versus pre-HTEB	2	–3.095 (–3.932, –2.258)	0.000	93.5%	0.000
	*Random-effects model*		–3.955 (–7.707, –0.204)	0.039	93.5%	0.000
	Post-CMT versus pre-CMT	2	–0.378 (–1.509, 0.753)	0.512	0.0%	0.875
	Pre-HTEB versus pre-CMT	2	–0.820 (–1.828, 0.188)	0.111	51.9%	0.149
	Post-HTEB versus post-CMT	2	–2.866 (–3.914, –1.819)	0.000	92.1%	0.000
	*Random-effects model*		–5.545 (–12.341, 1.251)	0.110	92.1%	0.000
Pulmonary arterial pressure (mmHg)						
	Post-HTEB versus pre-HTEB	2	–18.600 (–22.544, –14.656)	0.000	0.0%	0.917
	Post-CMT versus pre-CMT	2	–2.057 (–7.609, 3.495)	0.468	0.0%	0.986
	Pre-HTEB versus pre-CMT	2	–0.483 (–5.516, 4.550)	0.851	0.0%	0.685
	Post-HTEB versus post-CMT	2	–17.196 (–22.036, –12.356)	0.000	0.0%	0.580
Brain natriuretic peptide (pg/mL or ng/L)						
	Post-HTEB versus pre-HTEB	2	–961.493 (–969.022, –953.964)	0.000	98.1%	0.000
	*Random-effects model*		–706.883 (–1.2 × 10^3^, –195.421)	0.007	98.1%	0.000
	Post-CMT versus pre-CMT	2	–683.279 (–689.595, –676.964)	0.000	96.6%	0.000
	*Random-effects model*		–476.191 (–897.348, –55.035)	0.027	96.6%	0.000
	Pre-HTEB versus pre-CMT	2	68.032 (61.191, 74.873)	0.000	0.0%	0.805
	Post-HTEB versus post-CMT	2	–210.429 (–217.478, –203.380)	0.000	80.2%	0.025
	*Random-effects model*		–164.775 (–275.285, –54.265)	0.003	80.2%	0.025
Parameters		Number of studies	Pooled odds ratio (OR) and 95% confidence interval	*p*-value for OR	I-square (I^2^, %)	*p*-value for heterogeneity
Total effective rate		6	5.114 (3.189, 8.203)	0.000	0.0%	0.744
	Heart failure	3	5.601 (1.990, 15.762)	0.001	0.0%	0.570
	Angina pectoris	3	4.993 (2.936, 8.492)	0.000	0.0%	0.505
Excellence rate		5	2.828 (1.968, 4.063)	0.000	0.0%	0.977
	Heart failure	2	2.768 (0.808, 9.480)	0.105	0.0%	0.659
	Angina pectoris	3	2.833 (1.939, 4.140)	0.000	0.0%	0.896
Inefficient rate		5	0.186 (0.112, 0.307)	0.000	0.0%	0.675
	Heart failure	2	0.097 (0.020, 0.463)	0.003	0.0%	0.772
	Angina pectoris	3	0.200 (0.118, 0.341)	0.000	0.0%	0.505

A random-effects model was added alongside the fixed-effects model when high heterogeneity was observed among the included studies (italicized). HTEB, high thoracic epidural blockade; CMT, conventional medical treatment; NYHA, New York Heart Association.

### 3.6 Analysis of Case–Series Studies

A comparison was conducted between pre- and post-HTEB treatment. The mean HR and BP were reduced after adjunctive HTEB treatment. Moreover, HTEB treatment alleviated the myocardial ischemia, reflected by decreased frequency and duration of ischemic episodes, and improved ΣST-T, NST-T, QTd, and QTCd. Further, HTEB regulated the autonomic neural function in ischemic cardiomyopathy by increasing SDNN and SDANN. In contrast, HTEB lowered the NYHA cardiac functional classification, prolonged the 6-minute walk distance, enhanced cardiac output, limited cardiac expansion, and reduced the cardiothoracic ratio. HTEB affects plasma neurohormones by decreasing the NT-proBNP, angiotensin-converting enzyme 2, and norepinephrine levels. Hemodynamic indicators improved after adjunctive HTEB treatment, as reflected in the enhanced mPAP, pulmonary capillary wedge pressure (PCWP), PVRI, SVI, and SVRI. Furthermore, the summary effective rate of adjunctive HTEB treatment was 96.8%, and the excellence and inefficiency rates were 67.7% and 3.3%, respectively. We applied a random-effects model to reanalyze these results, particularly for studies with an I^2^ greater than 75%. Consistent results were observed from the random-effects model, excluding the indicators, cardiac output (*p* for effect >0.05), mean pulmonary arterial pressure (*p* for effect >0.05), pulmonary capillary wedge (*p* for effect >0.05), and angiotensin-converting enzyme 2 (*p* for effect >0.05). The detailed results are presented in Table 2.

**Table 2.** **Pooled change in cardiac indicators post-adjunctive HTEB treatment compared to pretreatment**.

Parameters	Number of studies	Pooled weighted mean difference (WMD) and 95% confidence interval	*p*-value for WMD	I-squared (I^2^, %)	*p*-value for heterogeneity
Frequency of ischemic episodes (24 hours)	3	–4.230 (–4.758, –3.703)	0.000	27.3%	0.253
Duration of ischemic episodes (minutes)	3	–8.660 (–9.991, –7.328)	0.000	37.7%	0.201
Mean heart rate (times per min)	14	–5.210 (–6.046, –4.374)	0.000	59.4%	0.001
Systolic blood pressure (mmHg)	8	–8.260 (–10.010, –6.509)	0.000	87.3%	0.000
*Random-effects model*		–10.703 (–15.951, –5.456)	0.000	87.3%	0.000
Diastolic blood pressure (mmHg)	8	–8.015 (–8.705, –7.324)	0.000	99.5%	0.000
*Random-effects model*		–12.712 (–23.362, –2.062)	0.019	99.5%	0.000
Mean arterial blood pressure (mmHg)	4	–8.895 (–12.059, –5.732)	0.000	57.1%	0.072
ΣST-T (mv)	7	–1.192 (–1.332, –1.053)	0.000	0.0%	0.613
NST-T	6	–1.614 (–1.828, –1.401)	0.000	0.0%	0.862
Q-T dispersion	3	–9.313 (–14.524, –4.103)	0.000	0.0%	0.983
Q-T corrected dispersion	3	–10.287 (–17.283, –3.290)	0.004	31.0%	0.235
J-TD	2	–9.045 (–15.789, –2.300)	0.009	0.0%	0.623
SDNN (ms)	2	20.455 (11.077, 29.833)	0.000	73.2%	0.053
SDANN (ms)	2	21.425 (13.472, 29.378)	0.000	63.2%	0.099
NYHA cardiac functional classification	15	–1.650 (–1.734, –1.566)	0.000	42.4%	0.024
Left ventricular ejection fraction (%)	23	9.792 (9.031, 10.554)	0.000	87.8%	0.000
*Random-effects model*		10.041 (7.724, 12.359)	0.000	87.8%	0.000
Left ventricular fraction shortening (%)	7	4.425 (3.577, 5.273)	0.000	30.9%	0.181
N-terminal pro-brain natriuretic peptide (ng/L)	8	–4.0 × 10^3^ (–4.4 × 10^3^, –3.6 × 10^3^)	0.000	95.5%	0.000
*Random-effects model*		–4.0 × 10^3^ (–5.9 × 10^3^, –2.0 × 10^3^)	0.000	95.5%	0.000
Left atrial dimension (mm)	10	–5.053 (–6.222, –3.884)	0.000	0.0%	0.623
Left ventricular end-diastolic dimension (mm)	17	–7.151 (–7.902, –6.400)	0.000	81.4%	0.000
*Random-effects model*		–5.291 (–7.192, –3.391)	0.000	81.4%	0.000
Left ventricular end-systolic volume (mL)	7	–24.258 (–34.572, –13.944)	0.000	43.7%	0.087
Left ventricular end-diastolic volume (mL)	7	–13.649 (–26.893, –0.404)	0.043	41.9%	0.099
E peaking/A peaking ratio	3	0.281 (0.129, 0.432)	0.000	0.0%	0.538
A peaking/E peaking ratio	2	–0.670 (–0.815, –0.524)	0.000	0.0%	0.640
Cardiac output (L/min)	4	0.823 (0.501, 1.146)	0.000	84.2%	0.000
*Random-effects model*		0.803 (–0.029, 1.636)	0.059	84.2%	0.000
Stroke volume (mL)	5	9.469 (5.558, 13.380)	0.000	30.6%	0.206
Cardiothoracic ratio (CTR)	3	–0.128 (–0.137, –0.119)	0.000	46.9%	0.152
6-minute walk distance (6MWT)	3	132.564 (75.010, 190.117)	0.000	0.0%	0.444
Cardiac index (L min^–⁢1^ m^–⁢2^)	3	0.446 (0.284, 0.608)	0.000	80.5%	0.006
*Random-effects model*		0.423 (0.044, 0.802)	0.029	80.5%	0.006
Mean pulmonary arterial pressure (mmHg)	2	–3.189 (–4.309, –2.068)	0.000	93.0%	0.000
*Random-effects model*		–2.827 (–7.139, 1.484)	0.199	93.0%	0.000
Pulmonary capillary wedge pressure (PCWP, mmHg)	2	–2.826 (–3.857, –1.796)	0.000	94.6%	0.000
*Random-effects model*		–2.641 (–7.090, 1.808)	0.245	94.6%	0.000
Pulmonary vascular resistance indices (PVRI, mmHg min L^–⁢1^ m^2^)	2	–26.803 (–41.816, –11.789)	0.000	4.0%	0.308
Stroke volume index (SVI, mL/m^2^)	2	5.110 (2.010, 8.210)	0.001	0.0%	0.493
Systemic vascular resistance indices (SVRI, mmHg min L^–⁢1^ m^2^)	3	–182.480 (–270.393, –94.567)	0.000	73.1%	0.024
Angiotensin-converting enzyme 2	2	–20.508 (–24.087, –16.928)	0.000	95.9%	0.000
*Random-effects model*		–37.543 (–76.590, 1.503)	0.059	95.9%	0.000
Norepinephrine	2	–228.264 (–264.134, –192.394)	0.000	71.7%	0.029
Parameters	Number of studies	Pooled ES and 95% confidence interval	*p*-value for ES	I-squared (I^2^, %)	*p*-value for heterogeneity
Total effective rate	5	0.968 (0.944, 0.991)	0.000	0.0%	0.573
Excellence rate	8	0.677 (0.595, 0.76)	0.000	57.9%	0.011
Inefficient rate	5	0.033 (0.009, 0.057)	0.007	0.0%	0.572

A random-effects model was added alongside the fixed-effects model when high heterogeneity was observed among the included studies (italicized). HTEB, high thoracic epidural blockade; NYHA, New York Heart Association; SDNN, standard deviation of NN intervals; SDANN, standard deviation of the average NN intervals; ES, effect size.

### 3.7 Merged Analysis of HTEB Groups From Case–Control Studies and Case–Series Studies

The HTEB groups in the 25 case–control studies and 38 case–series were merged, and synthetic single-rate meta-analyses were performed to estimate the ESs of the short-term outcomes post-adjunctive HTEB. The results showed that HTEB reduces HR, BPs, and NYHA cardiac functional classification. Moreover, HTEB treatment was associated with improved left ventricular output and cardiac remodeling depicted by enhanced LVEF, LVFS, LAD, LVEDD, LVESV, LVEDV, and CI. The summary effective rate of adjunctive HTEB treatment was 95.2%, and the excellent and inefficient rates were 56.9% and 4.9%, respectively. We applied a random-effects model to reanalyze these results, particularly for studies with an I^2^ greater than 75%. Consistent results were observed from the random-effects model. The results are listed in Table 3.

**Table 3.** **Pooled changes in cardiac indicators post-adjunctive HTEB treatment compared to pretreatment**.

Parameters	Number of studies	Pooled weighted mean difference (WMD) and 95% confidence interval	*p*-value for WMD	I-squared (I^2^, %)	*p*-value for heterogeneity
Heart rate (min)	21	–8.093 (–8.806, –7.381)	0.000	91.6%	0.000
*Random-effects model*		–8.093 (–8.806, –7.381)	0.000	91.6%	0.000
Systolic blood pressure (mmHg)	13	–9.057 (–10.067, –8.047)	0.000	96.6%	0.000
*Random-effects model*		–13.072 (–18.857, –7.288)	0.000	96.6%	0.000
Diastolic blood pressure (mmHg)	12	–8.574 (–9.236, –7.913)	0.000	99.3%	0.000
*Random-effects model*		–12.082 (–20.495, –3.670)	0.005	99.3%	0.000
Mean blood pressure (mmHg)	6	–9.341 (–11.765, –6.917)	0.000	66.1%	0.011
NYHA cardiac functional classification	24	–1.526 (–1.586, –1.466)	0.000	72.8%	0.000
Left ventricular ejection fraction (%)	39	9.651 (9.082, 10.220)	0.000	85.1%	0.000
*Random-effects model*		9.970 (8.387, 11.553)	0.000	85.1%	0.000
Left ventricular fraction shortening (%)	15	4.365 (3.840, 4.890)	0.000	53.8%	0.006
Left atrial dimension (mm)	18	–4.748 (–5.567, –3.929)	0.000	0.0%	0.757
Left ventricular end diastolic dimension (mm)	33	–6.356 (–6.918, –5.793)	0.000	74.3%	0.000
Left ventricular end-systolic volume (mL)	10	–29.562 (–38.200, –20.924)	0.000	50.9%	0.026
Left ventricular end-diastolic volume (mL)	10	–20.439 (–31.559, –9.319)	0.000	43.8%	0.058
Cardiac index (L min^–⁢1^ m^–⁢2^)	5	0.546 (0.469, 0.623)	0.000	69.1%	0.011
Parameters	Number of studies	Pooled ES and 95% confidence interval	*p*-value for ES	I-squared (I^2^, %)	*p*-value for heterogeneity
Total effective rate	11	0.952 (0.934, 0.970)	0.007	0.0%	0.509
Excellence rate (*random-effects model*)	14	0.569 (0.460, 0.679)	0.000	89.7%	0.000
Excellence rate *(fixed-effect model)*	14	0.564 (0.530, 0.597)	0.000	89.7%	0.000
Inefficient rate	10	0.049 (0.031, 0.067)	0.000	0.0%	0.521

HTEB, high thoracic epidural blockade; ES, effect size; NYHA, New York Heart Association.

### 3.8 Meta-regression and Subgroup Analysis in Case–Control Studies

Multivariable meta-regression analysis was conducted on the NYHA cardiac functional classification and LVEF based on the variables, including HF etiology, anesthetic agent doses, HTEB intervention frequency, and HTEB intervention duration. Two indicators exhibited low fitting degrees, with adjusted R^2^ values of 11.81% for NYHA and 19.28% for LVEF, as identified through multivariable meta-regression analysis. No statistical significance was observed. Therefore, independent variable regression was performed. The results showed that the inconsistency of LVEF in individual studies was related to the HF etiology (**Supplementary Table 12**). The results of the subgroup analysis noted a slight inconsistency in the subgroups, such as dilated cardiomyopathy (DCM) (I^2^ = 33.3%) and ischemic cardiomyopathy (ICM) (I^2^ = 0%), and a slight to moderate inconsistency in the multiple cardiovascular diseases (I^2^ = 47.9%) (**Supplementary Table 13**).

### 3.9 Meta-regression and Subgroup Analysis of Case–Series Studies

A meta-regression analysis was conducted for the case–series studies to explore the inconsistency between individual studies for NT-pro BNP, LVEF, and LVEDD. No positive covariate was identified in the multivariate regression analysis. The results of the univariate regression analyses implicated the identified covariates, such as the dose of the anesthetic agents (*p* = 0.037), in relation to the LVEDD inconsistency between individual studies. Thus, subgroup analyses were performed on the three indicators. The NT-pro BNP analysis observed a wide distribution in HF etiologies. Therefore, three covariates were estimated based on the anesthetic agent doses, HTEB intervention frequency, and HTEB intervention duration. Good homogeneities were identified between the three studies [74, 75, 76]. A high heterogeneity was observed among the included studies in the subgroup labeled coronary artery disease of LVEF (I^2^ = 96.1%; seven reports). Moderate heterogeneity was noted in the subgroup labeled dilated cardiomyopathy for LVEF (I^2^ = 49.4%; 10 reports), while slight heterogeneity was found for LVEF in the subset categorized as other cardiovascular diseases (I^2^ = 28.3%; eight reports). Homogeneities were observed in the subgroups of the HTEB intervention duration, including 4–8 weeks (four reports), 2 weeks (two reports), 4–6 weeks (three reports), and minutes (two reports). Meanwhile, substantial heterogeneity (I^2^ = 81.4) was observed among involved studies for the LVEDD estimation (17 reports). Based on the classification of “dose of anesthetic agents”, the results of the meta-regression and subgroup analyses represented good consistency in the subgroup labeled as “0.5% lidocaine (3–5 mL)” (I^2^ = 9.8%; seven reports), “0.5% lidocaine (5 mL) or 0.2% ropivacaine (5 mL)” (I^2^ = 0%; three reports), and “0.5% lidocaine (5 mL)” (I^2^ = 0%; four reports). High inconsistency was observed in the subgroup labeled “others” (I^2^ = 83.1; 3 reports). These results are presented in **Supplementary Tables 14** and **15**.

### 3.10 Meta-regression and Subgroup Analysis in Merged Studies

Meta-regression analyses were conducted for NYHA, LVEF, and LVEDD between merged studies, with a positive result observed in the LVEF analysis. Covariates, such as the HTEB intervention duration (multivariate regression, *p* = 0.011; univariate regression, *p* = 0.008), were identified as related to the inconsistency between studies. Subgroup analyses were performed on the LVEF based on the covariates and those in the meta-regression analyses. Consistency between reports was identified in the subgroups, such as dilated cardiomyopathy (I^2^ = 28.8%; 18 reports; *p*-value for WMD = 0.000) and other cardiomyopathies (I^2^ = 27.3%; 11 reports; *p*-value for WMD = 0.000). The HTEB intervention duration subgroups, including minutes (I^2^ = 25.1%; two reports), 1 week (I^2^ = 22.1%; two reports), 2–3 weeks (I^2^ = 0.0%; three reports), 4–6 weeks (I^2^ = 0.0%; three reports), and 4–8 weeks (I^2^ = 0.0%; five reports) showed good homogeneity that potentially contributed to the inconsistency in the pooled effects of LVEF between reports. These results are presented in **Supplementary Tables 16** and **17**.

## 4. Discussion 

Multiple earlier meta-analyses have demonstrated that HTEB improves outcomes in patients undergoing both cardiac and non-cardiac surgeries. The use of epidural anesthesia during surgical procedures provides additional benefits, including reducing supraventricular arrhythmias, alleviating respiratory issues, and reducing major adverse cardiac events and mortality [82, 83, 84]. Nevertheless, questions and controversies remain regarding the anti-arrhythmic effects of HTEB. Several studies and a meta-analysis have suggested that thoracic epidural anesthesia shows no beneficial efficacy in preventing postoperative AF in patients undergoing cardiac surgery [85, 86]. However, two uncontrolled studies with small sample sizes suggested that patients with ventricular arrhythmias may benefit from HTEB treatment [87, 88]. No conclusive evidence exists that supports the use of bilateral cardiac sympathetic denervation for antiarrhythmic purposes in patients with cardiomyopathies; meanwhile, heart transplantation represents the most effective treatment among patients with end-stage cardiomyopathy and arrhythmias [89]. HTEB may be beneficial for ameliorating the hemodynamic abnormalities and cardiac dysfunction in cases of IHD and HF.

As the short-term effects of HTEB on cardiac performance in non-surgical patients with IHD and/or HF remain uncertain, we conducted a pooled analysis using a meta-analytic approach. The analytical procedure involved three continuous stages. Stage 1: Analysis of 25 case–control studies comparing cardiac indicators between adjunctive HTEB treatment and CMT. Stage 2: Analysis of 38 case–series studies comparing the cardiac indicators between pre- and post-HTEB treatment. Stage 3: Aggregation of the HTEB groups isolated from the case–control and case–series studies comparing the cardiac indicators between pre- and post-HTEB treatment. Most of the studies were published in Chinese because reports regarding adjunctive HTEB treatments in non-surgical patients with IHD and HF are limited in the literature published in English and other languages. The cumulative results provided valuable insights indicating that HTEB, combined with conventional medical treatments, offered additional benefits for improving cardiac function, alleviating angina caused by myocardial ischemia, and reducing cardiac dilation. Substantial heterogeneity was observed between individual studies. We addressed a part of this heterogeneity through meta-regression followed by subgroup analyses.

In the evaluation of 25 case–control studies, obvious inconsistency was observed in the cardiac functional classification of HR, SBP, DBP, MBP, and NYHA in the individual studies. In contrast to imaging examinations with relative quantitative detections, measuring the time points of HR and BP with variability are inconsistently distributed; meanwhile, the NYHA cardiac functional classification is based solely on clinical judgment. Therefore, we did not conduct a further meta-regression or subgroup analysis. Two or three studies were included in estimating the LVESV, mitral regurgitation area, tricuspid regurgitation area, RATD, RVTD, and serum BNP concentrations after HTEB treatment, and further subgroup analysis and meta-regression were unavailable due to limitations in computational efficiency, as well as in the number of reports. HTEB treatment increased LVEF with moderate heterogeneity (n = 16; I^2^ = 62.2%) among these selected studies. We further explored the potential origin of heterogeneity by using a meta-regression analysis followed by a subgroup analysis. The variables were classified as (1) HF etiology, (2) anesthetic agent doses, (3) HTEB treatment frequency, and (4) HTEB treatment duration. We found that the inconsistency between individual studies was related to HF etiology. The following subgroup analysis further indicated that HF due to DCM or ICM showed preferable homogeneity. Furthermore, in comparing the effective rate between the adjunctive HTEB group and the CMT group, the definition of effective rate in the three studies was the recovery of HF and angina pectoris due to CAD. The HTEB is more effective than CMT in treating HF and angina pectoris.

The sympathetic efferent nerve mediates angina in patients with ischemic coronary artery disease. Blocking the thoracic sympathetic segments through local anesthesia provides pain relief in the coronary ischemia syndrome and is sometimes used to manage intractable angina [90]. Olausson *et al*. [14] confirmed that continuous HTEB has superior anti-ischemic and anti-anginal effects compared to conventional therapy in patients with refractory unstable angina. Moreover, HTEB treatment can normalize the myocardial blood flow response to sympathetic stimuli, improve myocardial pH and metabolism during ischemia, increase myocardial oxygen availability, and preserve the cardiac function of the ischemic heart [13, 45, 46, 47, 48, 69, 71, 91, 92, 93, 94]. Our pooled analysis of the case–series studies indicates that HTEB exerts significant anti-ischemic effects, as evidenced by decreased frequency and duration of ischemic episodes, mean HR, and BP in patients with unstable angina pectoris. Heterogeneity was observed in the HR and BP calculations. We did not conduct a subgroup analysis due to the measured variability and randomness. In contrast, the ΣST-T, NST-T, Q-Td, and Q-Tcd parameters significantly improved after HTEB and showed good homogeneity. These electrocardiographic parameters are more objective in reflecting the improvement in myocardial ischemia.

HF is the end stage in various cardiovascular diseases, characterized by poor cardiac output and ventricular enlargement. Furthermore, HF is often associated with malignant arrhythmias and sudden cardiac arrest. DCM and ICM are common etiologies of HF. For non-cardiac surgery, HTEB has been suggested as the optimal anesthetic management for patients with DCM and HF, since HTEB can provide rapid analgesia and reduce the risk of perioperative adverse cardiac events [84]. There are limited reports on applying adjunctive HTEB in non-surgical patients with DCM. The 2011 study by Chi and colleagues [16] confirmed that HTEB treatment significantly improved cardiac function and benefited outcomes. Therefore, the observed improvement in cardiac ultrasound parameters in this study aligns with findings from other studies [14, 15]. Nevertheless, this study did not represent limited outcome information, such as detailed follow-up data. The middle and long-term benefits of HTEB on hard endpoints cannot be definitively confirmed for HF in humans. Therefore, well-designed trials with complete follow-up and adequate evidence are needed in the future [16]. Li and Liu [42] found that HTEB decreased plasma norepinephrine, cyclic adenosine monophosphate (cAMP), and cyclic guanosine monophosphatec (cGMP) levels, which was associated with improved left ventricular systolic function in patients with HF. The authors concluded that HTEB intervention improved cardiac function via blocking the NE–β-receptor–cAMP cascade and reducing cGMP content. cAMP and cGMP were found to correlate negatively with cardiac function. Nevertheless, this clinical study did not explore the underlying mechanism involved in the HTEB effect, and the decrease in these two indicators was insufficient to demonstrate the impact of HTEB on improving cardiac function by inhibiting cAMP and cGMP [16]. cAMP is a highly regulated second messenger critically involved in many intracellular processes. Serum cAMP and cGMP levels may not accurately reflect the intracellular level change [95]. Meanwhile, cAMP plays a beneficial role in preventing restenosis after following peripheral angioplasty. Hormonal stimulation inactivates the alpha subunit in the trimeric G-protein complex (Gαs), which activates adenylate cyclase. This activation leads to an increase in intracellular cAMP, subsequently activating the cAMP–protein kinase A (PKA) signaling pathway. This pathway suppresses the Ras–Raf–MAPK pathway associated with vascular smooth muscle cell (VSMC) proliferation. These effects contribute to decreased restenosis [96]. cAMP and cGMP upregulation in the failing heart may play a role in the adaptive response to compensate for reduced signaling transduction of cAMP and cGMP. Notably, HTEB reduced the serum levels of these two messengers, which may reflect the improved cardiac function via adrenergic signaling transduction, providing feedback regulation. Additionally, HTEB was associated with reduced plasma carboxy-terminal propeptide of procollagen type I (PICP) and amino-terminal propeptide of procollagen type III (PIIINP) levels [44]. Ma and colleagues [81] found that HTEB treatment helped improve cardiac function and alleviate cardiac remodeling in patients with DCM and HF based on cardiac magnetic resonance (CMR) using the late gadolinium enhancement (LGE) technique. Nevertheless, this study is a preliminary exploration that was significantly restricted by the small sample size (only eight patients) with no control arm, who were treated with HTBE for 4 weeks. The results of Ma *et al*. [81] are also pending further confirmation. The pooled results of the case–control studies demonstrated improved cardiac systolic dysfunction and cardiomegaly. However, heterogeneity was observed in the pooled result of LVEF across the 16 case–control studies. The results of the subgroup analysis, including dilated cardiomyopathy and ischemic cardiomyopathy subgroups, show favorable homogeneity. Adjunctive HTEB treatment is more effective than CTM alone, especially in treating DCM and ICM.

In addition to the pooled results from the case–control studies, we included additional case–series studies. The HTEB groups in the case–control studies were extracted and integrated with the case–series studies before the aggregated analysis was conducted. The pooled results indicated a significant improvement in cardiac function and a reduction in cardiomegaly following the adjunctive HTEB intervention. There was a wide distribution of etiologies, anesthetic agents, dosages, and HTEB intervention frequencies and durations. The disease classification was significantly inconsistent. Therefore, we did not discuss the origin of heterogeneity for all of the cardiac indicators in the case–series studies and aggregated studies further. Since we only performed a single-rate analysis using the uncontrolled case–series studies, the pooled effects we obtained failed to avoid the generation of heterogeneity. In addition, most of these case–series studies had small sample sizes that may contribute to the heterogeneity. Some evaluated indicators involved few reports (less than or equal to 3), restricted by computational efficiency, and the meta-regression or subgroup analysis was unavailable. These differences may affect the estimation of outcomes between individual studies. Nonetheless, merged effects may provide the average impacts of adjunctive HTEB treatment on cardiac function and structure in patients with IHD and HF.

Evidence showed that patients had received adequate drug intervention in these early studies. For example, patients with IHD received the combination of CMT that involved β-receptor blockers, calcium antagonists, nitrates, antiplatelet reagents, and anti-anticoagulants (aspirin and heparin). Hypolipemic therapy (statins) was not mentioned in these studies. In the treatment of HF, patients were administered the combination of CMT that included strengthening cardiac muscle contractions (digitalis and dopamine), diuresis, reducing pre- and post-cardiac overload (sodium nitroprusside), and neurohumoral regulation (β-receptor blockers, ACE-Is/ARBs, and MRA). Nevertheless, ARNI and SGLT2i were not involved in these earlier studies. HTEB treatment has proven effective compared to earlier medical regimens; however, comparisons with the new regimen outlined in the 2021 European Society of Cardiology (ESC) guidelines and the clinical benefits of incorporating HTEB therapy into this new regimen warrant further investigation. However, HTEB may be a potential therapeutic method for patients in whom medical treatment has already been optimized or for patients in whom some drugs cannot be administered due to comorbidities.

Two patterns of epidural administration were noted in the included studies. In the study conducted by Olausson *et al*. [14], an epidural bolus dose of 20 to 30 mg of bupivacaine (5 mg/mL) induced a blockage in the cardiac sympathetic segments (Th_1-5_), and continuous epidural infusion of bupivacaine was then started for at least 48 hours. In this study, HTEB resulted in a significant alleviation of refractory unstable angina. In the studies conducted by Blomberg and coworkers, pain management using HTEB began with a bolus epidural injection of 4.3 ± 0.2 mL of bupivacaine (5 mg/mL), which induced a sympathetic blockade from Th_1-8_ [45]. HTEB treatment favorably alters the oxygen supply/demand ratio within ischemic myocardial areas during ischemic chest pain. In another study by the same team, in 28 patients with unstable angina pectoris, following an initial test dose of 2 mL, subsequent incremental doses of 2 mL of the local anesthetic were administered approximately every 10 minutes to achieve a blockage encompassing at least the cardiac sympathetic segments (Th_1-5_) [46]. The HTEB treatment lasted for 6.0 ± 1.1 days. HTEB demonstrated efficacy in alleviating pain and stabilizing patients experiencing unstable angina pectoris that was resistant to conventional medical therapies [46]. In the study by Kock *et al*. [47], following an initial test dose of 2 mL, subsequent incremental doses of bupivacaine (5 mg/mL) ranging from 1 to 2 mL were administered every 10 minutes to achieve a blockade of at least the cardiac sympathetic segments Th_1-5_. In this study, HTEB improved both global and regional wall motion abnormalities in the left ventricle induced by ischemia during physical stress, and correlated with a reduction in the severity of ST-segment depression. Gramling-Babb *et al*. [48] treated 10 patients with refractory angina using HTEB. Patients received a bupivacaine bolus (0.25% to 0.5%), which was then maintained as a continuous infusion or an intermittent rebolus. HTEB produced symptomatic relief of angina pectoris. Other studies have also employed short-interval or continuous injection methods in managing angina pectoris [23, 34, 36, 55]. Although the research span is relatively lengthy, these studies [13, 14, 45, 46, 47, 48, 69, 71] exhibited a comprehensive and rigorous design for HTEB with more thorough technical information. Nevertheless, these studies used an intermittent injection pattern in the HF treatment; the bolus injection frequency was 2 to 4 hours. Different methods for performing HTBE were observed among the studies treating angina and HF. These administration patterns may potentially impact the responses of the patients to HTEB treatment. The HTEB for HF treatment times in these reports and the duration of catheter retention were longer than the treatment of angina pectoris by HTEB.

Several potential limitations in this study should be considered. First, due to the limited number of studies on this topic published in English and other languages, the current analysis primarily focuses on articles published in Chinese. Consequently, a potential publication bias may exist. Second, the inclusion period spans a long duration (from 1989 to 2023), during which the accuracy of diagnoses, treatments, and other factors may have influenced how these patients respond to HTEB treatment. This variability contributes to the heterogeneity of the included studies. The scheme of the traditional medical treatment used in the early studies for HF did not strictly adhere to the new therapeutic regimen recommended by the 2021 ESC guidelines for diagnosing and treating acute and chronic HF with reduced ejection fraction. Moreover, the medical regimen in some studies did not involve angiotensin-converting enzyme inhibitors/angiotensin receptor blockers, beta-blockers, mineralocorticoid receptor antagonists, and sodium-glucose co-transporter 2 inhibitors [2, 4]. Therefore, the clinical efficacy of HTEB in combination with the new drug regimen could not be assessed. Third, some of the earlier clinical study designs were inadequate, which may have reduced the reliability of the results. In the included case–series studies, we used a self-controlled design to compare changes in primary indicators before and after HTEB treatment. Therefore, this assessment may introduce significant bias due to the intrinsic characteristics of the included case–series studies. Lastly, we only detected publication bias based on the effects of LVEF due to the limited number of other indicators in the individual analyses. Some methods (e.g., funnel plots or regression) are only sensitive to the relationship between sample size and effect size. However, if the populations or protocols differ among the trials, legitimate heterogeneity should be reflected in the ES [97].

## 5. Conclusions

Even though the current results are limited and the application of HTEB in non-surgical patients with HF has not reached widespread consensus, our pooled results suggest that adjunctive HTEB treatment can improve cardiac performance and compensatory cardiac dilation. HTEB roughly increases the average LVEF by 10% in patients with IHD and HF, especially for DCM and ICM. A frequently-used protocol of HTEB treatment is 3–5 mL of 0.5% lidocaine hydrochloride administered intermittently via epidural infusion every 2/24 hours for 4 weeks. Adjunctive HTEB treatment may be a promising strategy for short-term cardiovascular rehabilitation; however, well-designed, larger-sample, multi-center clinical trials are needed in the future.

## Data Availability

The raw data used in our study are available from the corresponding author on reasonable request.

## Abbreviations

HTEB, High thoracic epidural blockade; IHD, ischemic heart disease; CMT, conventional medical treatment; HF, heart failure; MRAs, mineralocorticoid receptor antagonists; sGC, soluble guanylate cyclase; RAS, renin-angiotensin system; rEF, reduced ejection fraction; CAD, coronary artery disease; MeSH, Medical Subject Headings; GA, general anesthesia.

## Supplementary Material

Supplementary material associated with this article can be found, in the online version, at https://doi.org/10.31083/RCM37886.
