# Supplementary material for: Short-term Effects of High Thoracic Epidural Blockade in Patients With Ischemic Heart Disease and Heart Failure: A Systematic Review and Data Synthesis
Source: Rev Cardiovasc Med. 2025 Jul 31;26(7):37886. doi: 10.31083/RCM37886 (PMC12326434; doi:10.31083/RCM37886)
Supplement: Supplementary file 1 [file 2153-8174-26-7-37886-s1.zip › Figure caption and figure notes for Supplementary Fig 1.docx]

**Supplementary Fig. 1. Sensitivity analysis and the estimation of publication bias based on the effect of LVEF and LVEDD.** (A) Egger's publication bias plot is based on the estimation of LVEDD in the case–control trials. (B) Sensitivity analysis of LVEF in the case–series studies. (C) Sensitive analysis of LVEF after omitting one study (Chi et al. [15], 2011) in the case–series studies. (D) Egger's publication bias plot based on the estimation of LVEF after omitting one study in the case–series studies. (E) Sensitivity analysis and Egger's publication bias plots based on LVEDD in the case–series studies. (F) Sensitivity analysis and Egger's publication bias plot based on LVEDD after omitting one study (Liu et al. [57], 2001) in the case–series studies.
