# Supplementary material for: Short-term Effects of High Thoracic Epidural Blockade in Patients With Ischemic Heart Disease and Heart Failure: A Systematic Review and Data Synthesis
Source: Rev Cardiovasc Med. 2025 Jul 31;26(7):37886. doi: 10.31083/RCM37886 (PMC12326434; doi:10.31083/RCM37886)
Supplement: Supplementary file 1 [file 2153-8174-26-7-37886-s1.zip › Supplemental material.docx]

Supplementary Material

Title: Short-term effect of adjunctive high thoracic epidural blockade in hospitalized patients with ischemic heart disease and heart failure: a systematic review and meta-analysis

Supplementary Table 1: Literature search strategy

Supplementary Table 2: Transformation of NYHA functional classification in case control studies

Supplementary Table 3: Transformation of NYHA functional classification in case series studies

Supplementary Table 4: Clinical characteristics of trials included in the pooled analysis

Supplementary Table 5: HTEB intervention

Supplementary Table 6: Methodology quality assessment of case-control trials included in the analysis using modified Jadad score

Supplementary Table 7: Estimating literature quality in individual case-control study using Newcastle-Ottawa Scale

Supplementary Table 8: Total score of Newcastle-Ottawa Scale (NOS) in individual case-control studies

Supplementary Table 9: Institute of Health Economics (IHE) items

Supplementary Table 10: IHE estimation

Supplementary Table 11: Estimation of HTEB related complications in involved studies

Supplementary Table 12: Meta-regression analysis of LVEF in case control trials

Supplementary Table 13: Subgroup analysis of LVEF based on the etiologies of heart failure in case control trials

Supplementary Table 14: Meta-regression analysis of NT-pro BNP, LVEF and LVEDD in case series studies

Supplementary Table 15: Subgroup analysis of BNP, LVEF, LVEDD in case series studies

Supplementary Table 16: Meta-regression analysis of NYHA, LVEF, LVEDD in merged studies

Supplementary Table 17: Subgroup analysis of LVEF in merged studies

Supplementary Table 1: Literature search strategy

| 1. Intervention/treatment: Thoracic Epidural blockade; Cardiac sympathetic blockade; Thoracic epidural analgesia; Epidural anesthesia; Epidural analgesia; Cardiac sympathectomy;  2. Etiology: Heart failure, Ischemic heart disease, Dilated cardiomyopathy, Angina, Coronary artery disease  3. Outcomes: Left ventricular fraction ejection, Left ventricular function |
| --- |
| **1. Pubmed (Clinical Trial)**  #1[Title/Abstract]:"Thoracic Epidural blockade" [MeSH Terms] AND "Heart failure" [MeSH Terms] 3 results; 2 articles found by citation matching |
| #2 [Title/Abstract]:" Cardiac sympathetic blockade " [MeSH Terms] AND "Heart failure" [MeSH Terms] 57 results; 7 articles found by citation matching |
| #3 [Title/Abstract]:" Thoracic epidural analgesia " [MeSH Terms] AND "Heart failure" [MeSH Terms] 4 results; 2 articles found by citation matching |
| #4 [Title/Abstract]:" Epidural anesthesia " [MeSH Terms] AND "Heart failure" [MeSH Terms] 22 results; 8 articles found by citation matching |
| #5 [Title/Abstract]:" Epidural analgesia " [MeSH Terms] AND "Heart failure" [MeSH Terms] 21 results; 2 articles found by citation matching |
| #6 [Title/Abstract]:" Cardiac sympathectomy " [MeSH Terms] AND "Heart failure" [MeSH Terms] 22 results; 1 articles found by citation matching |
| #7 [Title/Abstract]:" Thoracic Epidural blockade " [MeSH Terms] AND " Ischemic heart disease " [MeSH Terms] 4 results |
| #8[Title/Abstract]:" Cardiac sympathetic blockade " [MeSH Terms] AND " Ischemic heart disease " [MeSH Terms] 35 results |
| #9 [Title/Abstract]:" Thoracic epidural analgesia " [MeSH Terms] AND " Ischemic heart disease " [MeSH Terms] 25 results; 2 articles found by citation matching |
| #10[Title/Abstract]:" Epidural anesthesia " [MeSH Terms] AND " Ischemic heart disease " [MeSH Terms] 67 results; 4 articles found by citation matching |
| #11 [Title/Abstract]:" Epidural analgesia " [MeSH Terms] AND " Ischemic heart disease " [MeSH Terms] 40 results; 2 articles found by citation matching |
| #12 [Title/Abstract]:" Cardiac sympathectomy " [MeSH Terms] AND " Ischemic heart disease " [MeSH Terms] 14 results |
| #13[Title/Abstract]:" Thoracic Epidural blockade " [MeSH Terms] AND " Dilated cardiomyopathy " [MeSH Terms] 3 results |
| #14[Title/Abstract]:" Cardiac sympathetic blockade " [MeSH Terms] AND " Dilated cardiomyopathy " [MeSH Terms] 10 results; 1 article found by citation matching |
| #15 [Title/Abstract]:" Thoracic epidural analgesia " [MeSH Terms] AND " Dilated cardiomyopathy " [MeSH Terms] 0 results |
| #16 [Title/Abstract]:" Epidural anesthesia " [MeSH Terms] AND " Dilated cardiomyopathy " [MeSH Terms] 2 results; 10 article found by citation matching |
| #17 [Title/Abstract]:" Epidural analgesia " [MeSH Terms] AND " Dilated cardiomyopathy " [MeSH Terms] 2 articles found by citation matching |
| #18 [Title/Abstract]:" Cardiac sympathectomy " [MeSH Terms] AND " Dilated cardiomyopathy " [MeSH Terms] 2 articles found by citation matching |
| #19 [Title/Abstract]:" Thoracic Epidural blockade " [MeSH Terms] AND " Angina " [MeSH Terms] 2 results |
| #20 [Title/Abstract]:" Cardiac sympathetic blockade " [MeSH Terms] AND " Angina " [MeSH Terms] 9 results; 1 articles found by citation matching |
| #21 [Title/Abstract]:" Thoracic epidural analgesia " [MeSH Terms] AND " Angina " [MeSH Terms] 4 results; 4 articles found by citation matching |
| #22 [Title/Abstract]:" Epidural anesthesia " [MeSH Terms] AND " Angina " [MeSH Terms] 10 results |
| #23 [Title/Abstract]:" Epidural analgesia " [MeSH Terms] AND " Angina " [MeSH Terms] 8 results |
| #24 [Title/Abstract]:" Cardiac sympathectomy " [MeSH Terms] AND " Angina " [MeSH Terms] 2 results |
| #25 [Title/Abstract]:" Thoracic Epidural blockade " [MeSH Terms] AND " Coronary artery disease " [MeSH Terms] 2 results |
| #26 [Title/Abstract]:" Cardiac sympathetic blockade " [MeSH Terms] AND " Coronary artery disease " [MeSH Terms] 15 results |
| #27 [Title/Abstract]:" Thoracic epidural analgesia " [MeSH Terms] AND " Coronary artery disease " [MeSH Terms] 10 results; 3 articles found by citation matching |
| #28 [Title/Abstract]:" Epidural anesthesia " [MeSH Terms] AND " Coronary artery disease " [MeSH Terms] 28 results |
| #29 [Title/Abstract]:" Epidural analgesia " [MeSH Terms] AND " Coronary artery disease " [MeSH Terms] 14 results; 3 articles found by citation matching |
| #30 [Title/Abstract]:" Cardiac sympathectomy " [MeSH Terms] AND " Coronary artery disease " [MeSH Terms] 8 results |
| #31 [Title/Abstract]: (("Thoracic Epidural blockade" [MeSH Terms]) OR ("Cardiac sympathetic blockade" [MeSH Terms]) OR ("Thoracic epidural analgesia" [MeSH Terms]) OR ("Epidural anesthesia" [MeSH Terms]) OR ("Epidural analgesia" [MeSH Terms]) OR ("Cardiac sympathectomy" [MeSH Terms])) AND (("Heart failure" [MeSH Terms]) OR ("Ischemic heart disease" [MeSH Terms]) OR ("Dilated cardiomyopathy" [MeSH Terms]) OR ("Angina" [MeSH Terms]) OR ("Coronary artery disease" [MeSH Terms])) AND (("Left ventricular fraction ejection" [MeSH Terms]) OR ("Left ventricular function" [MeSH Terms])) 45 results |
| **2. Web of Science (MeSH words and Assembles)** |
| Thoracic Epidural blockade AND Heart failure OR Cardiac sympathetic blockade AND Heart failure OR Thoracic epidural analgesia AND Heart failure OR Epidural anesthesia AND Heart failure OR Epidural analgesia AND Heart failure OR Cardiac sympathectomy AND Heart failure OR Thoracic Epidural blockade AND Ischemic heart disease OR Cardiac sympathetic blockade AND Ischemic heart disease OR Thoracic epidural analgesia AND Ischemic heart disease OR Epidural anesthesia AND Ischemic heart disease OR Epidural analgesia AND Ischemic heart disease OR Cardiac sympathectomy AND Ischemic heart disease OR Thoracic Epidural blockade AND Dilated cardiomyopathy OR Cardiac sympathetic blockade AND Dilated cardiomyopathy OR Thoracic epidural analgesia AND Dilated cardiomyopathy OR Epidural anesthesia AND Dilated cardiomyopathy OR Epidural analgesia AND Dilated cardiomyopathy OR Cardiac sympathectomy AND Dilated cardiomyopathy OR Thoracic Epidural blockade AND Angina OR Cardiac sympathetic blockade AND Angina OR Thoracic epidural analgesia AND Angina OR Epidural anesthesia AND Angina OR Epidural analgesia AND Angina OR Cardiac sympathectomy AND Angina OR Thoracic Epidural blockade AND Coronary artery disease OR Cardiac sympathetic blockade AND Coronary artery disease OR Thoracic epidural analgesia AND Coronary artery disease OR Epidural anesthesia AND Coronary artery disease OR Epidural analgesia AND Coronary artery disease OR Cardiac sympathectomy Coronary artery disease OR Thoracic Epidural blockade AND Left ventricular fraction ejection OR Cardiac sympathetic blockade AND Left ventricular fraction ejection OR Thoracic epidural analgesia AND Left ventricular fraction ejection OR Epidural anesthesia AND Left ventricular fraction ejection OR Epidural analgesia AND Left ventricular fraction ejection OR Cardiac sympathectomy Left ventricular fraction ejection OR Thoracic Epidural blockade AND Left ventricular function OR Cardiac sympathetic blockade AND Left ventricular function OR Thoracic epidural analgesia AND Left ventricular function OR Epidural anesthesia AND Left ventricular function OR Epidural analgesia AND Left ventricular function OR Cardiac sympathectomy AND Left ventricular function |
| English 2,567; Japanese 114; German 65; Chinese 50; French 41  Article 2,476; Other 1,030; Review Article 524; Case Report 330; Abstract 304; Meeting 298; Clinical Trial 273; Awarded Grant 159; Editorial Material 59; Letter 51; Dissertation Thesis 34; Patent 14; Reference Material 11; Book 8; Retracted Publication 7; Early Access 5; Unspecified 4; Correction 1; Publication With Expression Of Concern 1; Retraction 1 |
| **3. Emb@se (MeSH words and Assembles) (clinical trial)** |
| #1 ('thoracic epidural blockade' OR (thoracic AND epidural AND blockade)) AND ('heart failure'/exp OR 'heart failure' OR (('heart'/exp OR heart) AND ('failure'/exp OR failure))) 6 results |
| #2 ('cardiac sympathetic blockade' OR (('cardiac'/exp OR cardiac) AND ('sympathetic'/exp OR sympathetic) AND blockade)) AND ('heart failure'/exp OR 'heart failure' OR (('heart'/exp OR heart) AND ('failure'/exp OR failure))) 77 results |
| #3 ('thoracic epidural analgesia'/exp OR 'thoracic epidural analgesia' OR (thoracic AND epidural AND ('analgesia'/exp OR analgesia))) AND ('heart failure'/exp OR 'heart failure' OR (('heart'/exp OR heart) AND ('failure'/exp OR failure))) 20 results |
| #4 ('epidural anesthesia'/exp OR 'epidural anesthesia' OR (epidural AND ('anesthesia'/exp OR anesthesia))) AND ('heart failure'/exp OR 'heart failure' OR (('heart'/exp OR heart) AND ('failure'/exp OR failure))) 132 results |
| #5 ('epidural analgesia'/exp OR 'epidural analgesia' OR (epidural AND ('analgesia'/exp OR analgesia))) AND ('heart failure'/exp OR 'heart failure' OR (('heart'/exp OR heart) AND ('failure'/exp OR failure))) 81 results |
| #6 ('cardiac sympathectomy' OR (('cardiac'/exp OR cardiac) AND ('sympathectomy'/exp OR sympathectomy))) AND ('heart failure'/exp OR 'heart failure' OR (('heart'/exp OR heart) AND ('failure'/exp OR failure))) 4 results |
| #7 ('thoracic epidural blockade' OR (thoracic AND epidural AND blockade)) AND ('ischemic heart disease'/exp OR 'ischemic heart disease' OR (ischemic AND ('heart'/exp OR heart) AND ('disease'/exp OR disease))) 9 results |
| #8('cardiac sympathetic blockade' OR (('cardiac'/exp OR cardiac) AND ('sympathetic'/exp OR sympathetic) AND blockade)) AND ('ischemic heart disease'/exp OR 'ischemic heart disease' OR (ischemic AND ('heart'/exp OR heart) AND ('disease'/exp OR disease))) 44 results |
| #9 ('thoracic epidural analgesia'/exp OR 'thoracic epidural analgesia' OR (thoracic AND epidural AND ('analgesia'/exp OR analgesia))) AND ('ischemic heart disease'/exp OR 'ischemic heart disease' OR (ischemic AND ('heart'/exp OR heart) AND ('disease'/exp OR disease))) 32 results |
| #10 ('epidural anesthesia'/exp OR 'epidural anesthesia' OR (epidural AND ('anesthesia'/exp OR anesthesia))) AND ('ischemic heart disease'/exp OR 'ischemic heart disease' OR (ischemic AND ('heart'/exp OR heart) AND ('disease'/exp OR disease))) 167 results |
| #11 ('epidural analgesia'/exp OR 'epidural analgesia' OR (epidural AND ('analgesia'/exp OR analgesia))) AND ('ischemic heart disease'/exp OR 'ischemic heart disease' OR (ischemic AND ('heart'/exp OR heart) AND ('disease'/exp OR disease))) 96 results |
| #12 ('cardiac sympathectomy' OR (('cardiac'/exp OR cardiac) AND ('sympathectomy'/exp OR sympathectomy))) AND ('ischemic heart disease'/exp OR 'ischemic heart disease' OR (ischemic AND ('heart'/exp OR heart) AND ('disease'/exp OR disease))) 16 results |
| #13 ('thoracic epidural blockade' OR (thoracic AND epidural AND blockade)) AND ('dilated cardiomyopathy'/exp OR 'dilated cardiomyopathy' OR (dilated AND ('cardiomyopathy'/exp OR cardiomyopathy))) 0 results |
| #14 ('cardiac sympathetic blockade' OR (('cardiac'/exp OR cardiac) AND ('sympathetic'/exp OR sympathetic) AND blockade)) AND ('dilated cardiomyopathy'/exp OR 'dilated cardiomyopathy' OR (dilated AND ('cardiomyopathy'/exp OR cardiomyopathy))) 13 results |
| #15 ('thoracic epidural analgesia'/exp OR 'thoracic epidural analgesia' OR (thoracic AND epidural AND ('analgesia'/exp OR analgesia))) AND ('dilated cardiomyopathy'/exp OR 'dilated cardiomyopathy' OR (dilated AND ('cardiomyopathy'/exp OR cardiomyopathy))) 0 results |
| #16 ('epidural anesthesia'/exp OR 'epidural anesthesia' OR (epidural AND ('anesthesia'/exp OR anesthesia))) AND ('dilated cardiomyopathy'/exp OR 'dilated cardiomyopathy' OR (dilated AND ('cardiomyopathy'/exp OR cardiomyopathy))) 4 results |
| #17 ('epidural analgesia'/exp OR 'epidural analgesia' OR (epidural AND ('analgesia'/exp OR analgesia))) AND ('dilated cardiomyopathy'/exp OR 'dilated cardiomyopathy' OR (dilated AND ('cardiomyopathy'/exp OR cardiomyopathy))) 0 results |
| #18 ('epidural analgesia'/exp OR 'epidural analgesia' OR (epidural AND ('analgesia'/exp OR analgesia))) AND ('dilated cardiomyopathy'/exp OR 'dilated cardiomyopathy' OR (dilated AND ('cardiomyopathy'/exp OR cardiomyopathy))) 0 results |
| #19 ('cardiac sympathectomy' OR (('cardiac'/exp OR cardiac) AND ('sympathectomy'/exp OR sympathectomy))) AND ('dilated cardiomyopathy'/exp OR 'dilated cardiomyopathy' OR (dilated AND ('cardiomyopathy'/exp OR cardiomyopathy))) 2 results |
| #20 ('thoracic epidural blockade' OR (thoracic AND epidural AND blockade)) AND ('angina'/exp OR angina) 3 results |
| #21 ('cardiac sympathetic blockade' OR (('cardiac'/exp OR cardiac) AND ('sympathetic'/exp OR sympathetic) AND blockade)) AND ('angina'/exp OR angina) 12 results |
| #22 ('thoracic epidural analgesia'/exp OR 'thoracic epidural analgesia' OR (thoracic AND epidural AND ('analgesia'/exp OR analgesia))) AND ('angina'/exp OR angina) 8 results |
| #23 ('epidural anesthesia'/exp OR 'epidural anesthesia' OR (epidural AND ('anesthesia'/exp OR anesthesia))) AND ('angina'/exp OR angina) 31 results |
| #24 ('epidural analgesia'/exp OR 'epidural analgesia' OR (epidural AND ('analgesia'/exp OR analgesia))) AND ('angina'/exp OR angina) 17 results |
| #25 ('cardiac sympathectomy' OR (('cardiac'/exp OR cardiac) AND ('sympathectomy'/exp OR sympathectomy))) AND ('angina'/exp OR angina) 7 results |
| #26 ('thoracic epidural blockade' OR (thoracic AND epidural AND blockade)) AND ('coronary artery disease'/exp OR 'coronary artery disease' OR (coronary AND ('artery'/exp OR artery) AND ('disease'/exp OR disease))) 8 results |
| #27 ('cardiac sympathetic blockade' OR (('cardiac'/exp OR cardiac) AND ('sympathetic'/exp OR sympathetic) AND blockade)) AND ('coronary artery disease'/exp OR 'coronary artery disease' OR (coronary AND ('artery'/exp OR artery) AND ('disease'/exp OR disease))) 13 results |
| #28 ('thoracic epidural analgesia'/exp OR 'thoracic epidural analgesia' OR (thoracic AND epidural AND ('analgesia'/exp OR analgesia))) AND ('coronary artery disease'/exp OR 'coronary artery disease' OR (coronary AND ('artery'/exp OR artery) AND ('disease'/exp OR disease))) 40 results |
| #29 ('epidural anesthesia'/exp OR 'epidural anesthesia' OR (epidural AND ('anesthesia'/exp OR anesthesia))) AND ('coronary artery disease'/exp OR 'coronary artery disease' OR (coronary AND ('artery'/exp OR artery) AND ('disease'/exp OR disease))) 134 results |
| #30 ('epidural analgesia'/exp OR 'epidural analgesia' OR (epidural AND ('analgesia'/exp OR analgesia))) AND ('coronary artery disease'/exp OR 'coronary artery disease' OR (coronary AND ('artery'/exp OR artery) AND ('disease'/exp OR disease))) 64 results |
| #31('cardiac sympathectomy coronary artery disease' OR (('cardiac'/exp OR cardiac) AND ('sympathectomy'/exp OR sympathectomy) AND coronary AND ('artery'/exp OR artery) AND ('disease'/exp OR disease)) 5 results |
| #32 ('thoracic epidural blockade' OR (thoracic AND epidural AND blockade)) AND ('left ventricular fraction ejection' OR (left AND ventricular AND fraction AND ejection)) 0 results |
| #33 ('cardiac sympathetic blockade' OR (('cardiac'/exp OR cardiac) AND ('sympathetic'/exp OR sympathetic) AND blockade)) AND ('left ventricular fraction ejection' OR (left AND ventricular AND fraction AND ejection)) 11 results |
| #34 ('thoracic epidural analgesia'/exp OR 'thoracic epidural analgesia' OR (thoracic AND epidural AND ('analgesia'/exp OR analgesia))) AND ('left ventricular fraction ejection' OR (left AND ventricular AND fraction AND ejection)) 2 results |
| #35 ('epidural anesthesia'/exp OR 'epidural anesthesia' OR (epidural AND ('anesthesia'/exp OR anesthesia))) AND ('left ventricular fraction ejection' OR (left AND ventricular AND fraction AND ejection)) 5 results |
| #36 ('epidural analgesia'/exp OR 'epidural analgesia' OR (epidural AND ('analgesia'/exp OR analgesia))) AND ('left ventricular fraction ejection' OR (left AND ventricular AND fraction AND ejection)) 2 results |
| #37 ('cardiac sympathectomy left ventricular fraction ejection' OR (('cardiac'/exp OR cardiac) AND ('sympathectomy'/exp OR sympathectomy) AND left AND ventricular AND fraction AND ejection) 4 results |
| #38 ('thoracic epidural blockade' OR (thoracic AND epidural AND blockade)) AND ('left ventricular function'/exp OR 'left ventricular function' OR (left AND ventricular AND ('function'/exp OR function))) 4 results |
| #39 ('cardiac sympathetic blockade' OR (('cardiac'/exp OR cardiac) AND ('sympathetic'/exp OR sympathetic) AND blockade)) AND ('left ventricular function'/exp OR 'left ventricular function' OR (left AND ventricular AND ('function'/exp OR function))) 29 results |
| #40 ('thoracic epidural analgesia'/exp OR 'thoracic epidural analgesia' OR (thoracic AND epidural AND ('analgesia'/exp OR analgesia))) AND ('left ventricular function'/exp OR 'left ventricular function' OR (left AND ventricular AND ('function'/exp OR function))) 6 results |
| #41 ('epidural anesthesia'/exp OR 'epidural anesthesia' OR (epidural AND ('anesthesia'/exp OR anesthesia))) AND ('left ventricular function'/exp OR 'left ventricular function' OR (left AND ventricular AND ('function'/exp OR function))) 15 results |
| #42 ('epidural analgesia'/exp OR 'epidural analgesia' OR (epidural AND ('analgesia'/exp OR analgesia))) AND ('left ventricular function'/exp OR 'left ventricular function' OR (left AND ventricular AND ('function'/exp OR function))) 8 results |
| #43 ('cardiac sympathectomy' OR (('cardiac'/exp OR cardiac) AND ('sympathectomy'/exp OR sympathectomy))) AND ('left ventricular function'/exp OR 'left ventricular function' OR (left AND ventricular AND ('function'/exp OR function))) 4 results |
| #44 (((((((((((((((((((((((((((((((((((((((('thoracic epidural blockade' OR (thoracic AND epidural AND blockade)) AND ('heart failure'/exp OR 'heart failure' OR (('heart'/exp OR heart) AND ('failure'/exp OR failure))) OR 'cardiac sympathetic blockade' OR (('cardiac'/exp OR cardiac) AND ('sympathetic'/exp OR sympathetic) AND blockade)) AND ('heart failure'/exp OR 'heart failure' OR (('heart'/exp OR heart) AND ('failure'/exp OR failure))) OR 'thoracic epidural analgesia'/exp OR 'thoracic epidural analgesia' OR (thoracic AND epidural AND ('analgesia'/exp OR analgesia))) AND ('heart failure'/exp OR 'heart failure' OR (('heart'/exp OR heart) AND ('failure'/exp OR failure))) OR 'epidural anesthesia'/exp OR 'epidural anesthesia' OR (epidural AND ('anesthesia'/exp OR anesthesia))) AND ('heart failure'/exp OR 'heart failure' OR (('heart'/exp OR heart) AND ('failure'/exp OR failure))) OR 'epidural analgesia'/exp OR 'epidural analgesia' OR (epidural AND ('analgesia'/exp OR analgesia))) AND ('heart failure'/exp OR 'heart failure' OR (('heart'/exp OR heart) AND ('failure'/exp OR failure))) OR 'cardiac sympathectomy' OR (('cardiac'/exp OR cardiac) AND ('sympathectomy'/exp OR sympathectomy))) AND ('heart failure'/exp OR 'heart failure' OR (('heart'/exp OR heart) AND ('failure'/exp OR failure))) OR 'thoracic epidural blockade' OR (thoracic AND epidural AND blockade)) AND ('ischemic heart disease'/exp OR 'ischemic heart disease' OR (ischemic AND ('heart'/exp OR heart) AND ('disease'/exp OR disease))) OR 'cardiac sympathetic blockade' OR (('cardiac'/exp OR cardiac) AND ('sympathetic'/exp OR sympathetic) AND blockade)) AND ('ischemic heart disease'/exp OR 'ischemic heart disease' OR (ischemic AND ('heart'/exp OR heart) AND ('disease'/exp OR disease))) OR 'thoracic epidural analgesia'/exp OR 'thoracic epidural analgesia' OR (thoracic AND epidural AND ('analgesia'/exp OR analgesia))) AND ('ischemic heart disease'/exp OR 'ischemic heart disease' OR (ischemic AND ('heart'/exp OR heart) AND ('disease'/exp OR disease))) OR 'epidural anesthesia'/exp OR 'epidural anesthesia' OR (epidural AND ('anesthesia'/exp OR anesthesia))) AND ('ischemic heart disease'/exp OR 'ischemic heart disease' OR (ischemic AND ('heart'/exp OR heart) AND ('disease'/exp OR disease))) OR 'epidural analgesia'/exp OR 'epidural analgesia' OR (epidural AND ('analgesia'/exp OR analgesia))) AND ('ischemic heart disease'/exp OR 'ischemic heart disease' OR (ischemic AND ('heart'/exp OR heart) AND ('disease'/exp OR disease))) OR 'cardiac sympathectomy' OR (('cardiac'/exp OR cardiac) AND ('sympathectomy'/exp OR sympathectomy))) AND ('ischemic heart disease'/exp OR 'ischemic heart disease' OR (ischemic AND ('heart'/exp OR heart) AND ('disease'/exp OR disease))) OR 'thoracic epidural blockade' OR (thoracic AND epidural AND blockade)) AND ('dilated cardiomyopathy'/exp OR 'dilated cardiomyopathy' OR (dilated AND ('cardiomyopathy'/exp OR cardiomyopathy))) OR 'cardiac sympathetic blockade' OR (('cardiac'/exp OR cardiac) AND ('sympathetic'/exp OR sympathetic) AND blockade)) AND ('dilated cardiomyopathy'/exp OR 'dilated cardiomyopathy' OR (dilated AND ('cardiomyopathy'/exp OR cardiomyopathy))) OR 'thoracic epidural analgesia'/exp OR 'thoracic epidural analgesia' OR (thoracic AND epidural AND ('analgesia'/exp OR analgesia))) AND ('dilated cardiomyopathy'/exp OR 'dilated cardiomyopathy' OR (dilated AND ('cardiomyopathy'/exp OR cardiomyopathy))) OR 'epidural anesthesia'/exp OR 'epidural anesthesia' OR (epidural AND ('anesthesia'/exp OR anesthesia))) AND ('dilated cardiomyopathy'/exp OR 'dilated cardiomyopathy' OR (dilated AND ('cardiomyopathy'/exp OR cardiomyopathy))) OR 'epidural analgesia'/exp OR 'epidural analgesia' OR (epidural AND ('analgesia'/exp OR analgesia))) AND ('dilated cardiomyopathy'/exp OR 'dilated cardiomyopathy' OR (dilated AND ('cardiomyopathy'/exp OR cardiomyopathy))) OR 'cardiac sympathectomy' OR (('cardiac'/exp OR cardiac) AND ('sympathectomy'/exp OR sympathectomy))) AND ('dilated cardiomyopathy'/exp OR 'dilated cardiomyopathy' OR (dilated AND ('cardiomyopathy'/exp OR cardiomyopathy))) OR 'thoracic epidural blockade' OR (thoracic AND epidural AND blockade)) AND ('angina'/exp OR angina) OR 'cardiac sympathetic blockade' OR (('cardiac'/exp OR cardiac) AND ('sympathetic'/exp OR sympathetic) AND blockade)) AND ('angina'/exp OR angina) OR 'thoracic epidural analgesia'/exp OR 'thoracic epidural analgesia' OR (thoracic AND epidural AND ('analgesia'/exp OR analgesia))) AND ('angina'/exp OR angina) OR 'epidural anesthesia'/exp OR 'epidural anesthesia' OR (epidural AND ('anesthesia'/exp OR anesthesia))) AND ('angina'/exp OR angina) OR 'epidural analgesia'/exp OR 'epidural analgesia' OR (epidural AND ('analgesia'/exp OR analgesia))) AND ('angina'/exp OR angina) OR 'cardiac sympathectomy' OR (('cardiac'/exp OR cardiac) AND ('sympathectomy'/exp OR sympathectomy))) AND ('angina'/exp OR angina) OR 'thoracic epidural blockade' OR (thoracic AND epidural AND blockade)) AND ('coronary artery disease'/exp OR 'coronary artery disease' OR (coronary AND ('artery'/exp OR artery) AND ('disease'/exp OR disease))) OR 'cardiac sympathetic blockade' OR (('cardiac'/exp OR cardiac) AND ('sympathetic'/exp OR sympathetic) AND blockade)) AND ('coronary artery disease'/exp OR 'coronary artery disease' OR (coronary AND ('artery'/exp OR artery) AND ('disease'/exp OR disease))) OR 'thoracic epidural analgesia'/exp OR 'thoracic epidural analgesia' OR (thoracic AND epidural AND ('analgesia'/exp OR analgesia))) AND ('coronary artery disease'/exp OR 'coronary artery disease' OR (coronary AND ('artery'/exp OR artery) AND ('disease'/exp OR disease))) OR 'epidural anesthesia'/exp OR 'epidural anesthesia' OR (epidural AND ('anesthesia'/exp OR anesthesia))) AND ('coronary artery disease'/exp OR 'coronary artery disease' OR (coronary AND ('artery'/exp OR artery) AND ('disease'/exp OR disease))) OR 'epidural analgesia'/exp OR 'epidural analgesia' OR (epidural AND ('analgesia'/exp OR analgesia))) AND ('coronary artery disease'/exp OR 'coronary artery disease' OR (coronary AND ('artery'/exp OR artery) AND ('disease'/exp OR disease))) OR 'cardiac sympathectomy coronary artery disease' OR (('cardiac'/exp OR cardiac) AND ('sympathectomy'/exp OR sympathectomy) AND coronary AND ('artery'/exp OR artery) AND ('disease'/exp OR disease)) OR 'thoracic epidural blockade' OR (thoracic AND epidural AND blockade)) AND ('left ventricular fraction ejection' OR (left AND ventricular AND fraction AND ejection)) OR 'cardiac sympathetic blockade' OR (('cardiac'/exp OR cardiac) AND ('sympathetic'/exp OR sympathetic) AND blockade)) AND ('left ventricular fraction ejection' OR (left AND ventricular AND fraction AND ejection)) OR 'thoracic epidural analgesia'/exp OR 'thoracic epidural analgesia' OR (thoracic AND epidural AND ('analgesia'/exp OR analgesia))) AND ('left ventricular fraction ejection' OR (left AND ventricular AND fraction AND ejection)) OR 'epidural anesthesia'/exp OR 'epidural anesthesia' OR (epidural AND ('anesthesia'/exp OR anesthesia))) AND ('left ventricular fraction ejection' OR (left AND ventricular AND fraction AND ejection)) OR 'epidural analgesia'/exp OR 'epidural analgesia' OR (epidural AND ('analgesia'/exp OR analgesia))) AND ('left ventricular fraction ejection' OR (left AND ventricular AND fraction AND ejection)) OR 'cardiac sympathectomy left ventricular fraction ejection' OR (('cardiac'/exp OR cardiac) AND ('sympathectomy'/exp OR sympathectomy) AND left AND ventricular AND fraction AND ejection) OR 'thoracic epidural blockade' OR (thoracic AND epidural AND blockade)) AND ('left ventricular function'/exp OR 'left ventricular function' OR (left AND ventricular AND ('function'/exp OR function))) OR 'cardiac sympathetic blockade' OR (('cardiac'/exp OR cardiac) AND ('sympathetic'/exp OR sympathetic) AND blockade)) AND ('left ventricular function'/exp OR 'left ventricular function' OR (left AND ventricular AND ('function'/exp OR function))) OR 'thoracic epidural analgesia'/exp OR 'thoracic epidural analgesia' OR (thoracic AND epidural AND ('analgesia'/exp OR analgesia))) AND ('left ventricular function'/exp OR 'left ventricular function' OR (left AND ventricular AND ('function'/exp OR function))) OR 'epidural anesthesia'/exp OR 'epidural anesthesia' OR (epidural AND ('anesthesia'/exp OR anesthesia))) AND ('left ventricular function'/exp OR 'left ventricular function' OR (left AND ventricular AND ('function'/exp OR function))) OR 'epidural analgesia'/exp OR 'epidural analgesia' OR (epidural AND ('analgesia'/exp OR analgesia))) AND ('left ventricular function'/exp OR 'left ventricular function' OR (left AND ventricular AND ('function'/exp OR function))) OR 'cardiac sympathectomy' OR (('cardiac'/exp OR cardiac) AND ('sympathectomy'/exp OR sympathectomy))) AND ('left ventricular function'/exp OR 'left ventricular function' OR (left AND ventricular AND ('function'/exp OR function)))  Total 498; Clinical article: 120; Case report: 97; clinical trial 43; major clinical study: 36; randomized controlled trial:31; controlled clinical trial 24; prospective study:11; retrospective study:11 |
| **China National Knowledge Infrastructure (CNKI)** |
| [MeSH Terms]: 胸段硬膜外阻滞；心交感神经阻滞；胸段硬膜外镇痛；硬膜外麻醉；硬膜外镇痛；心交感神经切除术； 心力衰竭、缺血性心脏病、扩张型心肌病、心绞痛、冠状动脉粥样硬化性心脏病，左心室射血分数，左心室功能 |
| #1"硬膜外阻滞"[MeSH Terms] [Title/Abstract] 153 publications |
| #2"心交感神经阻滞"[MeSH Terms] [Title/Abstract] 5 publications |
| #3"硬膜外麻醉" [MeSH Terms] [Title/Abstract] 48 publications |
| #4"胸段硬膜外阻滞" [MeSH Terms] [Title/Abstract]和"心力衰竭" [MeSH Terms] [Title/Abstract] 16 publications |
| #5"胸段硬膜外阻滞" [MeSH Terms] [Title/Abstract] 和"缺血性心脏病" [MeSH Terms] [Title/Abstract] 4 publicationss |
| #6"胸段硬膜外阻滞" [MeSH Terms] [Title/Abstract] 和"扩张型心肌病" [MeSH Terms] [Title/Abstract] 14 publications |
| #7"胸段硬膜外阻滞" [MeSH Terms] [Title/Abstract] 和"心绞痛" [MeSH Terms] [Title/Abstract] 42 publications |
| #8"胸段硬膜外阻滞" [MeSH Terms] [Title/Abstract] 和"冠状动脉粥样硬化性心脏病" [MeSH Terms] [Title/Abstract] 0 publications |
| #9"胸段硬膜外阻滞" [MeSH Terms] [Title/Abstract] 和"左心室射血分数" [MeSH Terms] [Title/Abstract] 0 publication |
| #10"胸段硬膜外阻滞" [MeSH Terms] [Title/Abstract] 和"左心室功能" [MeSH Terms] [Title/Abstract] 0 publications |
| #11"心交感神经阻滞" [MeSH Terms] [Title/Abstract] 和"心力衰竭" [MeSH Terms] [Title/Abstract] 0 publications |
| #12"心交感神经阻滞" [MeSH Terms] [Title/Abstract] 和"缺血性心脏病" [MeSH Terms] [Title/Abstract] 0 publication |
| #13"心交感神经阻滞" [MeSH Terms] [Title/Abstract] 和"扩张型心肌病" [MeSH Terms] [Title/Abstract] 1 publications |
| #14"心交感神经阻滞" [MeSH Terms] [Title/Abstract] 和"心绞痛" [MeSH Terms] [Title/Abstract] 0 publications |
| #15"心交感神经阻滞" [MeSH Terms] [Title/Abstract] 和"冠状动脉粥样硬化性心脏病" [MeSH Terms] [Title/Abstract] 0 publications |
| #16"心交感神经阻滞"[MeSH Terms] [Title/Abstract] 和"左心室射血分数" [MeSH Terms] [Title/Abstract] 0 publications |
| #17"心交感神经阻滞" [MeSH Terms] [Title/Abstract] 和"左心室功能" [MeSH Terms] [Title/Abstract] 0 publications |
| #18"硬膜外麻醉" [MeSH Terms] [Title/Abstract]和"心力衰竭" [MeSH Terms] [Title/Abstract] 23 publication |
| #19"硬膜外麻醉" [MeSH Terms] [Title/Abstract] 和"缺血性心脏病" [MeSH Terms] [Title/Abstract] 9 publications |
| #20"硬膜外麻醉" [MeSH Terms] [Title/Abstract] 和"扩张型心肌病" [MeSH Terms] [Title/Abstract] 1 publication |
| #21"胸段硬膜外阻滞" [MeSH Terms] [Title/Abstract] 和"心绞痛" [MeSH Terms] [Title/Abstract] 42 publications |
| #22"硬膜外麻醉" [MeSH Terms] [Title/Abstract] 和"冠状动脉粥样硬化性心脏病" [MeSH Terms] [Title/Abstract] 0 publications |
| #23"硬膜外麻醉" [MeSH Terms] [Title/Abstract] 和"左心室射血分数" [MeSH Terms] [Title/Abstract] 0 publication |
| #24"硬膜外麻醉" [MeSH Terms] [Title/Abstract] 和"左心室功能" [MeSH Terms] [Title/Abstract] 3 publications |
| #25"硬膜外镇痛" [MeSH Terms] [Title/Abstract]和"心力衰竭" [MeSH Terms] [Title/Abstract] 0 publication |
| #26"硬膜外镇痛" [MeSH Terms] [Title/Abstract] 和"缺血性心脏病" [MeSH Terms] [Title/Abstract] 0 publications |
| #24"硬膜外镇痛" [MeSH Terms] [Title/Abstract] 和"扩张型心肌病" [MeSH Terms] [Title/Abstract] 0 publication |
| #27"硬膜外镇痛" [MeSH Terms] [Title/Abstract] 和"心绞痛" [MeSH Terms] [Title/Abstract] 6 publications |
| #28"硬膜外镇痛" [MeSH Terms] [Title/Abstract] 和"冠状动脉粥样硬化性心脏病" [MeSH Terms] [Title/Abstract] 0 publications |
| #29"硬膜外镇痛" [MeSH Terms] [Title/Abstract] 和"左心室射血分数" [MeSH Terms] [Title/Abstract] 0 publication |
| #30"硬膜外镇痛" [MeSH Terms] [Title/Abstract] 和"左心室功能" [MeSH Terms] [Title/Abstract] 0 publications |
| #31"心交感神经切除术" [MeSH Terms] [Title/Abstract]和"心力衰竭" [MeSH Terms] [Title/Abstract] 0 publication |
| #32"心交感神经切除术" [MeSH Terms] [Title/Abstract] 和"缺血性心脏病" [MeSH Terms] [Title/Abstract] 0 publications |
| #33"心交感神经切除术" [MeSH Terms] [Title/Abstract] 和"扩张型心肌病" [MeSH Terms] [Title/Abstract] 0 publication |
| #34"心交感神经切除术" [MeSH Terms] [Title/Abstract] 和"心绞痛" [MeSH Terms] [Title/Abstract] 0 publications |
| #35"心交感神经切除术" [MeSH Terms] [Title/Abstract] 和"冠状动脉粥样硬化性心脏病" [MeSH Terms] [Title/Abstract] 0 publications |
| #36"心交感神经切除术" [MeSH Terms] [Title/Abstract] 和"左心室射血分数" [MeSH Terms] [Title/Abstract] 0 publication |
| #37"心交感神经切除术" [MeSH Terms] [Title/Abstract] 和"左心室功能" [MeSH Terms] [Title/Abstract] 0 publications |

Supplementary Table 2: Transformation of NYHA functional classification in case control studies

| **Author** | **Year** | **HTEB**  **(High thoracic epidural blockade)** | | | | | **CMT**  **(Conventional medical treatment)** | | | | | ***P* for (before)**  **HTEB vs. CMT** | ***P* for (after)**  **HTEB vs. CMT** |
| --- | --- | --- | --- | --- | --- | --- | --- | --- | --- | --- | --- | --- | --- |
|  |  | **Before HTEB** | | **After HTEB** | | ***P* value** | **Before CMT** | | **After CMT** | | ***P* value** |  |  |
|  |  | N | Mean±  SD | n | Mean±  SD |  | N | Mean±  SD | n | Mean±  SD |  |  |  |
| Li, Zhuqin | 2003 | 15 | 3.73±  0.46 | 15 | 1.87±  0.64 | < 0.001 | 15 | 3.67±  0.49 | 15 | 3.27±  0.46 | 0.159 | 0.985 | < 0.001 |
| Li, Zhuqin | 2004 | 30 | 3.57±  0.68 | 30 | 1.90±  0.71 | < 0.001 | 30 | 3.53±  0.68 | 30 | 3.37±  0.72 | 0.792 | 0.998 | < 0.001 |
| Sun, Guifang | 2005 | 41 | 3.3±  0.5 | 41 | 2.3±  0.4 | < 0.001 | 41 | 3.4±  0.6 | 41 | 2.6±  0.3 | < 0.05 | > 0.05 | < 0.05 |
| Wang, Haolian | 2005 | 30 | 3.08±  0.71 | 30 | 2.36±  0.59 | < 0.001 | 30 | 3.07±  0.69 | 30 | 2.71±  0.61 | < 0.05 | > 0.05 | < 0.05 |
| Xiu, Chunhong | 2006 | 23 | 3.87±  0.34 | 23 | 2.13±  0.63 | < 0.001 | 16 | 3.94±  0.25 | 16 | 2.88±  0.62 | < 0.001 | 0.974 | < 0.001 |
| Wang, Xu | 2008 | 16 | 3.81±  0.40 | 16 | 2.00±  0.63 | < 0.001 | 16 | 3.75±  0.45 | 16 | 3.25±  0.68 | 0.062 | 0.989 | <0.001 |
| Liu, Yan | 2008 |  |  | 32 | 1.38±  0.66 |  |  |  | 32 | 2.06±  1.16 |  |  | 0.005 |
| Chi, Hongjie | 2011 | 144 | 3.24±  0.81 | 144 | 1.83±  0.89 | . | 98 | 3.26±  0.96 | 98 | 2.6±  0.79 | . | . | . |
| Li, Shuqing | 2013 | 20 | 3.75±  0.44 | 20 | 2.55±  0.76 | < 0.001 | 20 | 3.65±  0.49 | 20 | 3.30±  0.80 | 0.32 | 0.961 | 0.002 |
| Sun, Guifang | 2017 | 22 | 3.27±  0.46 | 22.00 | 2.09±  0.29 | < 0.001 | 22 | 3.36±  0.49 | 22.00 | 2.41±  0.50 | < 0.001 | 0.905 | 0.09 |

Supplementary Table 3: Transformation of NYHA functional classification in case series studies

| **First author** | **Year** | **Number** | **Pre-HTEB (Mean±SD)** | **Post- HTEB (Mean±SD)** | ***P* value** |
| --- | --- | --- | --- | --- | --- |
| GramlingBabb | 1997 | 10 | 3±1 | 4.5±0.5 | < 0.001 |
| Zhang, Youli | 1999 | 40 | 1.6±0.8 | 3.2±0.8 | < 0.001 |
| Chi, Hongjie | 2002 | 30 | 1.7±0.4 | 3.4±0.5 | < 0.001 |
| Chi, Hongjie (4 weeks) | 2002 | 15 | 1.7±0.5 | 3.2±0.6 | < 0.001 |
| Chi, Hongjie (16 weeks) | 2002 | 15 | 1.5±0.5 | 3.2±0.6 | < 0.001 |
| Chi, Hongjie | 2003 | 9 | 1.8±0.4 | 3.2±0.4 | < 0.001 |
| Li, Zhuqin | 2003 | 18 | 2.2±0.7 | 3.6±0.5 | < 0.001 |
| Li, Zhuqin | 2003 | 10 | 1.8±0.6 | 3.6±0.7 | < 0.001 |
| Chi, Hognjie | 2003 | 14 | 1.8±0.4 | 3.5±0.5 | < 0.001 |
| Wang, Xu | 2007 | 8 | 2.0±0.5 | 3.7±0.4 | < 0.001 |
| Li, Shu (four weeks) | 2015 | 25 | 2.1±0.5 | 3.8±0.4 | < 0.001 |
| Zhao, Min (four weeks) | 2015 | 54 | 2.1±0.5 | 3.8±0.4 | < 0.001 |
| Dong, Hao (atrial fibrillation) | 2016 | 20 | 1.8±0.6 | 3.6±0.5 | < 0.001 |
| Dong, Hao (Non-atrial fibrillation) | 2016 | 20 | 1.9±0.6 | 3.8±0.4 | < 0.001 |
| Zhao, Min | 2016 | 6 | 1.3±0.5 | 3.0±0.6 | < 0.001 |
| Li, Dan (dilated cardiomyopathy) | 2017 | 16 | 2.8±1.3 | 4.5±0.5 | < 0.001 |
| Li, Dan (ischemic cardiomyopathy) | 2017 | 16 | 3.4±1.2 | 4.5±0.5 | < 0.001 |
| Li, Dan (valvar heart disease) | 2017 | 16 | 2.3±1.2 | 4.5±0.5 | < 0.001 |
| Li, Dan | 2017 | 19 | 2.4±1.3 | 4.5±0.5 | < 0.001 |
| Ma, Dan | 2017 | 8 | 1.5±0.5 | 3.6±0.5 | < 0.001 |

**Supplementary Table 4:** Clinical characteristics of trials included in the pooled analysis

| **First author** | **Year** | **Study type/ period** | **Population (NO. of cases, M/F, Age)** | **Disease** |
| --- | --- | --- | --- | --- |
| **Case control studies** | | | | |
| Olausson  [13] | 1997 | Case-control study  Randomized assignment | Total (40, 29/7)/HTEB (18, 15/3)/CMT(18, 14/4), HTEB: 65.1 ± 2.0 y, CMT: 65.0 ± 2.4 y | Angina pectoris due to coronary artery disease |
| Liu, Fengqi [21] | 2001 | Case-control study  Assignment not mentioned | Total (34, 31/3)/HTEB (19, 18/1)/CMT(15, 13/2), HTEB:44.0 ± 11.6 y, CMT: 44.0 ± 21.0 y | Heart failure due to dilated cardiomyopathy |
| Zhu, Lichen [22] | 2001 | Case-control study  Randomized assignment | Total (62, 40/22)/HTEB(31, 19/12)/CMT(31, 21/10), HTEB: 59 ± 16 y, CMT: 62 ± 15 y | Acute coronary syndrome |
| Jin, Xiaoye [23] | 2001 | Case-control study  Randomized assignment | Total (50)/HTEB(25)/CMT(25) | Unstable pectoris due to coronary artery disease |
| Li, Zhuqin  [24] | 2003 | Case-control study  Randomized assignment | Total (30)/HTEB(15)/CMT(15), 22/8, 43 ± 8 y | Heart failure due to dilated cardiomyopathy |
| Li, Zhuqin  [25] | 2004 | Case-control study  Randomized assignment | Total (60 )/HTEB(30)/CMT (30), 47/13, 44.1 ± 11.6 y | Heart failure due to dilated cardiomyopathy |
| Sun, Guifang [26] | 2005 | Case-control study  Randomized assignment | Total (82)/HTEB(41)/CMT (41), 62/20, 52.3 ± 16.4 y | Heart failure due to dilated or/and ischemic cardiomyopathy |
| Wang, Haolian [27] | 2005 | Case-control study  Randomized assignment | Total (60)/HTEB(30 )/CMT (30), 36/24, 54 ± 11.7 y | Heart failure due to rheumatic heart disease, dilated cardiomyopathy, hypertension, coronary artery disease |
| Xiu, Chunhong [28] | 2005 | Case-control study  Randomized assignment | Total (32, 23/9)/HTEB(18, 13/5)/CMT (14,10/4), HTEB: 48 ± 12 y, CMT:51 ± 19 y | Heart failure due to dilated cardiomyopathy |
| Sun, Guifang [29] | 2005 | Case-control study  Randomized assignment | Total (43, 25/18)/HTEB(22, 13/9)/CMT(22, 12/9), HTEB: 48 ±7 y, CMT: 50 ± 8 y | Heart failure due to myocardial infarction |
| Chen, Shulin [30] | 2006 | Case-control study  Randomized assignment | Total (30)/HTEB(15)/CMT (15), 18/12, range: 25 to 58 y | Heart failure due to dilated cardiomyopathy |
| Xiu, Chuhong [31] | 2006 | Case-control study  Randomized assignment | Total (39, 30/9)/HTEB(23, 18/5) /CMT(16, 12/4), HTEB: 51 ± 13 y, CMT: 53 ± 16 y | Heart failure due to dilated cardiomyopathy |
| Xiu, Chunhong [32] | 2006 | Case-control study  Randomized assignment | Total (43, 33/10)/HTEB (20, 17/3)/CMT(23, 16/7), HTEB: 51 ± 12 y, CMT: 52 ± 16 y | Heart failure due to dilated cardiomyopathy |
| Wang, Tao  [33] | 2006 | Case-control study  randomized assignment | Total (60)/HTEB(30)/CMT(30), 46/14, 48 ± 7 y | Refractory angina pectoris due to coronary artery disease |
| Wu, Shuang [34] | 2007 | Case-control study  Randomized assignment | Total (117, 90/27)/HTEB (63, 51/12)/CMT(54, 39/15), 90/27, HTEB: range 61 ± 8 y, CMT: range 48 to 79 y | Heart failure due to ischemic cardiomyopathy |
| Chang, Yulin [35] | 2007 | Case-control study  Randomized assignment | Total (40, 0/40)/HTEB(0/20)/ CMT(0/20),  HTEB: 29.7 ± 4.6 y, CMT: 29.2 ± 4.8 y | Heart failure due to peripartum cardiomyopathy |
| Wang, Xu  [36] | 2008 | Case-control study  Randomized assignment | Total (32, 22/10)/HTEB(16, 12/4)/CDI(16, 10/6), HTEB: 54 ± 18 y, CMT: 55 ± 17 y | Heart failure due to ischemic cardiomyopathy |
| Liu, Yan  [37] | 2008 | Case-control study  Randomized assignment | Total (64)/HTEB(32)/CMT(32), 36/28, mean: 65 y (range: 49 to 82 y) | Angina pectoris due to coronary artery disease |
| Cui, Yuqiu  [38] | 2008 | Case-control study  Randomized assignment | Total (196, 126/70)/HTEB(98, 64/34)/CMT(98, 62/36), HTEB: 60 ± 12 y; CMT: 61 ± 11 y | Unstable pectoris due to coronary artery disease |
| Yi, Hongzhang [39] | 2007 | Case-control study  Randomized assignment | Total (40)/HTEB(20)/CMT(20), 21/19, range: 48 to 79 y | Myocardial infarction due to coronary artery disease |
| Pan, Juping [40] | 2011 | Case-control study  Randomized assignment | Total (40, 25/15)/HTEB (20, 13/7)/CMT(20, 12/8), HTEB: 56 ± 12 y; CMT: 54 ± 10 y | Heart failure due to ischemic cardiomyopathy |
| Chi, Hongjie [13] | 2011 | Case-control study  Randomized assignment | Total (242)/HTEB(144)/CMT(98) | Heart failure due to idiopathic dilated cardiomyopathy |
| Li, Qingshu [41] | 2013 | Case-control study  Randomized assignment | Total (40, 32/8)/HTEB(20,17/3)/CMT(20,15/5),  HTEB: 70 to 60 y (mean 69 y); CMT: mean 68 y (range: 78 to 58 y) | Heart failure due to dilated and ischemic cardiomyopathy, and hypertension |
| Wang, X  [42] | 2014 | Case-control study  Retrospective | Total (30, 22/8)/HTEB (16, 12/4)/CMT (14,10/4); HTEB:54 ± 18 y, CMT: 55 ± 17 y | Heart failure due to ischemic cardiomyopathy |
| Sun, Guifang [43] | 2017 | Case-control study  Randomized assignment | Total (44, 30/14)/HTEB (22, 14/8)/CMT (22, 16/6), HTEB: 51.9 ± 10.0 y, CMT: 53.1 ± 9.5 y | Heart failure due to dilated and ischemic cardiomyopathy. |
| **Case series studies** | | | | |
| Blomberg  [44] | 1989 | Case series study  Self-control | 9 (8/1), 67 ± 3 y | Severe coronary artery disease and unstable angina pectoris |
| Blomberg  [45] | 1989 | Case series study  Self-control | 28 (24/4), 61 ± 2 y (range:36 to 78 y) | Previous acute myocardial infarction and/or angina pectoris and severe coronary artery disease |
| Kock  [46] | 1990 | Case series study  Self-control | 10 (6/4), 58 ± 3y | Stress-induced myocardial ischemia of coronary artery disease |
| Gramling:Babb [47] | 1997 | Case series study  Self-control | 10 (8/2), 58 ± 5 y | Heart failure due to coronary artery disease and AMI |
| Lian, Qizhou [48] | 1998 | Case series study  Self-control | 15(9/6), 58.6 ± 6.9 y | Unstable angina pectoris |
| Lian, Qizhou [49] | 1999 | Case series study  Self-control | 23 of 46 (-/-), - | Unstable angina pectoris |
| Liu, Fengqi [50] | 1999 | Case series study  Self-control | 15 of 31 (20/11), mean 67 y (range:44 to 74 y) | Heart failure due to coronary artery disease |
| Zhang, Youli [51] | 1999 | Case series study  Self-control | 40 (22/18), mean 65 y (range: 60 to 75 y) | Heart failure due to coronary artery disease |
| Liu, Feng  [52] | 2000 | Case series study  Self-control | 12 (10/2), range:44 to 78 y | Unstable angina pectoris |
| Cao, Maorong [53] | 2000 | Case series study  Self-control | 12 (5/7), range:56 to 72 y | Unstable angina pectoris |
| Xing, Shujun [54] | 2000 | Case series study  Self-control | 34 (20/14), mean 67 y (range:44 to 74) | Heart failure due to coronary artery disease |
| Jin, Ruiling [55] | 2000 | Case series study  Self-control | 12 (8/4), 52 ± 12.6 y (range: 30 to 68 y) | Heart failure due to dilated cardiomyopathy |
| Chen,Guozhong[56] | 2001 | Case series study  Self-control | 61 (46/15), 61.6 ± 15.2 y (range: 40 to 82 y) | Refractory angina pectoris |
| Liu, Jie  [57] | 2001 | Case series study  Self-control | 91 (78/13), range: 12 to 68 y | Heart failure due to dilated cardiomyopathy |
| Chi, Hongjie [58] | 2002 | Case series study  Self-control | 30 (25/5), 43.6 ± 11.1 y | Heart failure due to dilated cardiomyopathy |
| Yang, Dejun [59] | 2002 | Case series study  Self-control | 32 (19/13), range: 49 to 70 y | Refractory angina |
| Chi, Hongjie [60] | 2002 | Case series study  Self-control | 15 (14/1), 38. 7 ± 11. 7 y (range: 14 to 63 y) | Heart failure due to dilated cardiomyopathy |
| Yu, Huijun  [61] | 2003 | Case series study  Self-control | 22 (15/7), 62 ± 9 y (range: 52 to70 y) | Refractory angina |
| Cai, Aihong [62] | 2003 | Case series study  Self-control | 21 (15/6), 36 ± 8.07 y (range: 27 to 49 y) | Heart failure due to dilated cardiomyopathy |
| Chi, Hongjie [63] | 2003 | Case series study  Self-control | 9 (7/2), 44±9 y (range: 30 to 63 y) | Heart failure due to Keshan disease (Endemic cardiomyopathy, ECD) |
| Li, Zhuqin  [64] | 2003 | Case series study  Self-control | 18 (18/NA), 45 ± 8 y (range: 34 to 63 y) | Heart failure due to alcoholic cardiomyopathy |
| Li, Zhuqin  [65] | 2003 | Case series study  Self-control | 10 (9/1),-- | Heart failure due to familial dilated cardiomyopathy |
| Chi, Hognjie [66] | 2003 | Case series study  Self-control | 14 (12/2), 64.5 ± 3.6 y (range: 61 to 70 y) | Heart failure due to dilated cardiomyopathy |
| Liu, Huafei  [67] | 2004 | Case series study  Self-control | 40 (24/16), 66.4 ± 7.0 y (range: 54 to 73 y) | Refractory angina |
| Schmidt  [68] | 2005 | Case series study  Self-control | 33 of 37 (26/7), range: 67 to 7 y | Coronary heart disease scheduled for coronary artery bypass surgery |
| Lagunilla  [69] | 2006 | Case series study  Self-control | 25 of 50 (22/3), 66.08 ± 8.28 y | Coronary artery disease and previous myocardial infarction scheduled coronary revascularization |
| Wang, Xu  [70] | 2007 | Case series study  Self-control | 8 (5/3), range: 55 to 74 y | Severe heart failure post-PCI for acute myocardial infarction |
| JAKOBSEN [71] | 2009 | Case series study  Self-control | 15 (NA/NA), mean 62 y (range: 51 to 75 y) | Multi vessel coronary artery disease elective CABG |
| Wang, Xiaowei [72] | 2010 | Case series study  Self-control | 30 (21/9), 56.53 ± 8.52 y (range: 37 to74) | Intractable heart failure due to diabetic cardiomyopathy |
| Wang, Guizhen [73] | 2013 | Case series study  Self-control | 34 (22/12), mean 42 y | Heart failure due to dilated cardiomyopathy with type II diabetes |
| Li, Shu  [74] | 2015 | Case series study  Self-control | 25 (16/9), 55.1 ± 13.4 y (range: 23 to 82 y) | Heart failure due to dilated cardiomyopathy, hypertension and ischemic heart disease |
| Zhao, Min  [75] | 2015 | Case series study  Self-control | 54 (38/16), 56 ± 12.6 y (range: 29 to 82 y) | Heart failure due to dilated cardiomyopathy, valve heart disease, peripartum cardiomyopathy, noncompaction of ventricular myocardium ( NVM), and ischemic heart disease |
| Dong, Hao  [76] | 2016 | Case series study  Self-control | AF group: 20/15/5; NFA group: 20/10/10, AF group: 53.00 ± 10.85, NFA group: 52.20±10.19 y | Heart failure with or without atrial fibrillation (AF) |
| Zhao, Min  [77] | 2016 | Case series study  Self-control | 7 (4/3), range: 40 to 65 y | Hypertrophic cardiomyopathy |
| Ma, Dan  [78] | 2016 | Case series study  Self-control | 82 (-/-), -- | Chronic heart failure |
| Li, Dan  [79] | 2017 | Case series study  Self-control | DCM: 16/11/5); ICM: 16/10/6); VHD:16/12/4), 48.53 ± 13.22 y (range: 24 to 72 y) | Heart failure due to dilated cardiomyopathy (DCM), ischemic heart disease (ICM), and valve heart disease (VHD) |
| Li, Dan  [80] | 2017 | Case series study  Self-control | 18 of 19 (10/9), 48.2 ± 13.5 y (range: 26 to 78 y) | Heart failure due to valvular heart diseases |
| Ma, Dan  [81] | 2017 | Case series study  Self-control | 8 (6/2), 57 ± 6 y | Heart failure due to dilated cardiomyopathy |

**Acronym or Abbreviations:** HTEB = high thoracic epidural blockade; CMT= conventional medical treatment

**Supplementary Table 5:** HTEB intervention

| **First author** | **Year** | **Aesthetics** | **Dosage** | **Frequency** | **Duration** | **Segment** | **Method for assessing the effectiveness of HTBE in terms of neuronal block** | **Conventional medical treatment** |
| --- | --- | --- | --- | --- | --- | --- | --- | --- |
| **Case control studies** | | | | | | | | |
| Olausson  [13] | 1997 | 5 mg/mL bupivacaine | 20-30 mg | A continuous epidural infusion of bupivacaine (7.5 to 16 mg/h) | 48 hours | T1-T5 | Cold discrimination with ice (Extent of loss of temperature discrimination ability) | β-blockers, calcium antagonists, aspirin, heparin, and nitroglycerin infusion |
| Liu, Fengqi [21] | 2001 | 1% Lidocaine | 5-8 ml | Q2H/24 h | 4-8 weeks | T1-T5 | Electronic skin thermometers was used to assess the effectiveness of sympathetic blockade, the skin temperature at the midpoint of the line connecting the two nipples increases by 0.5 to 2.5℃ following the blockade. | furosemide, digoxin, sodium nitroprusside, dopamine |
| Zhu, Lichen [22] | 2001 | 0.75% bupivacaine 0.2 mg | Micro-infusion pump for continuous injection (2 ml/h) | Continuous | 5-7 days | not mentioned | Not mentioned evaluation method. HTEB treatment was associated with a rapid relief of clinical symptoms | nitrates, β-blockers, calcium antagonists, ACEI, anticoagulants |
| Jin, Xiaoye [23] | 2001 | 0.5% Lidocaine | 5-7 ml | Q2-4H/24 h | 5 days | T1-T5 | Not mentioned evaluation method. HTEB treatment was associated with a rapid relief of clinical symptoms | β-blockers, ACEI, nitrates, antiplatelet |
| Li, Zhuqin  [24] | 2003 | 0.5% Lidocaine | 3-5 ml | Q2-4H/24 h | 4 weeks | T1-T5 | Not mentioned evaluation method. HTEB treatment was associated with a rapid relief of clinical symptoms | sodium nitroprusside, dopamine; digitalis, diuretics |
| Li, Zhuqin  [25] | 2004 | 0.5% Lidocaine | 3-5 ml | Q2H/24 h | 4 weeks | T1-T5 | Not mentioned evaluation method. HTEB treatment was associated with a rapid relief of clinical symptoms | cardiac tonic, diuretics, vasodilators |
| Sun, Guifang [26] | 2005 | 0.5% Lidocaine | 4-6 ml | Q2H (9:00 a.m. to 11: 00 p.m.) | 4 weeks | T1-T5 | Not mentioned evaluation method. HTEB treatment was associated with a rapid relief of clinical symptoms | digitalis, diuretics, sodium nitroprusside, β-blockers, digoxin |
| Wang, Haolian [27] | 2005 | 0.5% Lidocaine | 3-5 ml | Q2H or Q4H /24 h | 4 weeks | T1-T5 | Not mentioned evaluation method. HTEB treatment was associated with a rapid relief of clinical symptoms | sodium nitroprusside, ACEIs, digitalis, nitroprusside, diuretics |
| Xiu, Chunhong [28] | 2005 | 0.5% Lidocaine | 3-5 ml | Q2H/24 h | 4 weeks | T1-T5 | Not mentioned evaluation method. HTEB treatment was associated with a rapid relief of clinical symptoms | digitalis, diuretics, vasodilator |
| Sun, Guifang [29] | 2005 | 0.5% Lidocaine | 5 ml | Q2H/24 h | 15, 30, 60 minutes | T1-T5 | Not mentioned evaluation method. HTEB treatment was associated with a rapid relief of clinical symptoms | sodium nitroprusside, dopamine, diuretics, aspirin, low molecular weight heparin |
| Chen, Shulin [30] | 2006 | 0.5% Lidocaine | 5 ml | Q2H/24 h | 2-3 weeks | T1-T5 | Not mentioned evaluation method. HTEB treatment was associated with a rapid relief of clinical symptoms | β-blockers, cardiac tonic, diuretics |
| Xiu, Chuhong [31] | 2006 | 0.5% Lidocaine | 3-5 ml | Q2H/24 h | 4 weeks | T1-T5 | Not mentioned evaluation method. HTEB treatment was associated with a rapid relief of clinical symptoms | sodium nitroprusside, dopamine, digitalis, diuretics |
| Xiu, Chunhong [32] | 2006 | 0.5% Lidocaine | 3-5 ml | Q2H /24 h, | 4 weeks | T1-T5 | Not mentioned evaluation method. HTEB treatment was associated with a rapid relief of clinical symptoms | not mentioned |
| Wang, Tao  [33] | 2006 | 1% Lidocaine and 0.125% bupivacaine | Micro-infusion pump for continuous injection (2ml/h) | Continuous | 48 hours | T1-T5 | Not mentioned evaluation method. HTEB treatment was associated with a rapid relief of clinical symptoms | nitrates, β-blockers, calcium antagonists, nitroglycerin |
| Wu, Shuang [34] | 2007 | 0.5% Lidocaine | 3-5 ml | Q2H (daytime) | 4 weeks | T1-T5 | Not mentioned evaluation method. HTEB treatment was associated with a rapid relief of clinical symptoms | sodium nitroprusside, β-blockers, ACEIs/ARBs, digitalis, diuretics |
| Chang, Yulin [35] | 2007 | 0.5% Lidocaine | Micro-infusion pump for continuous injection (2 ml/hour) | Continuous | 1 week | T1-T5 | Not mentioned evaluation method. HTEB treatment was associated with a rapid relief of clinical symptoms | digitalis, diuretics, aminophylline |
| Wang, Xu  [36] | 2008 | 0.5% Lidocaine | 3-5 ml | Q2H/24h | 4 weeks | T1-T5 | Pupil constriction, conjunctival congestion, eyelid ptosis, and elevated skin temperature. | cardiac tonic, diuretics, vasodilators |
| Liu,Yan  [37] | 2008 | 0.5% Lidocaine | 3-5 ml or 6-8 ml | Q1.5H//24 h | 16-22 days | T1-T5 | Not mentioned evaluation method. HTEB treatment was associated with a rapid relief of clinical symptoms | cardiac tonic, calcium antagonists, vasodilator, nitrates, β-blockers |
| Cui,Yuqiu  [38] | 2008 | 0.5% Lidocaine | 5-7 ml | Q4H-Q6H//24 h | 4-8 weeks | T1-T5 | Electronic skin thermometers was used to assess the effectiveness of sympathetic blockade | nitrates, β-blockers, calcium antagonists, antiplatelet, anticoagulants |
| Yi, Hongzhang [39] | 2007 | 0.1% Ropivacaine | 4-6 ml | 2mL/h | 1, 2, 7days | T1-T5 | Not mentioned evaluation method. HTEB treatment was associated with a rapid relief of clinical symptoms | nitrates, aspirin, statin |
| Pan, Juping [40] | 2011 | 0.5% Lidocaine | 3 ml | Q3-4H/24 h | 2 weeks | not mentioned | Not mentioned evaluation method. HTEB treatment was associated with a rapid relief of clinical symptoms | vasodilators, ACEI, diuretics, digitalis, aldosterone antagonists |
| Chi, Hongjie [13] | 2011 | 0.5% Lidocaine | 5 ml | Q2H//24 h | 4 weeks | T1-T5 | Not mentioned evaluation method. HTEB treatment was associated with a rapid relief of clinical symptoms d | conventional medical therapy |
| Li, Qingshu [41] | 2013 | 0.5% Lidocaine | 5 ml | Q2H (9:00 a.m. to 11: 00 p.m.) | 4 weeks | T1-T5 | Cold discrimination with ice | digoxin, diuretics, β-blockers, ACEs |
| Wang, X  [42] | 2014 | 0.5% Lidocaine | 3-5 ml | Q2H/24 h | 4 weeks | T1-T5 | The temperature at the anesthesia area was recorded using a skin thermometer. In these regions, the temperature may rise by 0.5 to 1.0°C. The signs of successful blockage included miosis, conjunctival hyperemia, ptosis, and skin temperature increase. | sodium nitroprusside, dopamine, cedilanid, and furosemide |
| Sun, Guifang [43] | 2017 | 0.5% Lidocaine | 4 ml | Q2H (9:00 a.m. to 11: 00 p.m.) | 4 weeks | T1-T5 | Not mentioned evaluation method. HTEB treatment was associated with a rapid relief of clinical symptoms | β-blockers, ACEIs/ARBs, spironolactone, digoxin, loop diuretic |
| **Case series studies** | | | | | | | | |
| Blomberg [44] | 1989 | 5 mg/mL Bupivacaine | 4.3 ± 0.2 ml | Bolus epidural injection | 12 hour | T1-T8 | Extent of loss of temperature discrimination ability, using ether applied to the skin of the chest wall | β-blockers, calcium antagonists, long/short acting nitrates, salicylates, heparin |
| Blomberg [45] | 1989 | 2-5 or 5 mg ml^-1^ Bupivacaine intermittent epidural bolus injections of bupivacaine | 4.4 ± 0.3 ml | 2.7± 0.3 to 0.9± 0.3 times daily | 6.0 ± 1.1 days (1 week) | T1-T7 | Ether was used to test the ability to discriminate temperature | β-blockers, calcium antagonists, long/short acting nitrates, salicylates, heparin |
| Kock  [46] | 1990 | 5 mg/ml bupivacaine | 1-2 ml | 10 min | 20-30 min | T1-T5 | Extent of loss of temperature discrimination ability | β-blockers, Calcium channel blockers, Long-acting nitrates |
| Gramling:  Babb  [47] | 1997 | 0.25% to 0.5% Bupivacaine | Continuous infusion or an intermittent rebolus (2.5 to 5.0 mg/ml) | Q4H/24 h | 7-90 days | T1-T5 | Sensory changes in the appropriate dermatome at T2 through T5 and by alleviation of anginal symptoms | nitrates, β-blockers, calcium channel blockers, heparin, ACE inhibitor, narcotics, intraaortic balloon pump |
| Lian, Qizhou [48] | 1998 | 0.5% Lidocaine | 6 ml | Q4H/24 h | 15 min | T1-T5 | Electronic skin thermometers was used to assess the effectiveness of sympathetic blockade | heparin |
| Lian, Qizhou [49] | 1999 | 0.5% Lidocaine | 5-7 ml | Q4-6H/24 h | 3, 7 days | T1-T5 | Electronic skin thermometers was used to assess the effectiveness of sympathetic blockade | heparin |
| Liu, Fengqi [50] | 1999 | 0.5% to 0.75% Lidocaine | 5-8 ml | Q2H or Q4H /24 h | 1week | T1-T5 | Not mentioned evaluation method. HTEB treatment was associated with a rapid relief of clinical symptoms | cediran (or digitalis) , diuretics, vasodilators |
| Zhang, Youli [51] | 1999 | 0.5% to 0.75% Lidocaine | 6-8 ml | Q2H or Q4H /24 h | (2-4 weeks) 15 to 28 days | T1-T5 | Not mentioned evaluation method. HTEB treatment was associated with a rapid relief of clinical symptoms | aspirin |
| Liu, Feng [52] | 2000 | 0.5% Bupivacaine | 3-5ml | Q4-6H/24 h | 5-14 days | T1-T5 | Electronic skin thermometers was used to assess the effectiveness of sympathetic blockade | not mentioned |
| Cao, Maorong [53] | 2000 | 0.5% to 0.75% Lidocaine | 5-8ml | Q4-6H/24 h | not mentioned | T1-T5 | Not mentioned | not mentioned |
| Xing, Shujun [54] | 2000 | 0.125% Bupivacaine | Micro-infusion pump for continuous injection (5-8 ml, 3-5ml/h) | 30 min/1mL | 1 week | T1-T5 | Not mentioned evaluation method. HTEB treatment was associated with a rapid relief of clinical symptoms | cediran (or digitalis) , diuretics, vasodilators prior to HTEB |
| Jin, Ruiling [55] | 2000 | 0.5% Lidocaine | 3-5 ml | Q2-4H/24 h | 2-3 weeks | T1-T5 | Not mentioned evaluation method. HTEB treatment was associated with a rapid relief of clinical symptoms | digitalis, calcium antagonists, nitroglycerin, cediran, diuretics, sodium nitroprusside |
| Chen,Guozhong  [56] | 2001 | 0.25% Bupivacaine | 4-6 ml | 4-6 times/24 hours | 30 min, 24 hours, 1weeks (7 days), 2 weeks (14 days) | T1-T5 | Not mentioned evaluation method. HTEB treatment was associated with a rapid relief of clinical symptoms | nitrates, calcium antagonists, β-blockers |
| Liu, Jie  [57] | 2001 | 0.5% Lidocaine or 0.125% Bupivacaine | 2-5 ml or 2-4 ml | Q2-4H or Q3-5H/24 h | (4-8 weeks) 1 to 2 months | T1-T5 | Not mentioned evaluation method. HTEB treatment was associated with a rapid relief of clinical symptoms | digitalis, diuretics, vasodilators |
| Chi, Hongjie [58] | 2002 | 0.5% Lidocaine | 3-5 ml | Q2H/24 h | 4-8 weeks | T1-T5 | Not mentioned evaluation method. HTEB treatment was associated with a rapid relief of clinical symptoms | conventional drugs |
| Yang, Dejun [59] | 2002 | 1% Lidocaine | 4 ml | 3-5 times/24 hours | 4-50 days | T1-T5 | Not mentioned evaluation method. HTEB treatment was associated with a rapid relief of clinical symptoms | nitrates, calcium antagonists, β-blockers, thrombolytic drugs |
| Chi, Hongjie [60] | 2002 | 0.5% Lidocaine | 3-5 ml | Q2H/24 h | 4 weeks | T1-T5 | Not mentioned evaluation method. HTEB treatment was associated with a rapid relief of clinical symptoms | conventional drugs |
| Yu, Huijun  [61] | 2003 | 0.5% Lidocaine | 3-5 ml | Q2-4H/24 h | 1week | T1-T5 | Not mentioned evaluation method. HTEB treatment was associated with a rapid relief of clinical symptoms | aspirin, heparin, nitroglycerin, β-blockers, calcium antagonists |
| Cai, Aihong [62] | 2003 | 0.5% Lidocaine or 0.12% Ropivacaine | 5-8 ml or 3ml | Q2-4H or Q3H/24 h | 1-4 weeks | T1-T5 | Not mentioned evaluation method. HTEB treatment was associated with a rapid relief of clinical symptoms | conventional drugs, prior to HTEB intervention |
| Chi, Hongjie [63] | 2003 | 0.5% Lidocaine | 3-5 ml | Q2H/24 h | 4-6 weeks | T1-T5 | Not mentioned evaluation method. HTEB treatment was associated with a rapid relief of clinical symptoms | sodium nitroprusside, dopamine, cediran, diuretics |
| Li, Zhuqin  [64] | 2003 | 0.5% Lidocaine | 3-5 ml | Q2-4H/24 h | 4-8 weeks | T1-T5 | Not mentioned evaluation method. HTEB treatment was associated with a rapid relief of clinical symptoms | digitalis, diuretics, vasodilators |
| Li, Zhuqin  [65] | 2003 | 0.5% to 1% Lidocaine | 5-8 ml | Q2H/24 h | 4-8 weeks | T1-T5 | Not mentioned evaluation method. HTEB treatment was associated with a rapid relief of clinical symptoms | digitalis, diuretics, vasodilators |
| Chi, Hognjie [66] | 2003 | 0.5% Lidocaine | 3-5 ml | Q2H/24 h | 4-6 weeks | T1-T5 | Not mentioned evaluation method. HTEB treatment was associated with a rapid relief of clinical symptoms | sodium nitroprusside, dopamine, cediran, diuretics |
| Liu, Huafei [67] | 2004 | 0.5% Lidocaine | 5 ml | Q4H (5-8 times) | 5-28 days | T1-T5 | Not mentioned evaluation method. HTEB treatment was associated with a rapid relief of clinical symptoms | nitrates |
| Schmidt  [68] | 2005 | 0.5% Bupivacaine | 6.0 ± 1.0 ml, 0.075 ml/kg | bolus epidural injection | 30 min | T1-T5 | Temperature and pinprick discrimination | β-blockers; calcium entry blockers, long acting nitrates, angiotensin-converting enzyme inhibitors. |
| Lagunilla  [69] | 2006 | 0.3% Ropivacaine | 5-10 ml | 1 time | 20 min | T1-T2 | Hemithoraces by the pinprick method | morphine, diazepam, intramuscular scopolamine, β-blockers |
| Wang, Xu  [70] | 2007 | 0.5% Lidocaine | 3-5 ml | Q2H/24 h | 4 weeks | T1-T5 | Not mentioned evaluation method. HTEB treatment was associated with a rapid relief of clinical symptoms | conventional drugs |
| JAKOBSEN [71] | 2009 | 2% Lidocaine + 0.5% Bupivacaine | 3 ml+7ml | bolus epidural injection | 20 min | T1-T5 | Regional loss of cold sensation of  the corresponding dermatomes | β-blockers; calcium entry blockers, long acting nitrates, angiotensin-converting enzyme inhibitors, diazepam. |
| Wang, Xiaowei [72] | 2010 | 0.5% Lidocaine | 3-5 ml | Q2H/24 h | 4 weeks (21 to 78 days, 43.0 ± 13.7) | T1-T5 | Not mentioned evaluation method. HTEB treatment was associated with a rapid relief of clinical symptoms | conventional drugs |
| Wang, Guizhen [73] | 2013 | 0.2% Ropivacaine | 3-5 ml | Q4H/24 h | 4-6 weeks | T1-T5 | Not mentioned evaluation method. HTEB treatment was associated with a rapid relief of clinical symptoms | digitalis, diuretics, vasodilators |
| Li, Shu  [74] | 2015 | 0.5% Lidocaine or 0.2% Ropivacaine | 5 ml or 5 ml | Q2H or Q4H /7:00 a.m.-23:00 p.m. | 2 weeks, 4 weeks | T1-T5 | Not mentioned evaluation method. HTEB treatment was associated with a rapid relief of clinical symptoms | sodium nitroprusside, dopamine, cediran, diuretics |
| Zhao, Min  [75] | 2015 | 0.5% Lidocaine or 0.2% Ropivacaine | 5 ml or 5 ml | Q2H or Q4H /24 h | 2 weeks, 4weeks | T1-T5 | Not mentioned evaluation method. HTEB treatment was associated with a rapid relief of clinical symptoms | sodium nitroprusside, dopamine, cediran, diuretics, ACEI/ARB, spironolactone |
| Dong, Hao  [76] | 2016 | 0.5% Lidocaine or 0.2% Ropivacaine | 5 ml or 5 ml | Q2H or Q4H /24 h | 4 weeks | T1-T5 | Not mentioned evaluation method. HTEB treatment was associated with a rapid relief of clinical symptoms | ACEIs/ARBs, β-blockers, spironolactone, cediran, sodium nitroprusside, anticoagulants |
| Zhao, Min  [77] | 2016 | 0.5% Lidocaine or 0.2% Ropivacaine | 5 ml or 5 ml | Q2H or Q4H /24 h | 4 weeks | T1-T5 | Not mentioned evaluation method. HTEB treatment was associated with a rapid relief of clinical symptoms | β-blockers, calcium antagonists |
| Ma, Dan  [78] | 2016 | not mentioned | not mentioned | not mentioned | 4 weeks | not mentioned | Not mentioned evaluation method. HTEB treatment was associated with a rapid relief of clinical symptoms | not mentioned |
| Li, Dan  [79] | 2017 | 0.5% Lidocaine | 5 ml | Q2H/24 h | 4 weeks | T1-T5 | Not mentioned evaluation method. HTEB treatment was associated with a rapid relief of clinical symptoms | conventional drugs |
| Li, Dan  [80] | 2017 | 0.5% Lidocaine | 5 ml | Q2H/24 h | 4 weeks | T1-T5 | Not mentioned evaluation method. HTEB treatment was associated with a rapid relief of clinical symptoms | nitroprusside sodium, dopamine, digitalis and furosemide, angiotensin converting enzyme inhibitors/angiotensin receptor blockers (ACEI/ARB) and spironolactone. |
| Ma, Dan  [81] | 2017 | 0.5% Lidocaine | 5 ml | Q2H/24 h | 4 weeks | T1-T5 | Skin temperature | β-blockers, ACEI/ARB, diuretics, spironolactone, aspirin, nitrates, statins, digitalis |

**Supplementary Table 6:** Methodology quality assessment of case-control trials included in the analysis using modified Jadad score (7 point)

| **Study ID** | **randomization** | | | **Concealment of allocation** | | | **Double blinding** | | | **Withdrawals and dropouts** | |
| --- | --- | --- | --- | --- | --- | --- | --- | --- | --- | --- | --- |
| Items | Not randomized or inappropriate method of randomization | The study was described as randomized | The method of randomization was described and it was appropriate | Not described the method of allocation | The study was described as using allocation concealment methods | The method of allocation concealment was described appropriately | No blind or inappropriate method of blinding | The study was described as double blind | The method of double blind was described and it was appropriate | Not describe the follow-up | A description of withdrawals and dropouts |
| Score |  | 1 | 2 | 0 | 1 | 2 | 0 | 1 | 2 | 0 | 1 |
| Olausson 1997 [13] |  | 1 |  | 0 |  |  | 0 |  |  | 0 |  |
| Liu, Fengqi 2001 [21] |  |  |  | 0 |  |  | 0 |  |  | 0 |  |
| Zhu, Lichen 2001[22] |  | 1 |  | 0 |  |  | 0 |  |  | 0 |  |
| Jin, Xiaoye 2001 [23] |  | 1 |  | 0 |  |  | 0 |  |  | 0 |  |
| Li, Zhuqin 2003 [24] |  | 1 |  | 0 |  |  | 0 |  |  | 0 |  |
| Li, Zhuqin 2004 [25] |  | 1 |  | 0 |  |  | 0 |  |  | 0 |  |
| Sun, Guifang 2005[26] |  | 1 |  | 0 |  |  | 0 |  |  | 0 |  |
| Wang, Haolian 2005 [27] |  | 1 |  | 0 |  |  | 0 |  |  | 0 |  |
| Xiu, Chunhong 2005[28] |  | 1 |  | 0 |  |  | 0 |  |  | 0 |  |
| Sun, Guifang 2005 [29] |  | 1 |  | 0 |  |  | 0 |  |  | 0 |  |
| Chen, Shulin 2006 [30] |  | 1 |  | 0 |  |  | 0 |  |  | 0 |  |
| Xiu, Chuhong 2006 [31] |  | 1 |  | 0 |  |  | 0 |  |  | 0 |  |
| Xiu, Chunhong 2006 [32] |  | 1 |  | 0 |  |  | 0 |  |  | 0 |  |
| Wang, Tao 2006 [33] |  | 1 |  | 0 |  |  | 0 |  |  | 0 |  |
| Wu, Shuang 2007 [34] |  | 1 |  | 0 |  |  | 0 |  |  | 0 |  |
| Chang, Yulin 2007 [35] |  | 1 |  | 0 |  |  | 0 |  |  | 0 |  |
| Wang, Xu 2008 [36] |  | 1 |  | 0 |  |  | 0 |  |  | 0 |  |
| Liu,Yan 2008 [37] |  | 1 |  | 0 |  |  | 0 |  |  | 0 |  |
| Cui, Yuqiu 2008 [38] |  | 1 |  | 0 |  |  | 0 |  |  | 0 |  |
| Yi, Hongzhang 2007 [39] |  | 1 |  | 0 |  |  | 0 |  |  | 0 |  |
| Pan, Juping  2011[40] |  | 1 |  | 0 |  |  | 0 |  |  | 0 |  |
| Chi, Hongjie 2011[13] |  | 1 |  | 0 |  |  | 0 |  |  | 0 |  |
| Li, Qingshu 2013 [41] |  |  | 2 |  |  | 2 |  |  | 2 | 0 |  |
| Wang, X 2014 [42] | 0 |  |  | 0 |  |  | 0 |  |  | 0 |  |
| Sun, Guifang 2017 [43] |  |  | 2 | 0 |  |  | 0 |  |  | 0 |  |

**Supplementary Table** 7 Estimating literature quality in individual case-control study using Newcastle-Ottawa Scale

| **Bias domain** | **Signalling question** | **Response options** | **Studies** |
| --- | --- | --- | --- |
| **Selection** | 1.Representativeness of the exposed cohort | (1) Truly representative (one star); (2) Somewhat representative (one star); (3) Selected group; (4) No description of the derivation of the cohort | (1) Olausson 1997; (1) Liu, fengqi 2001; (1) Zhu, lichen 2001; (1) Jin, xiaoye2001; (1) Li, zhuqin 2003; (1) Li, zhuqin 2004; (1) Sun, guifang 2005; (1) Wang, haolian 2005; (1) Xiu, chunhong 2005; (1) Sun, guifang 2005; (1) Chen, shulin 2006; (1) Xiu, chunhong 2006; (1) Xiu, chunhong 2006; (1) Wang, tao 2006; (1) Wu, shuang 2007; (1) Yi, hongzhang 2007; (1) Chang, yulin 2007; (1) Wang, xu 2008; (1) Liu, yan 2008; (1) Cui, yuqiu 2008; (1) Pan, juping 2011; (1) Chi, hongjie 2011; (1) Li, qingshu 2013; (1) Wang, xu 2014; (1) Sun, guifang 2017. |
|  | 2.Selection of the non-exposed cohort | (1) Drawn from the same community as the exposed cohort (one star); (2) Drawn from a different source; (3) No description of the derivation of the non-exposed cohort | 1. Olausson 1997; (1) Liu, fengqi 2001; (1) Zhu, lichen 2001; (1) Jin, xiaoye 2001; (1) Li, zhuqin 2004; (1) Sun, guifang 2005; (1) Wang, haolian 2005; (1) Xiu, chunhong 2005; (1) Sun, guifang 2005; (1) Chen, shulin 2006; (1) Xiu, chunhong 2006; (1) Xiu, chunhong 2006; (1) Wang, tao 2006; (1) Wu, shuang 2007; (1) Yi, hongzhang 2007; (1) Chang, yulin 2007; (1) Wang, xu 2008; (1) Liu, yan 2008; (1) Cui, yuqiu 2008; (1) Pan, juping 2011; (1) Chi, hongjie 2011; (1) Li, qingshu 2013;(1) Wang, xu 2014; (1) Sun, guifang 2017. |
|  | 3. Ascertainment of exposure | (1) Secure record (e.g., surgical record) (one star); (2) Structured interview (one star); (3)Written self-report; (4) No description; (5)Other | (1) Olausson 1997, (1)Liu, fengqi 2001, (1) Zhu, lichen 2001; (1)Jin, xiaoye 2001; (1) Li, zhuqin 2003; (1) Li, zhuqin 2004; (1) Sun, guifang 2005; (1) Wang, haolian 2005; (1) Xiu, chunhong 2005; (1) Sun, guifang 2005; (1) Chen, shulin 2006; (1) Xiu, chunhong 2006; (1) Xiu, chunhong 2006; (1) Wang, tao 2006; (1) Wu, shuang 2007; (1) Yi, hongzhang 2007; (1) Chang, yulin 2007; (1) Wang, xu 2008; (1) Liu, yan 2008; (1) Cui, yuqiu 2008; (1) Pan, juping 2011; (1) Chi, hongjie 2011; (1) Li, qingshu 2013; (1) Wang, xu 2014; (1) Sun, guifang 2017. |
|  | 4.Demonstration that outcome of interest was not present at start of study | (1)Yes (one star); (2) No | (1) Olausson 1997; (1) Liu, fengqi 2001; (1) Zhu, lichen 2001; (1) Jin, xiaoye 2001; (1) Li, zhuqin 2003; (1) Li, zhuqin 2004; (1) Sun, guifang 2005; (1) Wang, haolian 2005; (1) Xiu, chunhong 2005; (1) Sun, guifang 2005; (1) Chen, shulin 2006; (1) Xiu, chunhong 2006; (1) Xiu, chunhong 2006; (1) Wang, tao 2006; (1) Wu, shuang 2007; (1) Yi, hongzhang 2007; (1) Chang, yulin 2007; (1) Wang, xu 2008; (1) Liu, yan 2008; (1) Cui, yuqiu 2008; (1) Pan, juping 2011; (1) Chi, hongjie 2011; (1) Li, qingshu2013; (1) Wang, xu 2014; (1) Sun, guifang 2017. |
| **Comparability** | Comparability of cohorts on the basis of the design or analysis controlled for confounders | (1) The study controls for age, sex and marital status (one star); (2) Study controls for other factors (list) __baseline values of indicators and NYHA functional classification__one star); (3) Cohorts are not comparable on the basis of the design or analysis controlled for confounders | (1) Olausson 1997; (2) Liu, fengqi 2001; (2) Zhu, lichen 2001; (2) Jin, xiaoye 2001; (2) Li, zhuqin 2003; (2) Li, zhuqin 2004; (2) Sun, guifang 2005; (2) Wang, haolian 2005; (2) Xiu, chunhong 2005; (2) Sun, guifang 2005; (2) Chen, shulin 2006; (2) Xiu, chunhong 2006; (2) Xiu, chunhong 2006; (2) Wang, tao 2006; (2) Wu, shuang 2007; (2) Yi, hongzhang 2007; (2) Chang, yulin 2007; (2) Wang, xu 2008; (2) Liu, yan 2008; (2) Cui, yuqiu 2008; (2) Pan, juping 2011; (2) Chi, hongjie 2011; (2) Li, qingshu2013; (2) Wang, xu 2014; (2) Sun, guifang 2017. |
| **Outcome** | 1. Assessment of outcome | (1) Independent blind assessment (one star); (2) Record linkage (one star); (3) Self-report; (4) No description ; (5) Other | (2) Olausson 1997; (2) Liu, fengqi 2001; (2) Zhu, lichen 2001; (2) Jin, xiaoye 2001; (2) Li, zhuqin 2003; (2) Li, zhuqin 2004; (2) Sun, guifang 2005; (2) Wang, haolian 2005; (2) Xiu, chunhong 2005; (2) Sun, guifang 2005; (2) Chen, shulin 2006; (2) Xiu, chunhong 2006; (2) Xiu, chunhong 2006; (2) Wang, tao 2006; (2) Wu, shuang 2007; (2) Yi, hongzhang 2007; (2) Chang, yulin 2007; (2) Wang, xu 2008; (2) Liu, yan 2008; (2) Cui, yuqiu 2008; (2) Pan, juping 2011; (2) Chi, hongjie 2011; (1) Li, qingshu2013; (2) Wang, xu 2014; (2) Sun, guifang 2017. |
|  | 2.Was follow-up long enough for outcomes to occur | (1)Yes (one star); (2) No Indicate the median duration of follow-up and a brief rationale for the assessment above: ___. | (1) Chihongjie 2011. |
|  | 3.Adequacy of follow-up of cohorts | (1) Complete follow up- all subject accounted for (one star); (2) Subjects lost to follow up unlikely to introduce bias- number lost less than or equal to 20% or description of those lost suggested no different from those followed (one star); (3) Follow up rate less than 80% and no description of those lost; (4) No statement | (1) Chihongjie 2011. |

**Supplementary Table 8** Total score of Newcastle-Ottawa Scale (NOS) in individual case-control studies

| **Study ID** | **Represent-**  **ativeness**  **of the exposed cohort** | **Selection of the non-exposed cohort** | **Ascertai-nment of exposure** | **Demonstration that outcome of interest was not present at start of study** | **Comparability of cohorts on the basis of the design or analysis controlled for confounders** | **Assessment of outcome** | **Was follow-up**  **long enough for**  **outcomes to occur** | **Adequacy of**  **follow-up**  **of cohorts** | **Total**  **score** |
| --- | --- | --- | --- | --- | --- | --- | --- | --- | --- |
| Olausson 1997 [13] | ★ | ★ | ★ | ★ | ★ | ★ |  |  | 6 |
| Liu, Fengqqi 2001 [21] | ★ | ★ | ★ | ★ | ★ | ★ |  |  | 6 |
| Zhu, Lichen 2001 [22] | ★ | ★ | ★ | ★ | ★ | ★ |  |  | 6 |
| Jin, Xiaoye 2001 [23] | ★ | ★ | ★ | ★ | ★ | ★ |  |  | 6 |
| Li, Zhuqin 2003 [24] | ★ | ★ | ★ | ★ | ★ | ★ |  |  | 6 |
| Li, Zhuqin 2004 [25] | ★ | ★ | ★ | ★ | ★ | ★ |  |  | 6 |
| Sun, Guifang 2005 [26] | ★ | ★ | ★ | ★ | ★ | ★ |  |  | 6 |
| Wang, Haolian 2005 [27] | ★ | ★ | ★ | ★ | ★ | ★ |  |  | 6 |
| Xiu, Chunhong 2005 [28] | ★ | ★ | ★ | ★ | ★ | ★ |  |  | 6 |
| Sun, Guifang 2005 [29] | ★ | ★ | ★ | ★ | ★ | ★ |  |  | 6 |
| Chen, Shulin 2006 [30] | ★ | ★ | ★ | ★ | ★ | ★ |  |  | 6 |
| Xiu,Chunhong 2006 [31] | ★ | ★ | ★ | ★ | ★ | ★ |  |  | 6 |
| Xiu,Chunhong 2006 [32] | ★ | ★ | ★ | ★ | ★ | ★ |  |  | 6 |
| Wang,Tao 2006 [33] | ★ | ★ | ★ | ★ | ★ | ★ |  |  | 6 |
| Wu,Shuang 2007 [34] | ★ | ★ | ★ | ★ | ★ | ★ |  |  | 6 |
| Chang,Yulin 2007 [35] | ★ | ★ | ★ | ★ | ★ | ★ |  |  | 6 |
| Wang, Xu  2008 [36] | ★ | ★ | ★ | ★ | ★ | ★ |  |  | 6 |
| Liu, Yan  2008 [37] | ★ | ★ | ★ | ★ | ★ | ★ |  |  | 6 |
| Cui, Yuqiu 2008 [38] | ★ | ★ | ★ | ★ | ★ | ★ |  |  | 6 |
| Yi, Hongzhang 2007 [39] | ★ | ★ | ★ | ★ | ★ | ★ |  |  | 6 |
| Pan, Uuping 2011 [40] | ★ | ★ | ★ | ★ | ★ | ★ |  |  | 6 |
| Chi, Hongjie 2011 [13] | ★ | ★ | ★ | ★ | ★ | ★ | ★ | ★ | 8 |
| Li, Qingshu 2013 [41] | ★ | ★ | ★ | ★ | ★ | ★ |  |  | 6 |
| Wang, Xu  2014 [42] | ★ | ★ | ★ | ★ | ★ | ★ |  |  | 6 |
| Sun, Guifang 2017 [43] | ★ | ★ | ★ | ★ | ★ | ★ |  |  | 6 |

**Supplementary Table 9** IHE items

| **Checklist and draft dictionary (Delphi process)** |
| --- |
| 1.Is the hypothesis/aim/objective of the study clearly stated? **Yes**: The hypothesis/aim/objective of the study is clearly reported. **Unclear**: The hypothesis/aim/objective of the study is vague or unclearly reported. **No**: The hypothesis/aim/objective is not reported. |
| 2.Are the characteristics of the participants included in the study described ? **Yes:** The most relevant characteristics of the participants are reported (e.g. the total number, age, and gender distribution). Ethnicity, severity of disease/condition, comorbidity, or etiology should also be included, if relevant. **Partially reported**: Only the number of participants was reported. **No**: None of the relevant characteristics of the participants is reported. |
| 3.Were the cases collected in more than one centre? **Yes**: Cases are collected in more than one centre (multicentre study). **Unclear**: Unclear where the patients come from (i.e. single or multicentre study). **No**: Cases are collected from one centre. |
| 4. Are the eligibility criteria (i.e. inclusion and exclusion criteria) for entry into the study clearly stated? **Yes**: Both inclusion and exclusion criteria are reported. **Partially reported**: Only one, the inclusion or exclusion criteria is reported. **No**: Neither inclusion nor exclusion criteria are reported. |
| 5. Were participants recruited consecutively? **Yes**: There is a clear statement or it is clear from the context that the participants were recruited consecutively or study stated that all eligible patients were recruited. **Unclear**: The method used to recruit participants is not clearly stated or no information is provided about the method used to recruit participants in the study. **No**: The cases studied were a subgroup of those treated with no evidence to show that they were selected consecutively. The participants were recruited based on other criteria such as access to intervention determined by the distance or availability of resources. |
| 6. Did participants enter the study at a similar point in the disease? **Yes**: There is a clear description about all participants entering the study at a similar point in the condition/disease based on their clinical status, duration of condition or exposure before the intervention, severity of disease, and presence of co-morbidities or complications. **Unclear**: There is no description of the characteristics of participants before entering the study or there is no statement about entering the study at a similar point in the disease. **No**: Participants did not enter the study at a similar point in the condition/disease. This can be revealed by a wide range of disease durations before entering the study or different levels of severities or comorbidities or complications due to progression of their condition/disease. |
| 7. Was the intervention of interest clearly described? **Yes**: The most relevant characteristics of the intervention are reported (e.g. dosage, frequency of administration, duration, permanent or temporary intervention, technical parameters/ characteristics of a device). **Partially reported**: Intervention is only mentioned by name. **No**: None of the relevant characteristics of the intervention was reported |
| 8. Were additional interventions (co-interventions) reported in the study? **Yes**: Participants received additional co-intervention(s). **Unclear**: It is suspected that a co-intervention was administered but the information is not reported. **No**: There is a clear statement or it is clear from the context that a co-intervention was not administered. |
| 9. Are the outcome measures established a priori? **Yes**: All relevant outcome measures are reported in the introduction or methods section (e.g. accomplished, measurable improvements or effects, symptoms relieved, improved function, improved test scores, and quality of life measures). **Partially reported**: Some of the relevant outcomes are briefly reported in the introduction or methods section. **No**: The outcome measures are reported for the first time in the results, discussion, or conclusion section of the study. |
| 10. Were the relevant outcomes measured with appropriate objective and/or subjective methods? **Yes:** All relevant outcomes are measured with appropriate methods which are described in the methods section. These measures might be objective (e.g. gold standard tests or standardized clinical tests), subjective (e.g. self-administered questionnaires, standardized forms, or patient symptoms interview forms), or both. **Unclear**: It is unclear how the relevant outcomes were measured. No information is provided on the methods used to measure study’s relevant outcomes. **No**: The methods used to measure outcomes were inappropriate |
| 11. Were the relevant outcomes measured before and after the intervention? **Yes**: The relevant outcomes are measured before and after applying the intervention.  **Unclear**: It is unclear when the outcomes were measured. **No**: The study reported only outcomes measured after applying the intervention. |
| 12. Were the statistical tests used to assess the relevant outcomes appropriate?  **Yes**: The statistical tests are clearly described in the methods section of the study and are used appropriately (e.g. parametric test for normally distributed population vs. nonparametric test for non Gaussian population). The reviewer should assign a yes score if no statistical analysis was performed but reasons for this were stated. **Unclear:** The statistical tests are not described in the methods section of the study or there is no information about the statistical analysis. **No**: The statistical tests were used inappropriately. |
| 13. Was the length of follow-up reported? **Yes**: The length of follow-up is clearly reported (mean, median, range, standard deviation). **Unclear**: The duration of follow-up is not clearly reported. **No**: The length of follow-up is not reported. |
| 14. Was the loss to follow-up reported? **Yes**: The number or proportion of participants lost to follow-up is clearly reported or authors report outcome results on all participants included initially, or number lost to follow-up can be subtracted from the number enrolled and number analyzed. **Unclear**: It is not clear from the information provided how many participants were lost to follow-up or it is an inconsistence of reporting lost to follow-up (e.g. discrepancies between information from tables and text). **No**: The number or proportion of participants lost to follow-up is not reported. |
| 15. Does the study provide estimates of the random variability in the data analysis of relevant outcomes? **Yes**: The study reports estimates of the random variability (e.g. standard error, standard deviation, confidence interval for parametric data, and range and interquartile range for nonparametric data) for all relevant outcomes.  **Unclear or partially reported**: The presentation of the random variability is unclear (e.g.; the measure of dispersion is reported without indicating if it is a standard deviation or standard error). Estimates of the random variability are not reported for all relevant outcomes. **No**: The study does not report estimates of the random variability |
| 16. Are the adverse events related with the intervention reported? **Yes**: The undesirable or unwanted consequences of the intervention during the study period or within a prespecified time period are reported. The absence of adverse event(s) is acknowledged in the study. **Partially reported**: It is deducible that only some but not all potential adverse events are reported. **No**: There is no statement about the presence or absence of adverse events. |
| 17. Are the conclusions of the study supported by results? **Yes**: The conclusions of the study (in terms of patient, intervention, outcomes) are supported by the evidence presented in the results and discussion sections. **Partially reported**: Not all components of the patient, intervention, outcomes are supported by the evidence presented in the results and discussion section. **No**: The conclusions are not supported by the evidence presented in the results and discussion section. |
| 18. Are both competing interests and sources of support for the study reported? **Yes**: Both competing interests and sources of support (financial or other) received for the study are reported, or the absence of any competing interest and source of support is acknowledged. **Partially reported**: Only one of these elements is reported. **No**: Neither competing interests nor sources of support was reported. |
| 19. Was the study conducted prospectively? **Yes**: It is clearly stated that the study was conducted prospectively. **Unclear**: The design of the study is not mentioned or it is unclear if the study was conducted prospectively. **No**: The authors clearly stated that it was a retrospective study. |
| 20. Were the relevant outcomes assessed blinded to intervention status? **Yes**: The relevant outcomes were analyzed by individuals who were not aware of the intervention status. **Unclear**: The study did not report whether the outcome assessors were aware of the intervention status. **No**: It is clearly stated or obvious that the relevant outcomes were analyzed by individuals who were aware of the intervention status. |

**Supplementary Table 10** IHE estimation

| Study ID | 1 | 2 | 3 | 4 | 5 | 6 | 7 | 8 | 9 | 10 | 11 | 12 | 13 | 14 | 15 | 16 | 17 | 18 | 19 | 20 |
| --- | --- | --- | --- | --- | --- | --- | --- | --- | --- | --- | --- | --- | --- | --- | --- | --- | --- | --- | --- | --- |
| Blomberg  1989 [44] | Yes | Yes | No | Yes | Yes | Yes | Yes | Yes | Yes | Yes | Yes | Yes | No | No | Yes | Yes | Yes | Yes | Unclear | Unclear |
| Blomberg  1989 [45] | Yes | Yes | No | No | Yes | Yes | Yes | Yes | Yes | Yes | Yes | Yes | No | No | Yes | No | Yes | Yes | Unclear | Unclear |
| Kock  1990 [46] | Yes | Yes | No | Yes | Yes | Yes | Yes | Yes | Yes | Yes | Yes | Yes | No | No | Yes | No | Yes | Yes | Unclear | Unclear |
| Gramling:  Babb 1997 [47] | Yes | Yes | Yes | Yes | Yes | Yes | Yes | Yes | Yes | Yes | Yes | Yes | Yes | No | Yes | Yes | Yes | No | Unclear | Unclear |
| Lian, Qizhou  1998 [48] | Yes | Yes | No | No | Yes | Yes | Yes | Yes | Yes | Yes | Yes | Yes | No | No | Yes | No | Yes | No | Unclear | Unclear |
| Lian, Qizhou  1999 [49] | Yes | Yes | No | No | Yes | Yes | Yes | Yes | Yes | Yes | Yes | Yes | No | No | Yes | Yes | Yes | No | Unclear | Unclear |
| Liu, Fengqi  1999 [50] | Yes | Yes | No | No | Yes | Yes | Yes | No | Yes | Yes | Yes | Yes | No | No | Yes | No | Yes | No | Unclear | Unclear |
| Zhang, Youli 1999 [51] | Yes | Yes | No | No | Yes | No | Yes | No | Yes | Yes | Yes | Yes | No | No | Yes | No | Yes | No | Unclear | Unclear |
| Liu, Feng  2000 [52] | Yes | Yes | No | No | Yes | Yes | Yes | No | Yes | Yes | Yes | Yes | No | No | Yes | No | Yes | No | Unclear | Unclear |
| Chao, Maorong 2000 [53] | Yes | Yes | No | Yes | Yes | Yes | Yes | No | Yes | Yes | Yes | Yes | No | No | Yes | No | Yes | Yes | Unclear | Unclear |
| Xing, Shujun 2000 [54] | Yes | Yes | No | No | Yes | Yes | Yes | No | Yes | Yes | Yes | Yes | No | No | Yes | No | Yes | No | Unclear | Unclear |
| Jin, Ruiling  2000 [55] | Yes | Yes | No | No | unclear | No | Yes | Unclear | Yes | Yes | Yes | Yes | No | No | Yes | No | Yes | No | Unclear | Unclear |
| Chen, Guozhong 2001 [56] | Yes | Yes | No | Yes | unclear | Yes | Yes | No | Yes | Yes | Yes | Yes | No | No | Yes | Yes | Yes | No | Unclear | Unclear |
| Liu, Jie  2001 [57] | Yes | Yes | No | Yes | Yes | Yes | Yes | Yes | Yes | Yes | Yes | Yes | Yes | No | Yes | Yes | Yes | No | Unclear | Unclear |
| Chi, Hongjie 2002 [58] | Yes | Yes | No | Yes | Yes | Yes | Yes | Yes | Yes | Yes | Yes | Yes | No | No | Yes | No | Yes | Yes | Unclear | Unclear |
| Yang, Dejun  2002 [59] | Yes | Yes | No | No | unclear | Yes | Yes | Unclear | Yes | Yes | Yes | Yes | No | No | Yes | No | Yes | No | Unclear | Unclear |
| Chi, Hongjie 2002 [60] | Yes | Yes | No | No | Yes | Yes | Yes | Yes | Yes | Yes | Yes | Yes | No | No | Yes | No | Yes | Yes | Unclear | Unclear |
| Yu, Huijun  2003 [61] | Yes | Yes | No | Yes | Yes | Yes | Yes | Yes | Yes | Yes | Yes | Yes | No | No | Yes | Yes | Yes | No | Unclear | Unclear |
| Cai, Aihong  2003 [62] | Yes | Yes | No | No | Yes | Unclear | Yes | No | Yes | Yes | Yes | Yes | No | No | Yes | No | Yes | No | Unclear | Unclear |
| Chi, Hongjie 2003 [63] | Yes | Yes | No | No | unclear | Yes | Yes | Yes | Yes | Yes | Yes | Yes | No | No | Yes | No | Yes | Yes | Unclear | Unclear |
| Li, Zhuqin  2003 [64] | Yes | Yes | No | No | Yes | Yes | Yes | Yes | Yes | Yes | Yes | Yes | No | No | Yes | No | Yes | Yes | Unclear | Unclear |
| Li, Zhuqin  2003 [65] | Yes | Yes | No | No | unclear | No | Yes | Yes | Yes | Yes | Yes | Yes | No | No | Yes | No | Yes | Yes | Unclear | Unclear |
| Chi, Hongjie  2003 [66] | Yes | Yes | No | No | Yes | Yes | Yes | Yes | Yes | Yes | Yes | Yes | Yes | No | Yes | No | Yes | Yes | Unclear | Unclear |
| Liu, Huafei  2004 [67] | Yes | Yes | No | No | unclear | No | Yes | Unclear | Yes | Yes | Yes | Yes | No | No | Yes | No | Yes | No | Unclear | Unclear |
| Schmidt  2005 [68] | Yes | Yes | No | Yes | Yes | Yes | Yes | Yes | Yes | Yes | Yes | Yes | No | No | Yes | No | Yes | Yes | Unclear | Unclear |
| Lagunilla  2006 [69] | Yes | Yes | No | Yes | Yes | Yes | Yes | Yes | Yes | Yes | Yes | Yes | No | No | Yes | No | Yes | Yes | Yes | Yes |
| Wang, Xu  2007 [70] | Yes | Yes | No | No | No | Yes | Yes | Yes | Yes | Yes | Yes | Yes | No | No | Yes | No | Yes | No | Unclear | Unclear |
| JAKOBSEN 2009 [71] | Yes | Yes | No | No | Yes | Yes | Yes | Yes | Yes | Yes | Yes | Yes | No | No | Yes | No | Yes | Yes | Unclear | Yes |
| Wang, Xiaowei  2010 [72] | Yes | Yes | No | No | Yes | Yes | Yes | Yes | Yes | Yes | Yes | Yes | No | No | Yes | No | Yes | Yes | Unclear | Unclear |
| Wang, Guizhen 2013 [73] | Yes | Yes | No | No | Yes | Yes | Yes | Yes | Yes | Yes | Yes | Yes | No | No | Yes | No | Yes | Yes | Unclear | Unclear |
| Li, Shu  2015 [74] | Yes | Yes | No | No | unclear | No | Yes | Yes | Yes | Yes | Yes | Yes | No | No | Yes | No | Yes | Yes | Unclear | Unclear |
| Zhao, Min  2015 [75] | Yes | Yes | No | Yes | Yes | No | Yes | Yes | Yes | Yes | Yes | Yes | Yes | No | Yes | No | Yes | Yes | Unclear | Unclear |
| Dong, Hao  2016 [76] | Yes | Yes | No | Yes | Yes | Yes | Yes | Yes | Yes | Yes | Yes | Yes | No | No | Yes | No | Yes | Yes | Unclear | Unclear |
| Zhao, Min  2016 [77] | Yes | Yes | No | No | unclear | No | Yes | Yes | Yes | Yes | Yes | Yes | Yes | No | Yes | No | Yes | Yes | Unclear | Unclear |
| Ma, Dan  2016 [78] | Yes | No | Unclear | No | unclear | Yes | Yes | Unclear | Yes | Yes | Yes | Yes | No | No | Yes | No | Yes | No | Unclear | Unclear |
| Li, Dan  2017 [79] | Yes | Yes | No | No | Yes | No | Yes | Yes | Yes | Yes | Yes | Yes | No | No | Yes | No | Yes | Yes | Unclear | Unclear |
| Li, Dan  2017 [80] | Yes | Yes | No | Yes | Yes | Yes | Yes | Yes | Yes | Yes | Yes | Yes | No | No | Yes | No | Yes | Yes | Yes | Unclear |
| Ma, Dan  2017 [81] | Yes | Yes | No | Yes | Yes | Yes | Yes | Yes | Yes | Yes | Yes | Yes | No | No | Yes | Yes | Yes | No | Unclear | Unclear |

**Supplementary Table 11** Estimation of HTEB related complications in involved studies

| **Study ID** | **Hypotension** | **Weak** | **Dizzy** | **Infection** | **Respirat-**  **ory**  **depression** | **Bleeding** | **Hemato-**  **mas** | **Thrombo-**  **embolism** | **Paraplegia** | **Cardiac**  **Arrest/ died** | **Local**  **flare** | **Catheter**  **detachment/**  **occlusion** | **Pain** | **Horner's**  **syndrome** | **Urinary retention** |
| --- | --- | --- | --- | --- | --- | --- | --- | --- | --- | --- | --- | --- | --- | --- | --- |
| Olausson et al  1997 [13] | NA | NA | NA | NA | NA | NA | NA | NA | NA | NA | NA | NA | NA | 1 pts* | 10 pts* |
| Liu, Fengqi  2001 [21] | # | # | # | # | # | # | # | # | # | # | # | # | # | # | # |
| Zhu, Lichen  2001 [22] | # | # | # | # | # | # | # | # | # | # | # | # | # | # | # |
| Jin, Xiaoye  2001 [23] | NA | NA | NA | NA | NA | NA | NA | NA | NA | NA | NA | 2 pts* | 2 pts* | NA | NA |
| Li, Zhuqin  2003 [24] | # | # | # | # | # | # | # | # | # | # | # | # | # | # | # |
| Li, Zhuqin  2004 [25] | # | # | # | # | # | # | # | # | # | # | # | # | # | # | # |
| Sun, Guifang  2005 [26] | NA | NA | NA | NA | NA | NA | NA | NA | NA | NA | NA | 2pts* | NA | NA | NA |
| Wang, Haolian  2005 [27] | NA | NA | NA | NA | NA | NA | NA | NA | NA | NA | 2 pts* | NA | NA | NA | NA |
| Xiu, Chunhong  2005 [28] | # | # | # | # | # | # | # | # | # | # | # | # | # | # | # |
| Sun, Guifang  2005 [29] | NA | NA | NA | NA | NA | NA | NA | NA | NA | NA | NA | NA | NA | NA | NA |
| Chen, Shulin  2006 [30] | # | # | # | # | # | # | # | # | # | # | # | # | # | # | # |
| Xiu, Chunhong  2006 [31] | # | # | # | # | # | # | # | # | # | # | # | # | # | # | # |
| Xiu, Chunhong  2006 [32] | # | # | # | # | # | # | # | # | # | # | # | # | # | # | # |
| Wang, Tao  2006 [33] | # | # | # | # | # | # | # | # | # | # | # | # | # | # | # |
| Wu, Shuang  2007 [34] | 7 pts* | 11 pts* | 9 pts* | NA | NA | NA | NA | NA | NA | NA | NA | NA | NA | NA | NA |
| Chang, Yulin  2007 [35] | NA | NA | NA | NA | NA | NA | NA | NA | NA | NA | 1pts* | NA | NA | NA | NA |
| Wang, Xu  2008 [36] | # | # | # | # | # | # | # | # | # | # | # | # | # | # | # |
| Liu, Yan  2008 [37] | # | # | # | # | # | # | # | # | # | # | # | # | # | # | # |
| Cui, Yuqiu  2008 [38] | NA | NA | 1 pts* | NA | NA | NA | NA | NA | NA | NA | 2 pts* | NA | NA | NA | NA |
| Yi, Hongzhang 2007 [39] | # | # | # | # | # | # | # | # | # | # | # | # | # | # | # |
| Pan Juping 2011 [40] | # | # | # | # | # | # | # | # | # | # | # | # | # | # | # |
| Chi, Hongjie  2011 [13] | NA | NA | NA | 1 pts* | NA | NA | NA | NA | NA | NA | NA | NA | NA | 2 pts* | NA |
| Li, Qingshu  2013[41] | NA | NA | NA | 1 pts* | NA | 2 pts* | NA | NA | NA | NA | NA | NA | NA | NA | NA |
| Wang, Xu  2014 [42] | # | # | # | # | # | # | # | # | # | # | # | # | # | # | # |
| Sun, Guifang  2017 [43] | # | # | # | # | # | # | # | # | # | # | # | # | # | # | # |
| Blomberg 1989 [44] | NA | NA | NA | NA | NA | NA | NA | NA | NA | NA | NA | NA | 1 pts* | NA | NA |
| Blomberg 1989 [45] | # | # | # | # | # | # | # | # | # | # | # | # | # | # | # |
| Kock  1990 [46] | # | # | # | # | # | # | # | # | # | # | # | # | # | # | # |
| Gramling:Babb 1997 [47] | NA | NA | NA | 1 pts* | NA | NA | NA | NA | NA | 1 pts | NA | 1 pts* | NA | 1 pts* | NA |
| Lianqizhou  1998 [48] | # | # | # | # | # | # | # | # | # | # | # | # | # | # | # |
| Lianqizhou  1999 [49] | NA | NA | NA | NA | NA | NA | NA | NA | NA | NA | NA | 1 pts* | NA | NA | NA |
| Liu, Fengqi  1999 [50] | # | # | # | # | # | # | # | # | # | # | # | # | # | # | # |
| Zhang, Youli  1999 [51] | # | # | # | # | # | # | # | # | # | # | # | # | # | # | # |
| Liu, Feng  2000 [52] | # | # | # | # | # | # | # | # | # | # | # | # | # | # | # |
| Chao, Maorong  2000 [53] | # | # | # | # | # | # | # | # | # | # | # | # | # | # | # |
| Xing, Shujun  2000 [54] | # | # | # | # | # | # | # | # | # | # | # | # | # | # | # |
| Jin, Ruiling  2000 [55] | # | # | # | # | # | # | # | # | # | # | # | # | # | # | # |
| Chen, Guozhong 2001 [56] | NA | NA | NA | NA | NA | NA | NA | NA | NA | NA | NA | 5 pts* | NA | NA | NA |
| Liu, Jie 2 001 [57] | NA | NA | NA | NA | NA | NA | NA | NA | NA | 2 pts | NA | NA | NA | NA | NA |
| Chi, Hongjie 2002 [58] | # | # | # | # | # | # | # | # | # | # | # | # | # | # | # |
| Yang, Dejun  2002 [59] | # | # | # | # | # | # | # | # | # | # | # | # | # | # | # |
| Chi, Hongjie 2002 [60] | # | # | # | # | # | # | # | # | # | # | # | # | # | # | # |
| Yu, Huijun2003 [61] | NA | NA | NA | NA | NA | NA | NA | NA | NA | NA | NA | NA | NA | NA | NA |
| Cai, Aihong 2003 [62] | # | # | # | # | # | # | # | # | # | # | # | # | # | # | # |
| Chi, Hongjie2003 [63] | # | # | # | # | # | # | # | # | # | # | # | # | # | # | # |
| Li, Zhuqin 2003 [64] | # | # | # | # | # | # | # | # | # | # | # | # | # | # | # |
| Li, Zhuqin 2003 [65] | # | # | # | # | # | # | # | # | # | # | # | # | # | # | # |
| Chi, Hongjie2003 [66] | # | # | # | # | # | # | # | # | # | # | # | # | # | # | # |
| Liu, Huafei 2004 [67] | # | # | # | # | # | # | # | # | # | # | # | # | # | # | # |
| Schmidt 2005 [68] | # | # | # | # | # | # | # | # | # | # | # | # | # | # | # |
| Lagunilla 2006 [69] | # | # | # | # | # | # | # | # | # | # | # | # | # | # | # |
| Wang, Xu 2007 [70] | # | # | # | # | # | # | # | # | # | # | # | # | # | # | # |
| JAKOBSEN e 2009 [71] | # | # | # | # | # | # | # | # | # | # | # | # | # | # | # |
| Wang, Xiaowei2010[72] | # | # | # | # | # | # | # | # | # | # | # | # | # | # | # |
| Wang, Guizhen 2013[73] | # | # | # | # | # | # | # | # | # | # | # | # | # | # | # |
| Li, Shu 2015 [74] | # | # | # | # | # | # | # | # | # | # | # | # | # | # | # |
| Zhao, Min 2015 [75] | # | # | # | # | # | # | # | # | # | # | # | # | # | # | # |
| Dong, Hao 2016 [76] | # | # | # | # | # | # | # | # | # | # | # | # | # | # | # |
| Zhao, Min2016 [77] | # | # | # | # | # | # | # | # | # | # | # | # | # | # | # |
| Ma, Dan 2016 [78] | # | # | # | # | # | # | # | # | # | # | # | # | # | # | # |
| Li, Dan 2017 [79] | # | # | # | # | # | # | # | # | # | # | # | # | # | # | # |
| Li, Dan2017 [80] | # | # | # | # | # | # | # | # | # | # | # | # | # | # | # |
| Ma, Dan 2017 [81] | NA | NA | NA | NA | NA | NA | NA | NA | NA | NA | NA | NA | NA | NA | NA |

Patients (Pts) with * denote the complication related to HTEB procedure, and Pts without* denote the complication is not related to HTEB procedure. # denote there was no report. NA denote that there was no corresponding complication.

**Supplementary Table 12:** Meta-regression analysis of LVEF in case control trials

| **Indicators** | **Coefficient 95% confidence interval** | ***P* > \|t\|** |
| --- | --- | --- |
| **NYHA cardiac functional classification** | | |
| **Multivariate regression (Adjusted R^2^=11.81%)** |  |  |
| Etiology of heart failure | 0.259 (-0.120,0.639) | 0.150 |
| Frequency of HTEB intervention | -0.060 (-0.414,0.295) | 0.703 |
| **Univariate regression** |  |  |
| Etiology of heart failure | 0.239 (-0.091,0.570) | 0.133 |
| Frequency of HTEB intervention | 0.019 (-0.339,0.378) | 0.904 |
| **Left ventricular ejection fraction** |  |  |
| **Multivariate regression (Adjusted R^2^= 19.28%)** |  |  |
| Etiology of heart failure | -0.737(-2.325,0.852) | 0.329 |
| Dose of anesthetic agents | -0.965(-3.543,1.613) | 0.428 |
| Frequency of HTEB intervention | -0.370(-2.030,1.289) | 0.633 |
| Duration of HTEB intervention | 1.057(-5.632,7.747) | 0.734 |
| **Univariate regression** | | |
| Etiology of heart failure | -1.189(-2.318,-0.060) | **0.040** |
| Dose of anesthetic agents | -1.640(-3.434,0.154) | 0.070 |
| Frequency of HTEB intervention | -0.695(-1.831,0.441) | 0.211 |
| Duration of HTEB intervention | -1.298(-6.059,3.462) | 0.568 |

**Supplementary Table 13:** Subgroup analysis of LVEF based on the etiologies of heart failure in case control trials

| **Parameters** | **No. of**  **studies** | **Pooled Weighted Mean Difference**  **(WMD) and 95% Conf. Interval** | ***P* for *WMD*** | **I-squared**  **(*I*^2^, %)** | ***P* for**  **heterogeneity** |
| --- | --- | --- | --- | --- | --- |
| Post-HTEB versus Post-CMT | | | | | |
| Based on the etiology of heart failure | **16** |  |  |  |  |
| Heart failure due to dilated cardiomyopathy | 7 | 10.36 9 (8.184, 12.555) | 0.000 | 33.30% | 0.174 |
| Heart failure due to ischemic cardiomyopathy | 3 | 4.934 (3.138, 6.731) | 0.000 | 0.00% | 0.599 |
| Heart failure due to dilated or and ischemic cardiomyopathy | 2 | 4.870 (3.597, 6.143) | 0.000 | 72.90% | 0.055 |
| Heart failure due to multiple cardiovascular disease | 2 | 2.734 (-0.102, 5.570) | 0.059 | 47.90% | 0.166 |
| Heart failure due to peripartum cardiomyopathy | 1 | 5.000 (-0.332, 10.332) | 0.066 | .% | . |
| Angina pectoris due to coronary artery disease | 1 | 6.000 (1.590, 10.410) | 0.008 | .% | . |

**Supplementary Table 14:** Meta-regression analysis of NT-pro BNP, LVEF and LVEDD in case series studies

| **Indicators** | Coefficient 95% confidence interval | *P* > \|t\| |
| --- | --- | --- |
| **N-terminal pro-brain natriuretic peptide** |  |  |
| **Multivariate regression (Adjusted R^2^= -22.26%)** |  |  |
| Dose of anesthetic agents | 359.502 (-8860.591,9579.595) | 0.932 |
| Frequency of HTEB intervention | 625.943 (-5722.259,6974.145) | 0.828 |
| Duration of HTEB intervention | 593.772(-2637.344,3824.888) | 0.687 |
| **Univariate regression** |  |  |
| Dose of anesthetic agents | 1212.057 (-2337.219,4761.334) | 0.468 |
| Frequency of HTEB intervention | 895.016 (-1536.882,3326.915) | 0.435 |
| Duration of HTEB intervention | 669.618 (-2257.581,3596.817) | 0.625 |
| **Left ventricular ejection fraction** | | |
| **Multivariate regression (Adjusted R^2^= 22.61%)** | | |
| Classification of disease | -2.973(-6.452,0.507) | 0.090 |
| Dose of anesthetic agents | -0.644(-2.696,1.408) | 0.522 |
| Frequency of HTEB intervention | -2.588(-5.418,0.242) | 0.071 |
| Duration of HTEB intervention | 1.008(-0.385,2.400) | 0.148 |
| **Univariate regression** |  |  |
| Classification of disease | -2.342(-5.020,0.335) | 0.084 |
| Dose of anesthetic agents | 0.464(-1.472,2.400) | 0.627 |
| Frequency of HTEB intervention | -0.812(-3.553,1.929) | 0.548 |
| Duration of HTEB intervention | 1.215(-0.002,2.432) | **0.050** |
| **Left ventricular end-dilation dimension** | | |
| **Multivariate regression (Adjusted R^2^= 19.91%)** | | |
| Classification of disease | 1.207(-1.369,3.784) | 0.337 |
| Dose of anesthetic agents | -1.078(-2.553,0.397) | 0.142 |
| Frequency of HTEB intervention | 0.329(-2.642,3.299) | 0.818 |
| Duration of HTEB intervention | 0.195(-1.089,1.479) | 0.753 |
| **Univariate regression** | | |
| Classification of disease | 1.567(-0.478,3.611) | 0.126 |
| Dose of anesthetic agents | -1.389(-2.682,-0.096) | **0.037** |
| Frequency of HTEB intervention | -1.003(-3.693,1.686) | 0.446 |
| Duration of HTEB intervention | 0.143(-1.169,1.455) | 0.823 |

**Supplementary Table 15:** Subgroup analysis of BNP, LVEF, LVEDD in case series studies

| **Parameters** | **No. of**  **studies** | **Pooled Weighted Mean Difference**  **(WMD) and 95% Conf. Interval** | ***P* for *WMD*** | **I-squared**  **(*I*^2^, %)** | ***P* for**  **heterogeneity** |
| --- | --- | --- | --- | --- | --- |
| **Post-HTEB versus Pre-HTEB** |  |  |  |  |  |
| **N-terminal pro-brain natriuretic peptide** | 8 |  |  |  |  |
| Dose of anesthetic agents |  |  |  |  |  |
| 0.5% Lidocaine | 5 | -4.2e+03 (-4.6e+03, -3.7e+03) | 0.000 | 97.7% | 0.000 |
| 0.5 % Lidocaine or 0.2% Ropivacaine | 3 | -3.3e+03 (-4.2e+03, -2.5e+03) | 0.000 | 0.0% | 0.968 |
| Frequency of HTEB intervention | 8 |  |  |  |  |
| Q2H | 5 | -4.2e+03 (-4.6e+03, -3.7e+03) | 0.000 | 97.7% | 0.000 |
| Q2H or Q4H | 2 | -3.5e+03 (-4.4e+03 ,-2.5e+03) | 0.000 | 0.0% | 0.922 |
| Q2H or Q4H (7:00 a.m.-23:00 p.m.) | 1 | -2.8e+03 (-4.6e+03, -918.320) | 0.003 | 0.0% | 0.862 |
| Duration of HTEB intervention | 8 |  |  |  |  |
| 4 weeks | 7 | -4.1e+03 (-4.5e+03, -3.7e+03) | 0.000 | 96.6% | 0.000 |
| 2 weeks | 2 | -3.1e+03 (-4.7e+03, -1.6e+03) | 0.000 | 0.0% | 0.872 |
| 4-8 weeks | 1 | -3.2e+03 (-4.7e+03, -1.8e+03) | 0.000 | .% | **.** |
| **Left ventricular ejection fraction** |  |  |  |  |  |
| Classification of disease | 23 |  |  |  |  |
| Heart failure due to coronary artery disease | 7 | 13.755 (12.183,15.328) | 0.000 | 96.1% | 0.000 |
| Heart failure due to dilated cardiomyopathy | 10 | 9.198 (7.981,10.414) | 0.000 | 49.4% | 0.038 |
| Heart failure due to other cardiovascular diseases | 8 | 7.923 (6.676, 9.171) | 0.000 | 28.3% | 0.175 |
| Dose of anesthetic agents | 23 |  |  |  |  |
| 0.5% Lidocaine (3 to 5 ml) | 8 | 10.791( 9.241,12.341) | 0.000 | 71.6% | 0.001 |
| 0.5 % Lidocaine (5 ml) or 0.2% Ropivacaine (5 ml) | 3 | 6.791 (5.081, 8.501) | 0.000 | 0.0% | 0.591 |
| 0.5% Lidocaine (5 ml) | 4 | 6.589 (4.828, 8.349) | 0.000 | 53.9% | 0.054 |
| Other doses | 8 | 12.350(11.103, 13.598) | 0.000 | 95.0% | 0.000 |
| Frequency of HTEB intervention | 23 |  |  |  |  |
| Q2H or Q4H/24h | 9 | 10.805 (9.766, 11.844) | 0.000 | 93.6% | 0.000 |
| Q2H/24h | 11 | 8.552 (7.339, 9.766 ) | 0.000 | 68.3% | 0.000 |
| Q4H/24h | 1 | 10.700 (6.232, 15.168) | . | .% | 0.000 |
| Others | 2 | 7.720 (3.895,11.544) | 0.000 | 25.1% | 0.248 |
| Duration of HTEB intervention |  |  |  |  |  |
| 4 weeks | 10 | 8.347(7.142, 9.551) | 0.000 | 67.5% | 0.000 |
| 4-8 weeks | 4 | 8.615 (7.286, 9.944) | 0.000 | 0.0% | 0.603 |
| 2 weeks | 2 | 5.183 (2.464, 7.901) | 0.000 | 0.0% | 0.834 |
| 4-6 weeks | 3 | 11.307 (7.797, 14.817) | 0.000 | 0.0% | 0.749 |
| minutes | 2 | 7.720 (3.895, 11.544) | 0.000 | 25.1% | 0.248 |
| Others | 4 | 22.369 (20.034, 24.705) | 0.000 | 93.7% | 0.000 |
| **Left ventricular end-dilation dimension** |  |  |  |  |  |
| Dose of anesthetic agents | 17 |  |  |  |  |
| 0.5% Lidocaine (3-5 ml) | 7 | -4.944 (-6.497,-3.392) | 0.000 | 9.8% | 0.354 |
| 0.5 % Lidocaine (5 ml) or 0.2% Ropivacaine (5 ml) | 3 | -2.677 (-4.485,-0.868) | 0.004 | 0% | 0.999 |
| 0.5% Lidocaine (5 ml) | 4 | -7.151 (-7.902, -6.400) | 0.000 | 0% | 0.630 |
| Others | 3 | -12.059 (-13.35,-10.767) | 0.000 | 83.1% | 0.003 |

**Supplementary Table 16:** Meta-regression analysis of NYHA, LVEF, LVEDD in merged studies

| **Merged studies** | | |
| --- | --- | --- |
| **NYHA cardiac functional classification** | | |
| **Multivariate regression (Adjusted R^2^= 16.43%)** | | |
| Classification of disease | -0.003 (-0.145,0.138) | 0.961 |
| Frequency of HTEB intervention | 0.167 (-0.034,0.367) | 0.100 |
| Duration of HTEB intervention | 0.049 (-0.070,0.168) | 0.407 |
| **Univariate regression** |  |  |
| Dose of anesthetic agents | -0.109(-0.310,0.092) | 0.276 |
| Classification of disease | 0.033(-0.076,0.141) | 0.542 |
| Frequency of HTEB intervention | 0.060(-0.075,0.195) | 0.370 |
| Duration of HTEB intervention | 0.000(-0.107,0.106) | 0.996 |
| **Left ventricular ejection fraction** | | |
| **Multivariate regression (Adjusted R^2^= 13.25%)** | | |
| Classification of disease | 0.120(-0.926,1.165) | 0.818 |
| Dose of anesthetic agents | -0.633(-2.482,1.217) | 0.493 |
| Frequency of HTEB intervention | -0.298(-1.687,1.092) | 0.667 |
| Duration of HTEB intervention | 1.189(0.293,2.086) | **0.011** |
| **Univariate regression** |  |  |
| Classification of disease | 0.341(-0.686,1.368) | 0.506 |
| Dose of anesthetic agents | 0.588(-0.882,2.058) | 0.424 |
| Frequency of HTEB intervention | 0.322(-0.880,1.524) | 0.592 |
| Duration of HTEB intervention | 0.945(0.260,1.630) | **0.008** |
| **Left ventricular end-dilation dimension** | | |
| **Multivariate regression (Adjusted R^2^= 5.88%)** | | |
| Classification of disease | 0.424(-0.441,1.289) | 0.326 |
| Dose of anesthetic agents | -0.951(-2.622,0.721) | 0.256 |
| Frequency of HTEB intervention | 0.284(-0.808,1.375) | 0.601 |
| Duration of HTEB intervention | 0.220(-0.580,1.020) | 0.579 |
| **Univariate regression** |  |  |
| Classification of disease | 0.514(-0.319,1.346) | 0.219 |
| Dose of anesthetic agents | -0.748(-2.147,0.652) | 0.286 |
| Frequency of HTEB intervention | 0.129(-0.841,1.100) | 0.789 |
| Duration of HTEB intervention | 0.093(-0.690,0.875) | 0.811 |

**Supplementary Table 17:** Subgroup analysis of LVEF in merged studies

| **Parameters** | **No. of studies** | **Pooled Weighted Mean Difference**  **(WMD) and 95% Conf. Interval 95% CI** | ***P* for**  **heterogeneity** | ***I*^2^** | ***P* for *WMD*** |
| --- | --- | --- | --- | --- | --- |
| **Left ventricular ejection fraction** | | | | | |
| **Classification of disease** |  |  |  |  |  |
| Heart failure due to dilated cardiomyopathy | 18 | 9.694 (8.655, 10.733) | 0.122 | 28.8% | 0.000 |
| Heart failure due to ischemic heart disease | 4 | 8.372 (6.912, 9.832) | 0.004 | 77.9% | 0.000 |
| Heart failure due to dilated and ischemic cardiomyopathy | 2 | 9.271 (7.974, 10.568) | 0.000 | 97.5% | 0.000 |
| Heart failure due to other cardiomyopathy | 11 | 7.457 (6.348, 8.567) | 0.162 | 27.3% | 0.000 |
| Other coronary artery disease | 6 | 18.639 (16.769, 20.510) | 0.000 | 94.3% | 0.000 |
| **Dose of anesthetic agents** |  |  |  |  |  |
| 0.5% Lidocaine | 27 | 9.271 (8.580, 9.961) | 0.000 | 75.6% | 0.000 |
| 0.5 % Lidocaine or 0.2% Ropivacaine | 3 | 6.791 (5.081, 8.501) | 0.591 | 0.0% | 0.000 |
| 0.5% to 0.75% Lidocaine | 2 | 26.864 (23.904, 29.825) | 0.000 | 95.6% | 0.000 |
| Other doses | 6 | 9.297 (7.930, 10.665) | 0.515 | 0.0% | 0.000 |
| **Frequency of HTEB intervention** |  |  |  |  |  |
| Minutes | 2 | 7.720 (3.895, 11.544) | 0.248 | 25.1% | 0.000 |
| Q2H/24 h | 19 | 8.949 (7.948, 9.949) | 0.001 | 56.8% | 0.000 |
| Q2H (9:00 a.m. to 11: 00 p.m.) | 4 | 8.960 (7.784, 10.135) | 0.000 | 93.6% | 0.000 |
| Q2-4H/24h | 11 | 10.549 (9.550, 11.549) | 0.000 | 92.6% | 0.000 |
| Q3-4H/24h | 1 | 10.000 (7.809, 12.191) | . | .% | 0.000 |
| Others | 2 | 13.803 (10.414, 17.191) | 0.037 | 77.1% | 0.000 |
| **Duration of HTEB intervention** |  |  |  |  |  |
| Minutes | 2 | 7.720 (3.895, 11.544) | 0.248 | 25.1% | 0.000 |
| 1 week | 2 | 8.398 (4.448, 12.348) | 0.257 | 22.1% | 0.000 |
| 2 weeks | 3 | 8.103 (6.397, 9.809) | 0.025 | 72.8% | 0.000 |
| 2-3 weeks | 3 | 16.326 (13.236, 19.417) | 0.590 | 0.0% | 0.000 |
| 4 weeks | 21 | 8.761 (8.002, 9.521) | 0.000 | 74.4% | 0.000 |
| 4-6 weeks | 3 | 11.307 (7.797, 14.817) | 0.749 | 0.0% | 0.000 |
| 4-8 weeks | 5 | 8.697 (7.375, 10.018) | 0.531 | 0.0% | 0.000 |
| Other weeks | 2 | 26.407 (23.512, 29.302) | 0.000 | 96.0% | 0.000 |
